# A Thiophilic Metal-Mediated Cysteine *S*‑Detritylation Strategy: Enhancing Orthogonality in Solid-Phase Peptide Synthesis

**DOI:** 10.1021/acsomega.6c05277

**Published:** 2026-09-25

**Authors:** Salvatore Mottola, Rosa Bellavita, Ida Boccino, Gennaro Maria Bailo, Roberto Cutolo, Mariateresa Giustiniano, Stefania Galdiero, Paolo Grieco, Salvatore Di Maro, Anna Messere, Francesco Merlino

**Affiliations:** † DiSTABiF, University of Campania Luigi Vanvitelli, via Vivaldi 43, 81100 Caserta, Italy; ‡ Department of Pharmacy, 9307 University of Naples Federico II, via Domenico Montesano 49, 80131 Naples, Italy

## Abstract

The selective manipulation of cysteine residues in solid-phase peptide synthesis (SPPS), beyond disulfide bridge formation, remains challenging, and an efficient protocol for the selective removal of the widely used trityl (Trt) protecting group under solid-phase conditions has yet to be established. To overcome this limitation, we developed a thiophilic transition metal-mediated strategy by adapting a three-component reagent system originally conceived for solution-phase synthesis. Systematic screening of copper, zinc and silver salts identified Ag­(CF_3_CO_2_) and AgNO_3_ as the most effective promoters of Trt removal. The optimized protocol enabled reproducible and efficient deprotection, provided preliminary mechanistic insight through control experiments, and proved compatible with common Fmoc-SPPS protecting groups and alternative solvents. Furthermore, *in situ* thiol release enabled direct on-resin *S*-alkylation with a selected range of aliphatic substituents, including those bearing aromatic moieties, highlighting the utility of the method for the late-stage functionalization of cysteine-containing resin-bound peptides.

## Graphical abstract

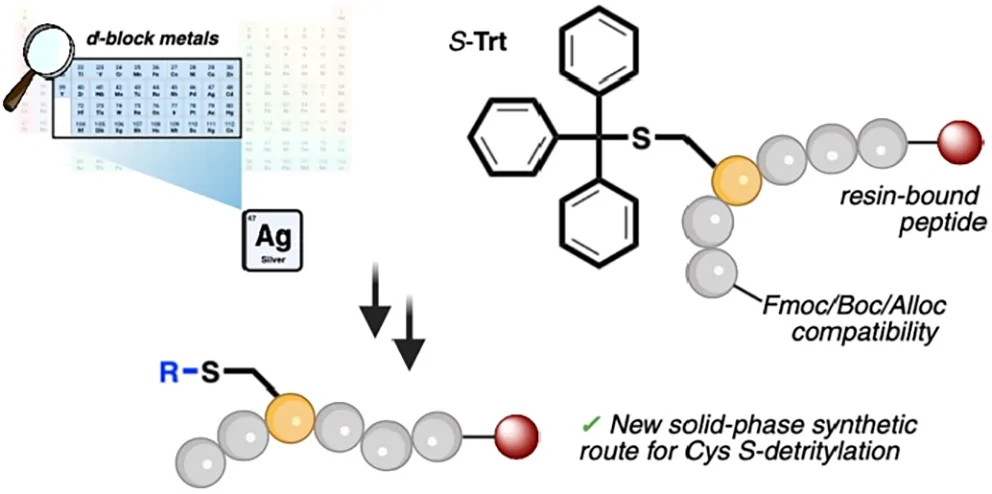

## Introduction

Cysteine (Cys) sulfhydryl group (-SH) is a pertinent site of derivatization for modulating the biological profile of numerous proteins and peptides.^1–4^ Therefore, synthetic procedures for thiol-selective modifications, including *S*-alkylation (*e*.*g*., farnesylation, PEGylation, glycosylation, *etc*.),^5^ disulfide bond formation,^6^ chemical ligation,^7^ and others referring to -SH manipulations,^1^ can be employed to achieve peptides and/or peptidomimetics suitable for biological and/or biotechnological investigations.^2^

The fluorenylmethoxycarbonyl (Fmoc)/*tert*-butyl (*t*-Bu)-based solid phase peptide synthesis (SPPS) represents the chemical method of choice for peptide synthesis that, beyond elongation, offers numerous opportunities for site-selective functionalization through orthogonal protecting group strategies.^8–10^ In the specific case of cysteine thiol, which can be otherwise subjected to unwanted reactions such as acylation, alkylation or oxidation,^4^ diverse protecting groups have been introduced.^11–13^ Among those highly sensitive to acidic conditions,^11–16^ the triphenylmethyl (trityl, Trt), originally introduced in 1962 as protecting group for solution-phase peptide synthesis,^17^ is now routinely used also in the Fmoc-based SPPS for protecting the Cys residue during the construction of linear sequences and disulfide-rich peptides.^3–6^ The starting *N* ^α^-Fmoc-protected reagent [*e*.*g*., Fmoc-Cys­(Trt)] is the most widely available and cheapest form in the market (Table S1, Supporting Information), and it is easily found in all SPPS-equipped laboratories.

*S*-detritylation typically uses acidic conditions, either with Lewis acids (LAs), such as AgNO_3_, HgCl_2_, Hg­(OAc)_2_, CuSO_4_, CuCl, Cu­(OTf)_2_, or BF_3_·Et_2_O in solution-phase systems,^17–21^ or diluted Brønsted acids (BAs), such as trifluoroacetic acid (TFA), HCl, HBr or mixed acetic acid (AcOH)/HBr systems,^10,11,17^ in both solution- and solid-phase systems, resulting in LA- or BA-catalyzed hydrolysis, respectively. Due to the high stability of the Trt cation and the strong nucleophilicity of the thiol group, complete deprotection requires the addition of a scavenger [e.*g*., triisopropylsilane (TIS) or triethylsilane (TES)] to the reaction mixture, as commonly implemented in SPPS *cleavage cocktails* (Figure 1a).^8,13,22^ Trt cation is thus effectively scavenged and irreversibly converted into triphenylmethane.^13,23^ Depending on the protocol, additional reagents may be required to suppress side reactions or maintain cysteine in its reduced state (*e*.*g*., ethanedithiol), whereas oxidative procedures such as iodinolysis lead directly to disulfide formation.^11,15,24^

**Figure 1:**
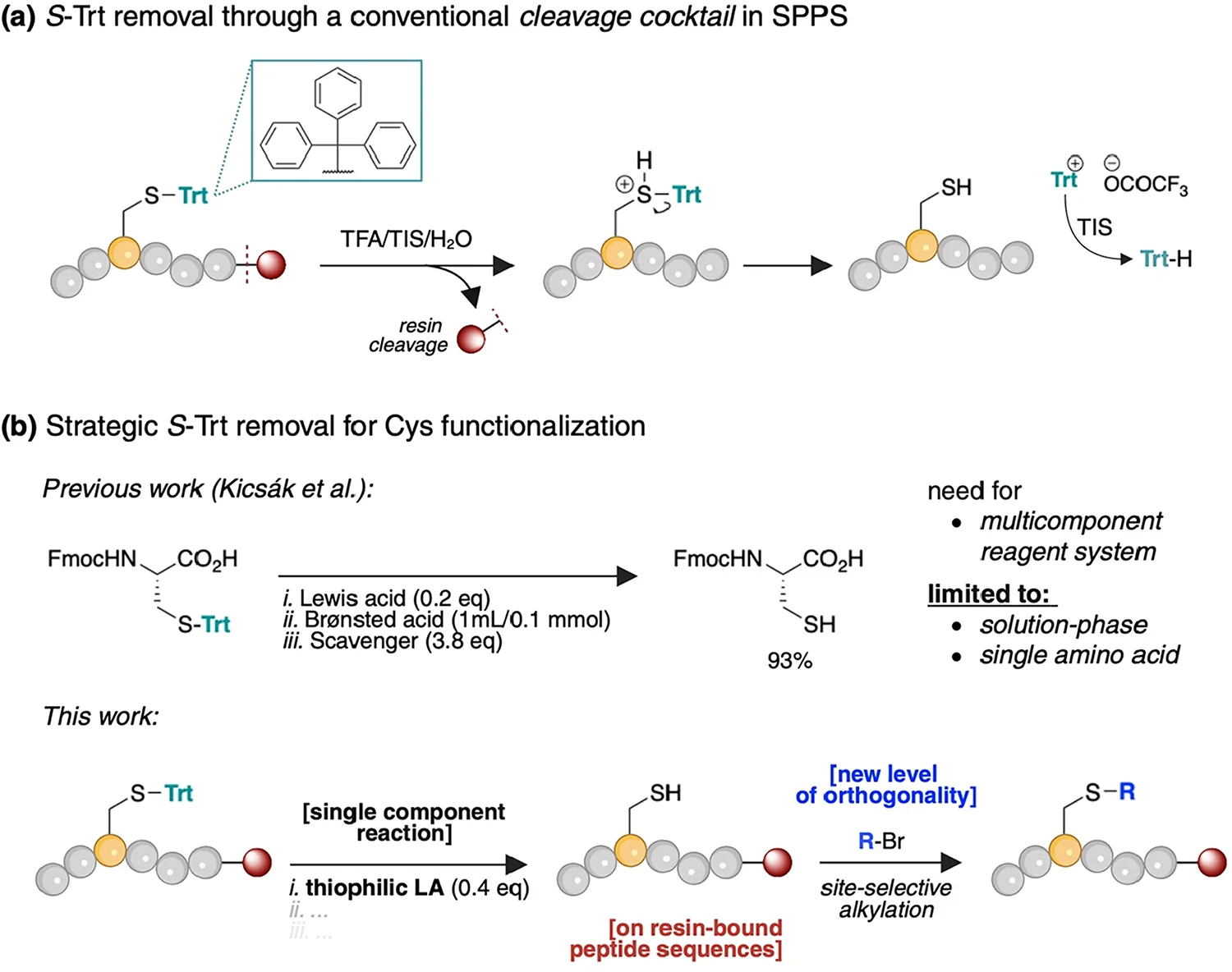
Overview of our study introducing a novel strategy for *S*-Trt removal in SPPS. (a) Conventional acid-mediated approach during resin cleavage. (b) Selective on-resin *S*-detritylation enabling cysteine functionalization.

Therefore, Trt is a straightforward and well-established Cys protecting group used in the Fmoc-based SPPS. Despite its widespread use, however, selective removal of the *S*-Trt group from cysteine on solid support has received surprisingly limited attention and, to the best of our knowledge, no protocol has been specifically developed for this transformation. This methodological gap is particularly relevant in the context of late-stage peptide diversification, where controlled thiol unmasking could provide direct access to site-selective cysteine functionalization under SPPS conditions. To address this challenge, we investigated a thiophilic transition metal-based strategy for on-resin *S*-Trt removal that could be exploited for the site-selective fine-tuning of Cys-containing peptides (Figure 1b). The resulting synthetic route was evaluated for its compatibility with other protecting groups and its synthetic utility, ultimately enabling a new level of orthogonality in solid-phase synthesis and late-stage cysteine functionalization. In addition, a reaction mechanism was proposed and supported by MS and NMR monitoring of the *S*-detritylation end-products.

## Results and Discussion

### On Solid-Phase *S*-Detritylation Setup

Earlier studies on the removal of Trt as a protecting group for Cys thiols described the use of the aforementioned metal salts, such as AgNO_3_ in the presence of pyridine, or treatment with boiling TFA to achieve complete deprotection.^17,20^ However, these reaction conditions are incompatible with Fmoc-SPPS protocols. Kicsák *et al.* relied on a solution-phase detritylation protocol consisting of three-component reagent systems: (i) a Lewis acid (LA), (ii) a Brønsted acid, and (iii) a quenching agent (*e*.*g*., TIS), acting in concert to promote efficient deprotection.^19^ Aiming to re-evaluate the use of LAs in SPPS, we started from this study to investigate alternative and compatible Trt removal protocols. While numerous LAs have been employed for the removal of *O*-, *N*- and *S*-trityl protecting groups,^25–31^ we focused on transition metals with pronounced thiophilicity.^32^ It is known that “late” transition metals (right-side d-block elements) display strong affinity for sulfur while showing comparatively lower reactivity toward more electronegative heteroatoms such as oxygen.^33,34^ This trend correlates with the thiophilicity scale reported by Kepp,^32^ in which Cu­(I), Cu­(II), Zn­(II), and Ag­(I) are ranked among the most thiophilic d-block metals. From this set, we selected commercially available Cu­(I), Cu­(II), Zn­(II), and Ag­(I) salts for our investigation. In addition to their high thiophilicity, these metals were prioritized because they combine strong sulfur affinity with a comparatively lower toxicity. These considerations make them particularly attractive for application in SPPS, where compatibility with resin-bound substrates and downstream handling are critical. For the Brønsted acid component, we examined trifluoroacetic acid (TFA), which is commonly used in orthogonal deprotection steps during Fmoc-based SPPS.^35,36^ Therefore, to establish our protocol, we employed a model resin-bound tetrapeptide, Ac-Cys­(Trt)-Lys­(Boc)-Phe-Ala, assembled on Rink amide resin (**1**, Figure 2). The peptide was synthesized using the Fmoc-based ultrasonic-assisted SPPS (US-SPPS), a strategy previously developed by some of us.^37^ For each *S-*detritylation experiment, an aliquot of resin-bound peptide (50 μmol) was treated with the three-component inspiring *cocktail*, consisting of (i) a thiophilic LA (Cu­(I), Cu­(II), Zn­(II), or Ag­(I) salts; 0.4 equiv), (ii) a protic acid (1% TFA), and (iii) a scavenger (TIS, 7.6 equiv), in DCM at rt for 16 h. Acetic acid (AcOH) and 2,2,2-trifluoroethanol (TFE) were also briefly evaluated as alternative Brønsted acids, but negligible or no conversion to the desired product was observed under these conditions (data not shown).

**Figure 2:**
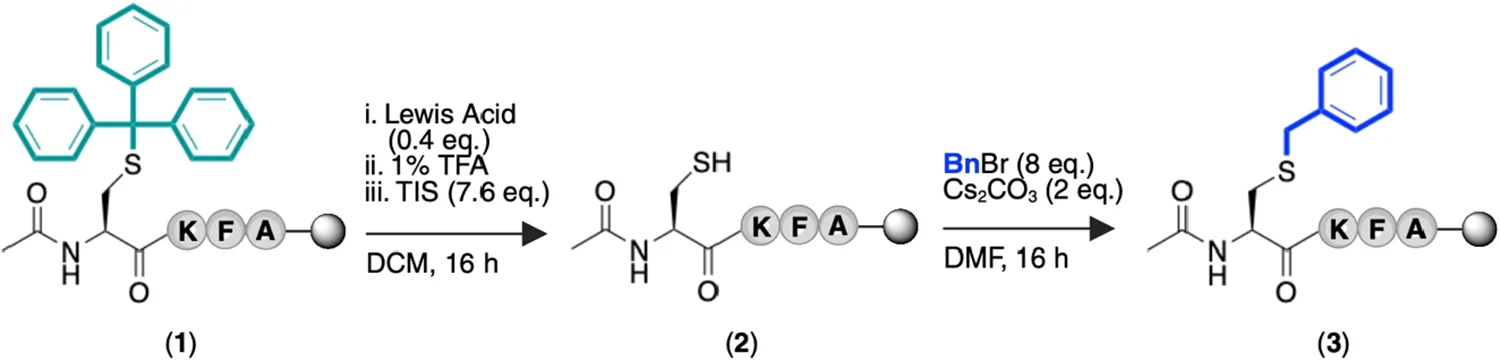
Thiophilic transition metal-mediated *S*-detritylation and subsequent on-resin cysteine benzylation.

To evaluate the efficiency of the *S*-detritylation process, the release of the thiol group in **2** (Figure 2) was initially monitored using Ellman’s test,^38^ a rapid thiol-sensitive colorimetric assay. The resin-bound thiol was subsequently subjected to *S*-benzylation, and the resulting peptide (**3**, Figure 2) was analyzed by HPLC peak integration after TFA cleavage. Unless otherwise stated, all reported % conversion values throughout this work refer to the corresponding *S*-benzylated peptide and therefore provide an indirect measure of the preceding *S*-detritylation step.

In the established solid-phase *S*-detritylation model, conversion rates were analyzed with respect to the combined effects of thiophilic d-block metal salts and the selected Brønsted acid (entries **1–20**, Table 1). By using 1% TFA in DCM as the protic medium, a broader spectrum of salts displayed measurable activity, though efficiencies varied. In general, halogenated salts of Cu, Zn, and Ag (entries **1**, **2**, **7–9**, and **14–16**) afforded only modest conversion, typically below 10%, with the sole exception of CuBr_2_ (35 ± 8%). Among Cu­(II) salts, reactivity was strongly counterion-dependent, with conversion ranging from no detectable *S*-alkylated product to moderate values around 35%. In contrast, Zn­(II) salts were consistently less effective, resulting in only low to modest conversion (<30%). In sharp contrast, silver salts consistently demonstrated the highest reactivity. AgNO_3_ efficiently promoted the *S*-detritylation process, leading to 62 ± 5% conversion to the corresponding *S*-benzylated peptide. Ag_2_SO_4_ and AgOAc also provided substantial conversion (>40%), underscoring the crucial influence of the counterion on reaction outcome. Among the tested salts, Ag­(CF_3_CO_2_) (entry **20**) afforded the highest conversion (80 ± 6%), followed by AgNO_3_ and AgOAc or Ag_2_SO_4_ (∼50%), revealing a clear counterion-dependent trend in efficiency: CF_3_CO_2_^–^ > NO_3_^–^ > AcO^–^/SO_4_^2–^. These results indicated Ag­(I) as the most consistently thiophilic and efficient species under the tested conditions, while underscoring the critical role of the counterion. Weakly coordinating anions such as CF_3_CO_2_^–^ and NO_3_^–^ enhanced the accessibility and reactivity of Ag­(I), whereas more strongly coordinating anions (AcO^–^ and SO_4_^2–^) diminished its effectiveness in promoting *S*-detritylation and resulted in lower overall conversions. Accordingly, the superior performance of AgNO_3_ (entry **18**) and Ag­(CF_3_CO_2_) (entry **20**) identified these silver salts as the most promising candidates for further optimization of the solid-phase *S*-detritylation protocol (Figures S2–S3, Supporting Information).

**Table 1:** Screening of Lewis Acids for on-Resin *S*-Detritylation of the Model Tetrapeptide Cys­(Trt)-Lys­(Boc)-Phe-Ala (**1**) Evaluated Through Conversion to the *S*-Benzylated Product (**3**)

Entry	Lewis Acid^a^	% Conv.^b^
**1**	CuCl_2_	9 ± 9
**2**	CuBr_2_	35 ± 8
**3**	CuSO_4_	33 ± 7
**4**	Cu(NO_3_)_2_	n.d.
**5**	Cu(OAc)_2_	36 ± 7
**6**	Cu(CF_3_CO_2_)_2_	36 ± 7
**7**	ZnCl_2_	4 ± 4
**8**	ZnBr_2_	5 ± 1
**9**	ZnI_2_	11 ± 10
**10**	ZnSO_4_	16 ± 7
**11**	Zn(NO_3_)_2_	3 ± 1
**12**	Zn(OAc)_2_	20 ± 3
**13**	Zn(CF_3_CO_2_)_2_	25 ± 3
**14**	AgCl	11 ± 10
**15**	AgBr	8 ± 8
**16**	AgI	6 ± 5
**17**	Ag_2_SO_4_	43 ± 5
**18**	AgNO_3_	62 ± 5
**19**	AgOAc	46 ± 5
**20**	Ag(CF_3_CO_2_)	80 ± 6

a Lewis acid used in 0.4 equiv.

b Conversion determined by HPLC peak integration after *S*-benzylation step performed with BnBr (8 equiv) and Cs_2_CO_3_ (4 equiv) in DMF for 16 h. n.d.= not detected.

### Optimization of Reaction Conditions

Following preliminary screening, AgNO_3_ and Ag­(CF_3_CO_2_) emerged as the most promising Lewis acids, as both afforded conversion values exceeding 60% under the initial conditions. Therefore, this threshold was adopted as a practical cutoff to select candidates for further optimization, ensuring that only salts with consistently high efficiencies were advanced. With these two Ag­(I) salts, a new series of experiments was designed to develop a faster and more efficient protocol by systematically varying both the stoichiometry of the metal salt and the reaction time (Table 2; and Figures S4–S21, Supporting Information). In the initial procedure, detritylation was carried out for 16 h using 0.4 equiv of the metal salt. Albeit effective, such prolonged conditions were less attractive for practical SPPS workflows, where shorter cycle times and minimized reagent consumption were strongly preferred. To address this, lower (0.2 equiv) and higher (0.8 equiv) salt loadings were explored, in addition to the standard 0.4 equiv, to assess the impact of reagent stoichiometry on conversion. Reaction time was likewise shortened from 16 h to significantly reduced intervals and tested in duplicate cycles of 0.25, 1, and 4 h. These time frames were selected to probe the balance between kinetic efficiency and practical compatibility with SPPS protocols.

**Table 2:**
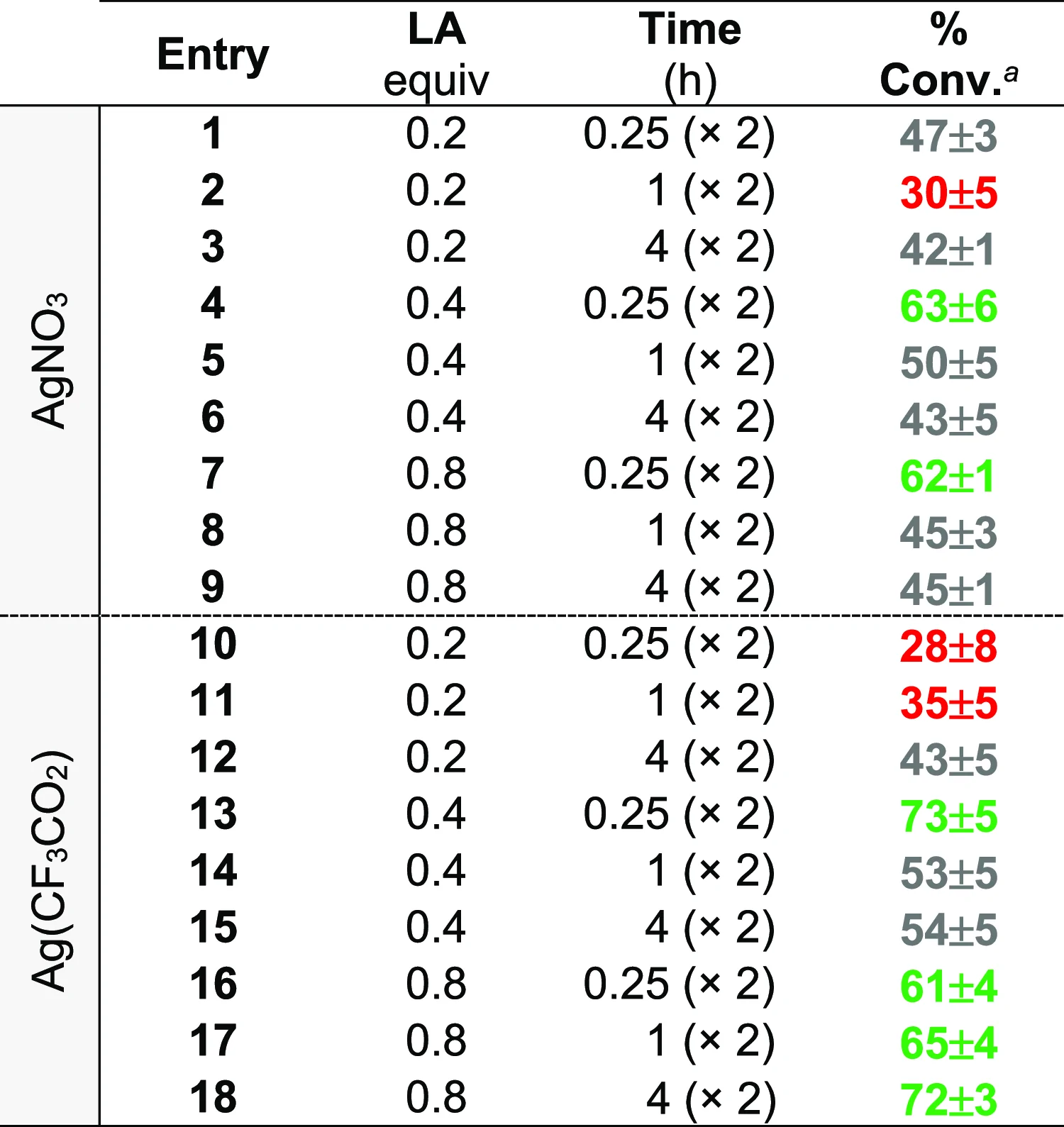
Optimization of Solid-Phase *S*-Detritylation Using Ag­(I) Salts^b^

a Green, conversion rate >60%; dark gray, 40%.

b Effect of reagent stoichiometry and reaction time.

The results summarized in Table 2 revealed distinct differences between the two Ag­(I) salts. AgNO_3_ afforded moderate to good conversions (30–65%) under short reaction times (0.25 h × 2) (entries **1**, **4**, and **7**), while longer treatments generally led to a slight decrease in efficiency (≤50%). This suggested that extended exposure to silver nitrate may promote partial silver complexation or side interactions with the resin, ultimately reducing the availability of the active Ag^+^ species. The best result for AgNO_3_ (entry **4**) was obtained using 0.4 equiv (63 ± 6%), although a comparable value was also obtained with 0.8 equiv (62 ± 1%). Considering the marginal difference in performance, the use of 0.4 equiv appeared preferable, as it achieved the same level of conversion with lower reagent consumption. On the other hand, lower loadings (0.2 equiv) proved less effective (entry **1**: 47 ± 3%). Ag­(CF_3_CO_2_) exhibited overall higher efficiency, consistently affording conversions above 60% under the most favorable conditions. The highest conversion (73 ± 5%) was achieved using 0.4 equiv in two short reaction cycles (0.25 h × 2) (entry **13**), while higher silver loadings (0.8 equiv) offered no significant advantage (entries **16–18**: 61–72%). Accordingly, short and duplicate cycles (0.25 h × 2) combined with moderate silver loadings (0.4 equiv) (entries **4** and **13**) offered the best compromise between efficiency (>65% conversions) and reagent economy.

### Study of the Reaction Mechanism

To gain mechanistic insight into the optimized *S*-detritylation protocol, a targeted set of control experiments was conducted under the best-performing conditions identified so far [0.4 equiv of AgNO_3_ (Table 2, entry **4**) or Ag­(CF_3_CO_2_) (Table 2, entry **13**), 1% TFA in DCM, 7.6 equiv TIS, two cycles of 0.25 h]. These experiments were designed to clarify the individual and combined contributions of the Brønsted acid (*i*.*e*., TFA) and the scavenger (*i*.*e*., TIS) as well as to assess whether the protocol still proceeds through a cooperative mode. Given that TFA may facilitate the formation of a trityl cation (Trt^+^) intermediate, whereas TIS is expected to quench reactive carbocationic species and suppress possible *S*-Trt bond reformation, each component was systematically omitted from the reaction mixture. On this basis, three control conditions were examined for each silver salt: (i) absence of TFA, (ii) absence of TIS, and (iii) absence of both additives (Table 3 and Figures S22–S30, Supporting Information). As a preliminary step, and to clarify the role of TFA in the reaction system, a control experiment was performed to determine whether 1% TFA alone could promote *S*-detritylation. Treatment of the resin-bound peptide with 1% TFA resulted in less than 5% conversion (entry **1**), demonstrating that the Brønsted acid alone is unable to efficiently promote the transformation.

**Table 3:** Effects of Reaction Additives on the Efficiency of the *S*-Detritylation Using Selected Ag­(I) Salts (0.4 equiv, 0.25 h × 2)

		Reaction mixture components		
	**Entry**	**1% TFA**	**TIS**	**% Conv.**
	**1** ^a^	yes	no	<5
AgNO_3_	**2**	yes	no	64 ± 4
	**3**	no	yes	43 ± 8
	**4**	no	no	64 ± 1
Ag(CF_3_CO_2_)	**5**	yes	no	91 ± 3
	**6**	no	yes	53 ± 6
	**7**	no	no	97 ± 1

a Control experiment performed in the absence of Lewis acid.

For AgNO_3_, comparable conversions (64 ± 4% and 64 ± 1%) were obtained in the absence of TIS, regardless of whether TFA was present (entries **2** and **4**). These values were consistent with those previously observed under optimized conditions (Table 2, entry **4**: 63 ± 6%conv) (Figure 3). In contrast, omission of TFA while retaining TIS (entry **3**) led to a significant decrease in conversion (43 ± 8%), likely due to a solvation effect of TFA on AgNO_3_. These findings indicated that TIS alone was unable to sustain efficient *S*-Trt cleavage and may even be detrimental, whereas the Brønsted acid was not strictly required for AgNO_3_-mediated detritylation, as comparable conversions were obtained with its presence and absence when TIS was omitted. A markedly different behavior was observed for Ag­(CF_3_CO_2_). In this case, removal of additives led to a substantial increase in efficiency. In the presence of TFA alone, conversion reached 91 ± 3% (entry **5**), whereas complete omission of both TFA and TIS afforded 97 ± 1% conversion (entry **7**), representing the highest value obtained across all tested conditions and even surpassing that achieved under the previously optimized protocol (Table 2, entry **13**: 73 ± 5%conv), as confirmed by direct comparison (Figure 3). Conversely, inclusion of TIS in the absence of TFA reduced conversion to 53 ± 6% (entry **6**), highlighting the detrimental effect of the silane scavenger. This ∼20% decrease was consistent with the trend observed for AgNO_3_ (Table 3, entries **2** and **4** vs entry **3**). This behavior of TIS in the absence of Brønsted acid suggested that the silane may interfere with the reaction environment, possibly by affecting silver speciation or solubility, thereby attenuating the efficiency of C–S bond cleavage. These findings demonstrated that the requirements for efficient *S*-detritylation differ significantly between the two Ag­(I) salts, confirming the critical role of the counterion. With respect to the assistance of external Brønsted acid or scavenger, Ag­(CF_3_CO_2_) appeared more capable of promoting efficient C–S bond cleavage than AgNO_3_ (Figure 3).

**Figure 3:**
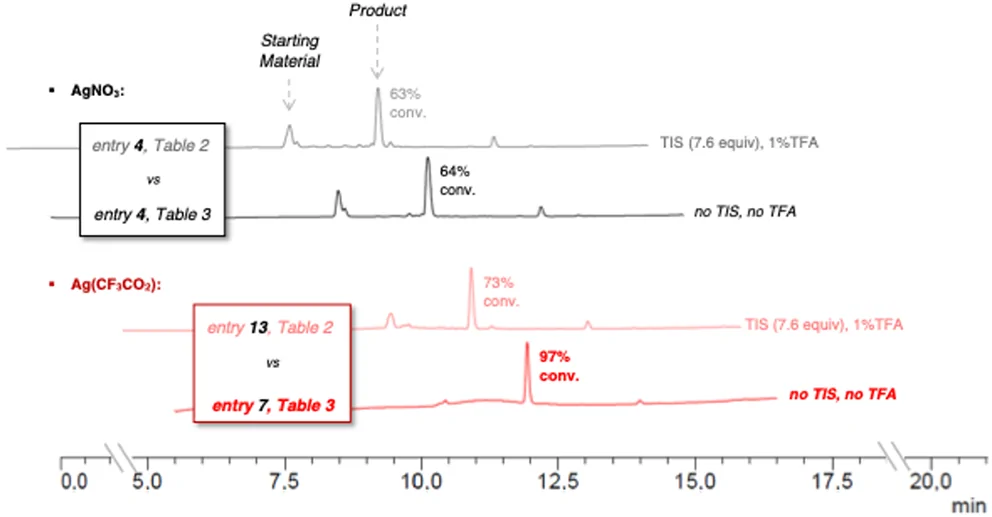
Overlap of representative HPLC chromatograms comparing the effect of additives on Ag­(I)-mediated *S*-detritylation. AgNO_3_ (entries **4**, Table 2 and **4**, Table 3) showed comparable conversions in the presence or absence of TIS (7.6 equiv) and TFA. Ag­(CF_3_CO_2_) (entries **13**, Table 2 and **7**, Table 3) exhibited significantly enhanced conversion in the absence of both additives, reaching up to 97% conversion.

To further elucidate the reaction pathway, the combined filtrates obtained after the two detritylation cycles performed under the most efficient conditions (Table 3, entry **7**) were collected and analyzed. After solvent removal, the residue was first examined by LC-MS (single quadrupole), revealing a single major species corresponding to the triphenylmethyl cation (Trt^+^) (Figure 4A). High-resolution mass spectrometry (HRMS) further detected a signal attributable to Trt^+^ (calcd *m*/*z* 243.1168) as the most abundant ion at *m*/*z* 243.1167. Although this corresponded to Trt^+^, whose high stability is well-known and commonly attributed to resonance stabilization and extensive delocalization of the positive charge over the three phenyl rings,^22^ the nonanhydrous conditions employed in our protocol led to conversion of Trt^+^ into Trt–OH, with the detected ion at *m*/*z* 243.1167 arising from fragmentation of the latter under MS conditions.^39^ These observations were supported by direct comparison of MS and HPLC analyses of the filtrates with those of commercially available Trt–OH (Figures S31–S34, Supporting Information). The residue was subsequently characterized by ^1^H NMR spectroscopy, which confirmed the presence of Trt–OH (Figure 4B). To further support the formation of this species, ^13^C NMR analysis was also performed, revealing no signals over 200 ppm, where carbocationic centers such as that of Trt^+^ could resonate.^40^ Moreover, the spectrum showed signals fully consistent with those of an authentic Trt–OH sample prepared as a reference (Figure 4B). Based on these experimental outcomes, a plausible reaction mechanism was proposed (Figure 4C). Initially, Ag­(I) is proposed to coordinate to the sulfur atom of the Trt-protected cysteine residue, in line with its well-established thiophilicity. Such coordination is expected to decrease the electron density at sulfur and weaken the adjacent C–S bond, thereby facilitating heterolytic cleavage and generating a silver thiolate species on resin together with a trityl cation in solution. The formation of Trt–OH can be rationalized by trapping of the Trt^+^ by adventitious water under nonanhydrous conditions, as supported by the HRMS and NMR data. Ultimately, this pathway would account for the release of the cysteine thiol through *S*-Trt removal, while the peptide remains resin-bound and the trityl-derived species Trt–OH is removed during the washing steps.

**Figure 4:**
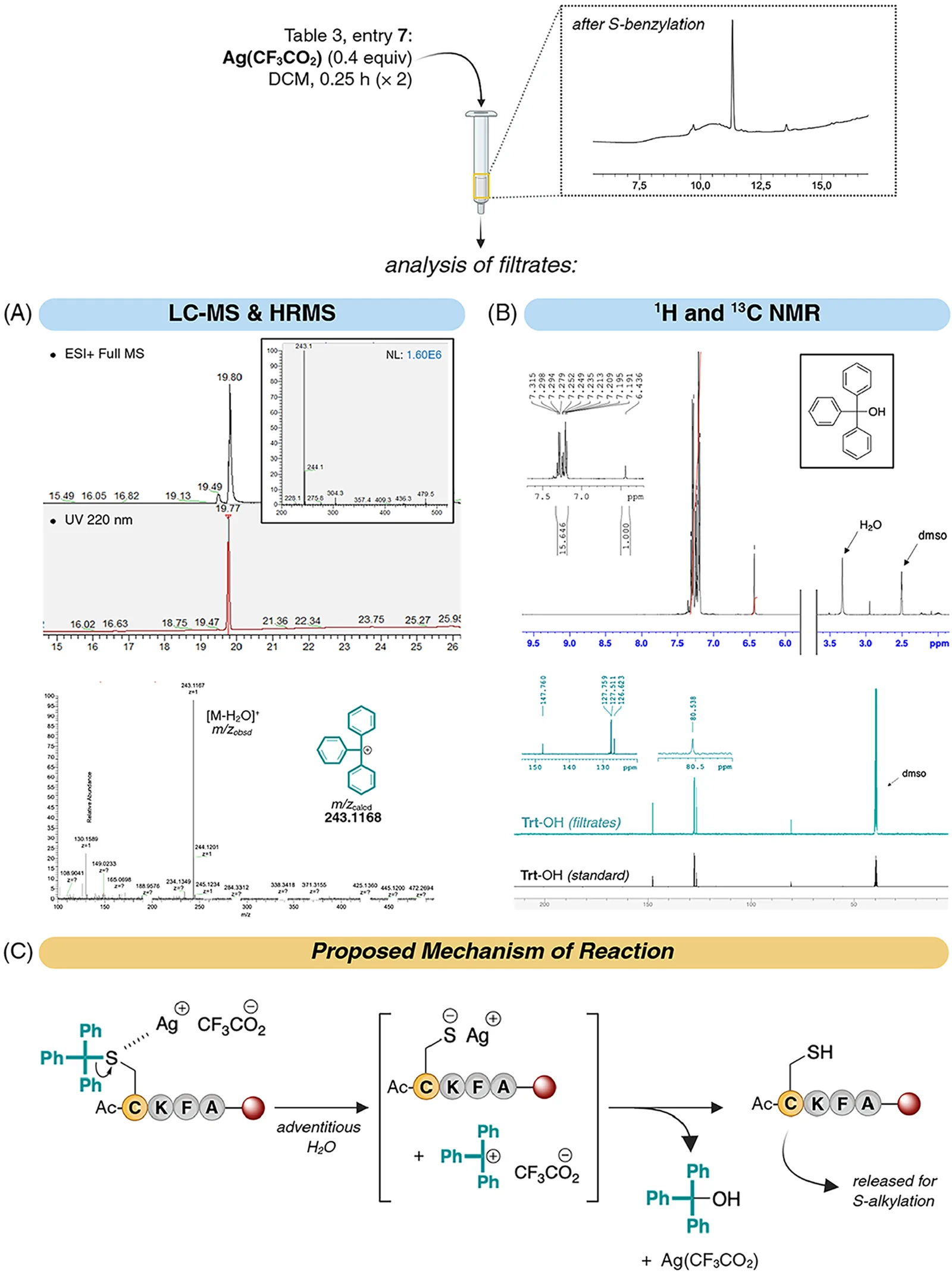
Mechanistic investigation of the Ag­(CF_3_CO_2_)-mediated *S*-detritylation on solid support. The combined filtrates were analyzed through (A) LC-MS and HRMS analysis, showing a major species corresponding to the triphenylmethyl cation (Trt^+^), and (B) ^1^H and ^13^C NMR analyses, confirming the presence of triphenylmethanol (Trt–OH). (C) Proposed mechanism of the Ag­(CF_3_CO_2_)-mediated *S*-detritylation on solid support.

In addition to the proposed reaction pathway, the extent of *S*-detritylation was quantified independently. UV spectroscopy experiments were carried out to determine the amount of Trt–OH that was formed during the reaction. For accurate quantification, the peptide loading on the solid support was first determined. The tetrapeptide sequence Fmoc-Cys­(Trt)-Lys­(Boc)-Phe-Ala was assembled on resin as described above. After completion of the synthesis, the Fmoc group was removed and quantified according to a previously established UV-based protocol,^37^ allowing determination of the peptide loading on the resin, which was taken as the reference value (0.2082 mmol). The *N*-terminus was then acetylated, and our *S*-detritylation standard protocol [Ag­(CF_3_CO_2_) (0.4 equiv) in DCM, two cycles of 15 min] was applied. The reaction filtrates collected after each treatment were combined and analyzed by UV spectroscopy (Figure 5). The presence of the trityl-derived species Trt–OH in solution was confirmed by the absorption band at 227 nm. Trt–OH was used as an external reference, and quantification was performed using a calibration curve constructed from commercially available Trt–OH. Based on this analysis, the measured absorbance corresponded to a trityl concentration of 43.1 mM in the collected solution (4 mL), equivalent to 0.172 mmol of trityl species. This value corresponds to ∼83% of the trityl groups removed from the resin-bound peptide and recovered in the filtrates. The result is in good agreement with the conversion previously determined by HPLC analysis of the corresponding *S*-benzylated peptide. Although the two analytical methods quantify different chemical entities (released trityl-derived species by UV analysis and the corresponding *S*-benzylated peptide by HPLC), the close correspondence between the values obtained provides independent support for the efficiency of the proposed *S*-detritylation protocol.

**Figure 5:**
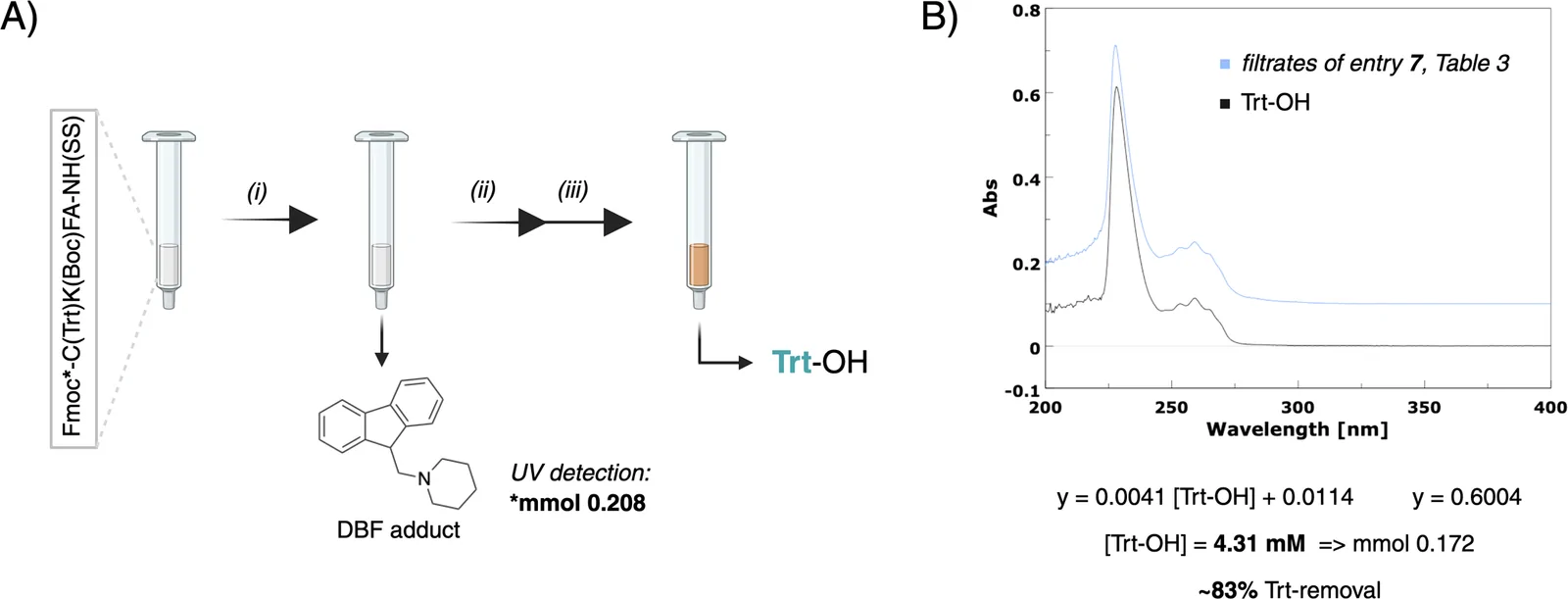
UV-based quantification of trityl removal during Ag­(CF_3_CO_2_)-mediated *S*-detritylation. (A) Schematic representation of the experimental workflow: (i) Fmoc removal; (ii) *N*-terminal acetylation; (iii) *S*-detritylation. (B) UV spectra of the collected filtrates compared with a Trt–OH reference, showing a characteristic absorption maximum at ∼227 nm.

### Compatibility with Ultrasonication, Green Solvents, and Other Protecting Groups

Having established the effectiveness of the single-component protocol based on Ag­(CF_3_CO_2_) (0.4 equiv) for *S*-detritylation on solid-phase, we next explored the compatibility of the reaction with an alternative enabling technology and more sustainable reaction conditions. Ultrasonication (US) was selected as an energy-efficient technology capable of enhancing mass transfer and accelerating heterogeneous transformations. The use of ultrasound in SPPS has been demonstrated to promote efficient mixing and improve the rate of on-resin reactions while reducing both reaction times and solvent consumption.^41–43^ The application of ultrasound to the present system was further motivated by our recent studies showing that US irradiation effectively assisted reactions performed on solid supports, leading to improved efficiency in solid-phase protocols.^37,44,45^ Hence, a more detailed kinetic investigation was performed by varying the reaction time while maintaining standard conditions. Different reaction times were examined (2 × 30, 2 × 15, 2 × 8, 2 × 4, and 2 × 2 min) under ultrasonication (entries **1–5**, Figure 6A). Ultrasonication proved fully compatible with the Ag­(CF_3_CO_2_)-mediated *S*-detritylation protocol, achieving high conversions over a broad range of reaction times (Figures S39–S44, Supporting Information). Conversions of ∼80% were observed for 2 × 30 min (entry **1**) and 2 × 15 min (entry **2**) treatments, while shorter irradiation times [2 × 8 (entry **3**) and 2 × 4 min (entry **4**)] still afforded 68–73% conversion, remaining close to 60% even after only 2 × 2 min cycles (entry **5**). These results suggested that the ultrasound-assisted process effectively supported *S*-Trt deprotection and ensured efficient mixing within the heterogeneous system, although no substantial improvement over conventional mechanical agitation was observed. Reducing the Ag­(CF_3_CO_2_) loading from 0.4 to 0.2 equiv resulted in a marked decrease in conversion (41 ± 3%, Figure S39, Supporting Information), confirming that silver loading plays a critical role in achieving efficient *S*-detritylation. Interestingly, this observation is consistent with the trend previously observed during optimization studies reported in Table 2, where lower Ag­(CF_3_CO_2_) loadings also resulted in reduced conversion despite the presence of TIS and 1% TFA. Therefore, although the reaction conditions differ from those employed in the present single-component protocol, both sets of experiments indicate that silver loading is a key parameter governing *S*-detritylation efficiency.

**Figure 6:**
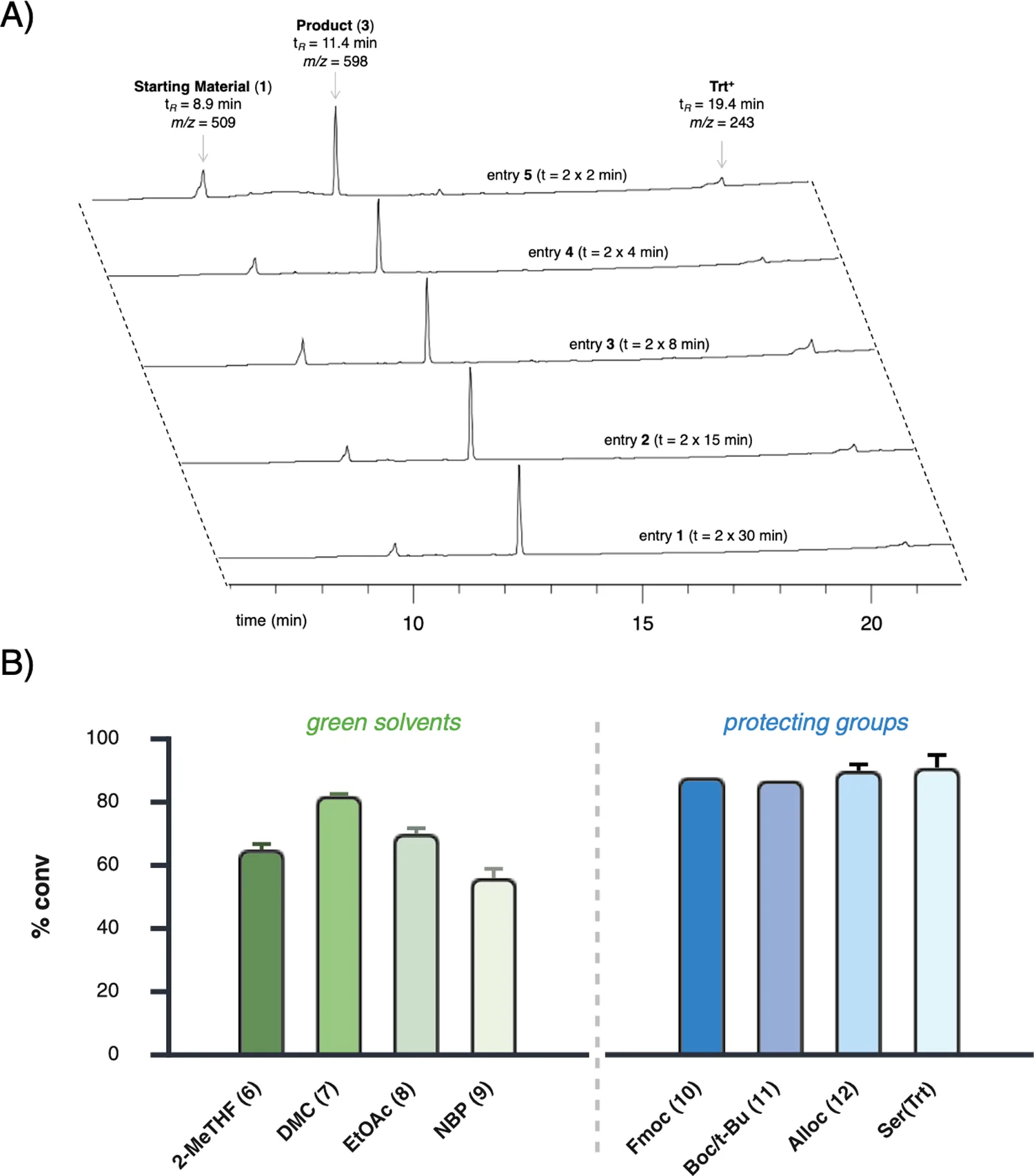
(A) Representative HPLC chromatograms showing the effect of reaction time under ultrasonication. (B) *S*-detritylation efficiency expressed as conversion to the *S*-benzylated product under different conditions. Left: performance in greener solvents. Right: compatibility with commonly employed protecting groups in SPPS.

We next evaluated the compatibility of the protocol with greener solvent systems and commonly employed protecting groups used in SPPS (Figure 6B). Although silver salts could not be considered intrinsically green reagents, their use in limited quantities could still be regarded as acceptable when the environmental impact of the solvent was minimized. Therefore, a series of alternative solvents was investigated for the Ag­(CF_3_CO_2_)-promoted *S*-detritylation, with the aim of identifying suitable replacements for DCM.^46,47^ In particular, several greener and less hazardous solvents were examined, including 2-methyltetrahydrofuran (2-MeTHF) (entry **6**), dimethyl carbonate (DMC) (entry **7**), ethyl acetate (EtOAc) (entry **8**), and *N*-butylpyrrolidinone (NBP) (entry **9**). The results revealed a clear dependence of reaction efficiency on the solvent employed, likely due to differences in polarity and solvation properties influencing Ag­(I) reactivity. Among the tested media, DMC (entry **7**) provided the highest conversion (82 ± 1%), affording efficiencies comparable to those obtained in DCM. 2-MeTHF (entry **6**) and EtOAc (entry **8**) also showed good performance, leading to conversions of 65 ± 3% and 70 ± 3%, respectively, indicating that the *S*-detritylation process tolerated moderately polar solvents. In contrast, NBP (entry **9**) resulted in relatively low conversion (56 ± 5%). Based on these results, our proposed single-component protocol can be effectively implemented under DCM-free conditions, with DMC emerging as a promising green alternative while maintaining high efficiency.

In a further step, the compatibility of the protocol with commonly employed protecting groups in Fmoc-based SPPS was investigated. Up to this point, *S*-detritylation had been performed after *N*-terminal acetylation, thereby avoiding potential compatibility issues with the Fmoc protecting group. To address this aspect, *S*-trityl deprotection and subsequent *S*-alkylation were evaluated on a resin-bound peptide still bearing an *N*-terminal Fmoc group. The tetrapeptide Fmoc-Cys­(Trt)-Lys­(Boc)-Phe-Ala (entry **10**) was thus subjected to Ag­(CF_3_CO_2_)-mediated detritylation in DCM. HPLC and MS analyses of the cleaved product revealed no detectable loss of the Fmoc group, with the Fmoc-protected peptide present at >97% (Figure S49, Supporting Information), demonstrating the feasibility of performing *S*-Trt deprotection and functionalization directly during peptide chain elongation.

Considering that the Trt group is often used in combination with other acid-labile protecting groups, the heptapeptide Ac-Asp­(O*t*Bu)-Gly-Cys­(Trt)-Lys­(Boc)-Phe-Ser­(*t*-Bu)-Ala (entry **11**) was designed as a model sequence to further evaluate the selectivity of the protocol. To probe the stability of *t*-Bu-based protecting groups, an additional capping step with acetic anhydride and DIPEA was performed. LC-MS analysis of the crude peptide after cleavage showed formation of the *S*-benzylated product (conversion 74 ± 3%, crude purity 65%), while the *t*-Bu-based protecting groups remained intact during the detritylation step. As expected, their removal occurred only upon the final acidolytic cleavage, without detection of multiple benzylated or acetylated byproducts (Figure S50, Supporting Information). To rationalize the lower crude purity observed for sequences bearing *tert*-butyl-based protecting groups compared to the other systems investigated, LC-MS analysis was performed. This revealed, in addition to a small but non-negligible amount of the free thiol peptide, attributable to incomplete Trt removal, the presence of two sequence-dependent byproducts formed during cleavage. These species were assigned as an aspartimide derivative and an *S-tert*-butyl peptide, the latter likely arising from reported *tert*-butyl migration from a neighboring lysine residue (Figure S51, Supporting Information).^48^

To further validate the orthogonality of the method, additional model sequences were examined. The resin-bound peptide Ac-Cys­(Trt)-Lys­(Alloc)-Phe-Ala (entry **12**), bearing an Alloc protecting group, was subjected to the *S*-detritylation and *S*-alkylation protocols. LC-MS analysis confirmed formation of the desired *S*-benzylated product without detectable loss of the Alloc side-chain protecting group (90 ± 2%), demonstrating the compatibility of the method with this functionality (Figure S52, Supporting Information). To further investigate the chemoselectivity of the method and its thiophilic character, a model peptide bearing a Ser­(Trt) residue, Ac-Cys­(Trt)-Ser­(Trt)-Phe-Ala (entry **13**), was examined. LC-MS analysis of the crude product after cleavage revealed formation of the sole *S*-benzylated cysteine derivative (92 ± 2%), while the Trt protecting group on the serine side chain remained intact (Figure S53, Supporting Information). The absence of *O*-detritylation was further confirmed by a subsequent acetylation step using acetic anhydride and DMAP (16 h, rt). This result clearly indicated that Ag­(I)-mediated detritylation selectively targeted sulfur over oxygen, highlighting the strong thiophilic character of the Lewis acid employed.

### Synthetic Utility

The results obtained thus far prompted further exploration of the synthetic potential of the developed protocol beyond benzylation, which had initially been employed as a model reaction to assess *S*-detritylation efficiency. In particular, its suitability for the late-stage functionalization of cysteine residues on solid support through *S*-alkylation with structurally diverse alkyl bromides was investigated (Figures S54–S59, Supporting Information). Following *in situ S*-detritylation, the released thiol was directly intercepted by a range of alkyl bromides. As shown in Figure 7, the methodology proved compatible with a variety of substituents, including propargyl (entry **1**), allyl (entry **2**) and geranyl (entry **3**) bromides, allowing the introduction of unsaturated and functionalized aliphatic chains. In these cases, moderate to good conversions (55–80%) were observed, indicating efficient thiol trapping under the optimized conditions. Notably, the protocol also enabled the incorporation of substituents bearing bulky and aromatic moieties, such as 2-naphthylmethyl (entry **4**) and diphenylmethyl (entry **5**) groups, albeit gaining more variable outcomes. In particular, a high conversion was achieved for entry **4** (95 ± 2%), whereas a significantly lower conversion was observed for entry **5** (20 ± 2%), likely reflecting steric hindrance effects during the alkylation step. Finally, the introduction of heteroaromatic moieties, exemplified by ethylindole (entry **6**), was also feasible, affording moderate conversion (47 ± 2%). These results demonstrate that the developed *S*-detritylation protocol can be effectively integrated with subsequent on-resin thiol functionalization reactions. At the same time, the observed variability in conversion highlights that the efficiency of the derivatization step is influenced by the nature of the alkylating agent, suggesting that steric effects primarily impact the *S*-alkylation process rather than the preceding *S*-detritylation step. Finally, our methodology can be readily integrated into standard Fmoc-SPPS workflows, providing an additional straightforward approach for the late-stage diversification of cysteine-containing peptides.

**Figure 7:**
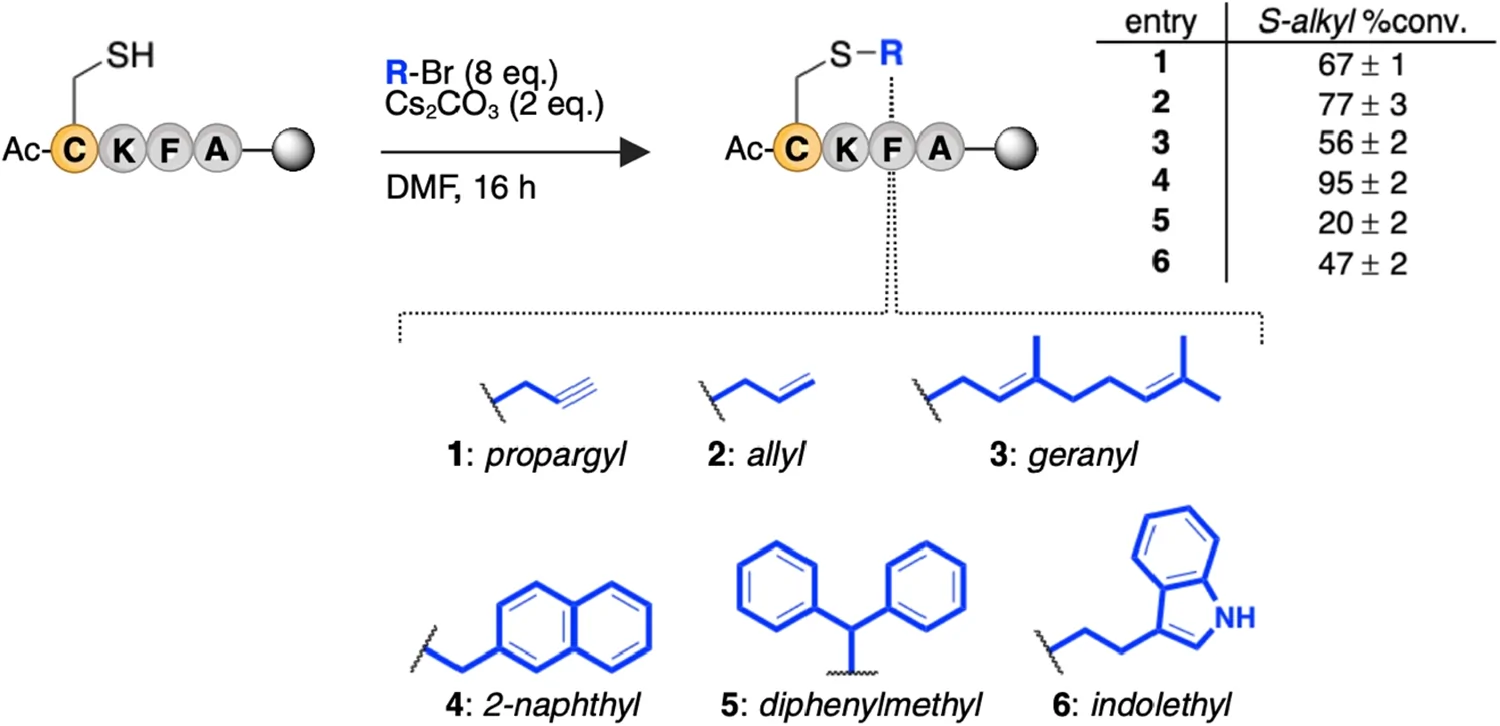
Synthetic utility of the Ag­(CF_3_CO_2_)-mediated *S*-detritylation protocol for on-resin cysteine functionalization.

## Conclusions

In conclusion, we have developed an efficient protocol for the solid-phase *S*-detritylation of cysteine based on the thiophilic properties of silver­(I) salts. Among the Lewis acids investigated, Ag­(CF_3_CO_2_) emerged as the most effective promoter, enabling conversions of up to 97% within short reaction times (30 min total) using substoichiometric loadings. The method operates under mild conditions and exhibits broad compatibility with standard Fmoc-based SPPS, preserving both the *N*-terminal Fmoc group and commonly employed acid-labile side-chain protecting groups, as demonstrated by the selective removal of *S*-Trt over *O*-Trt functionalities. This aspect is particularly relevant considering that Fmoc-Cys­(Trt) remains one of the most widely available and routinely used building blocks in SPPS, despite the lack of efficient protocols for its selective manipulation on solid support. To the best of our knowledge, the present methodology represents the first protocol specifically developed for selective on-resin *S*-detritylation, thereby introducing an additional level of orthogonality into standard Fmoc-SPPS workflows. Mechanistic investigations supported by UV spectroscopy, MS and NMR analyses are consistent with a silver-mediated C–S bond cleavage pathway, leading to the release of trityl-derived species, predominantly detected as Trt–OH under the experimental conditions. In addition, the protocol proved compatible with ultrasound-assisted synthesis and was successfully implemented under DCM-free conditions, with greener solvents such as DMC providing comparable efficiency. Importantly, *in situ* release of the thiol enabled direct on-resin cysteine functionalization, as demonstrated by *S*-alkylation with a range of structurally diverse alkyl substituents. Although the efficiency of the derivatization step depended on the nature of the alkylating agent, with sterically demanding substituents generally affording lower conversions, these limitations were primarily related to the *S*-alkylation process rather than the preceding *S*-detritylation step, which remained consistently efficient under the optimized conditions. The ability to selectively unmask the cysteine thiol from Trt protection on resin further underscores the potential of the method for late-stage diversification of peptide sequences. Overall, this study establishes a valuable foundation for the selective functionalization of cysteine residues in SPPS and opens new opportunities for the development of tailored peptide modifications, including site-selective conjugation and disulfide-based cyclization strategies.

## Methods

### Materials and General Procedures

*N* ^α^-Fmoc-protected amino acids, including Fmoc-Ala-OH, Fmoc-Arg­(Pbf)–OH, Fmoc-Phe-OH, Fmoc-Lys­(Alloc)–OH, Fmoc-Lys­(Boc)–OH, Fmoc-Lys­(Mtt)–OH, Fmoc-Lys­(Trt)–OH, Fmoc-Cys­(Trt)–OH, Fmoc-His­(Trt)–OH, as well as the coupling reagents *N,N,N*′*,N*′*-*tetramethyl-*O*-(1*H*-benzotriazol-1-yl)­uronium hexafluorophosphate (HBTU) and 1-hydroxybenzotriazole (HOBt) were purchased from Iris-Biotech GmbH (Marktredwitz, Germany) or GL Biochem Ltd. (Shanghai, China). Lewis acid salts: copper­(II) chloride (CuCl_2_), copper­(II) bromide (CuBr_2_), copper­(II) sulfate (CuSO_4_), copper­(II) nitrate [Cu­(NO_3_)_2_], copper­(II) acetate [Cu­(OAc)_2_], copper­(II) trifluoroacetate [Cu­(CF_3_CO_2_)_2_], zinc­(II) chloride (ZnCl_2_), zinc­(II) bromide (ZnBr_2_), zinc­(II) iodide (ZnI_2_), zinc­(II) sulfate (ZnSO_4_), zinc­(II) nitrate [Zn­(NO_3_)_2_], zinc­(II) acetate [Zn­(OAc)_2_], zinc­(II) trifluoroacetate [Zn­(CF_3_CO_2_)_2_], silver­(I) chloride (AgCl), silver­(I) bromide (AgBr), silver­(I) iodide (AgI), silver­(I) sulfate (Ag_2_SO_4_), silver­(I) nitrate (AgNO_3_), silver­(I) acetate (AgOAc), silver­(I) trifluoroacetate [Ag­(CF_3_CO_2_)], were purchased from Merck Life Science (Milan, Italy). Trifluoroacetic acid (TFA), triisopropylsilane (TIS), piperidine, *N,N*-diisopropylethylamine (DIEA), cesium carbonate (Cs_2_CO_3_), dimethyl sulfoxide-*d*_6_ (DMSO-*d*_6_), acetic anhydride (Ac_2_O), and 4-(Dimethylamino)­pyridine (DMAP) were purchased from Merck Life Science (Milan, Italy). Fmoc-Rink amide resin (loading of 0.50 mmol/g) was purchased from Rapp Polymere GmbH (Tuebingen, Germany). Anhydrous *N*,*N*-dimethylformamide (DMF) was purchased from Merck Life Science (Milan, Italy). Solvents for peptide synthesis, including DMF, dichloromethane (DCM), dimethyl carbonate (DMC), methanol (MeOH), ethyl acetate (EtOAc), *N*-butyl-2-pyrrolidone (NBP), 2-methyltetrahydrofuran (2-MeTHF), and diethyl ether (Et_2_O), as well as HPLC-grade water and acetonitrile (MeCN), were purchased from commercial sources (Merck Life Science or VWR, Milan, Italy). Plastic syringe tubes (ISOLUTE SPE filtration column) equipped with Teflon filters (ISOLUTE frits, 20 μm porosity polyethylene frits) were acquired by Biotage, Uppsala, Sweden. The ultrasonic bath SONOREX RK 52 H was purchased from BANDELIN electronic (Berlin, Germany).

All chromatograms were recorded on a UHPLC system (Shimadzu Nexera LC-30AD) equipped with a Phenomenex Kinetex C18 column (150 mm × 4.6 mm, 5 μm, 100 Å). Two analytical methods were employed: Method A, a linear gradient of MeCN (0.1% TFA) in water (0.1% TFA) over 10 min; and Method B, a linear gradient of MeCN (0.1% TFA) in water (0.1% TFA) over 15 min, both at a flow rate of 1 mL min^–1^. LC-MS analyses were performed on a Thermo Scientific Ultimate 3000 liquid chromatography system equipped with a Phenomenex Kinetex C18 column (150 mm × 4.6 mm, 5 μm, 100 Å), coupled to a Thermo Scientific LTQ XL linear ion trap mass spectrometer equipped with an electrospray ionization (ESI) source, using Method B at a flow rate of 0.8 mL min^–1^.

### Synthesis of the Model Resin-Bound Tetrapeptide Ac-Cys­(Trt)-Lys­(Boc)-Phe-Ala (1)

The resin-bound model tetrapeptide Ac-Cys­(Trt)-Lys­(Boc)-Phe-Ala (0.05 mmol) was assembled on Fmoc-Rink amide resin (loading of 0.50 mmol/g). The Fmoc-Rink amide resin was placed in a 10 mL plastic syringe reactor equipped with a frit and cap. The resin was swollen in DMF for 20 min under agitation on an automated shaker. Fmoc-deprotection was carried out using 20% piperidine in DMF (5 + 25 min) on an automated shaker. The resin was then washed with DMF (2 mL × 3) and was treated with a solution of the Fmoc-amino acid (3 equiv), HBTU (3 equiv), HOBt (3 equiv), and DIPEA (6 equiv) in DMF, followed by agitation on an automated shaker for 2 h. After the first coupling reaction, the synthesis was continued stepwise using Fmoc-based ultrasound-assisted-solid-phase peptide synthesis (US-SPPS) strategy.^37^ Deprotection and coupling reactions were monitored using the Kaiser test to detect resin-bound primary amines. After peptide elongation, the *N*-terminal Fmoc group was removed, and the *N-*terminus was acetylated using Ac_2_O (2 equiv) and DIEA (4 equiv) in DMF. The reaction mixture was agitated on an automated shaker for 30 min, after which the resin was washed with DMF (2 mL × 3) and DCM (2 mL × 3), and dried under *vacuum*. The identity of the assembled peptide was confirmed by HPLC and MS analysis of a cleaved resin aliquot [5 mg treated with 1 mL of TFA/TIS/H_2_O (95:2.5:2.5, *v*/*v*/*v*)] (Figure S1, Supporting Information).

### Solid-Phase *S-*Detritylation Procedure

The resin-bound model peptides (0.05 mmol) [entries **1–20** (Table 1) and entries **1–18** (Table 2): Ac-Cys­(Trt)-Lys­(Boc)-Phe-Ala (Figures S2–S21, Supporting Information)] were treated with a Lewis acid (0.2, 0.4, or 0.8 equiv), a protic acid (1% TFA), and a scavenger (TIS, 7.6 equiv) in DCM (1 mL). The suspensions were agitated either on an automated shaker (Figures S2–S21, Supporting Information) or under ultrasonic irradiation (Figures S39–S44, Supporting Information) for variable time intervals. The same procedure was performed while systematically omitting the protic acid and/or the scavenger [entries **1–7** (Table 3): Ac-Cys­(Trt)-Lys­(Boc)-Phe-Ala (Figures S22–S30, Supporting Information); entry **10** (Figure 6B): Fmoc-Cys­(Trt)-Lys­(Boc)-Phe-Ala (Figure S49, Supporting Information); entry **11** (Figure 6B): Ac-Asp­(O*t*Bu)-Gly-Cys­(Trt)-Lys­(Boc)-Phe-Ser­(*t*-Bu)-Ala (Figures S50–S51, Supporting Information); entry **12** (Figure 6B): Ac-Cys­(Trt)-Lys­(Alloc)-Phe-Ala (Figure S52, Supporting Information); entry **13** (Figure 6B): Ac-Cys­(Trt)-Ser­(Trt)-Phe-Ala (Figure S53, Supporting Information)]. After treatment, the resins were filtered and washed sequentially with MeOH/DMF (1:1, *v*/*v*, 2 mL × 3), EtOAc (2 mL × 3), and DCM (2 mL × 3). The treatment could be repeated for a second cycle when indicated. The compatibility of the protocol with selected green solvents was also evaluated by replacing DCM with 2-MeTHF, DMC, EtOAc, or NBP (Table S2 and Figures S45–S48, Supporting Information).

### On Solid-Phase *S*-Alkylation Procedure

The resin-bound peptide was treated with alkyl bromide (R-Br) (8 equiv) and Cs_2_CO_3_ (4 equiv) in dry DMF (1.5 mL) and agitated on an automated shaker for 16 h.^5,49^ The resin was then filtered and washed with MeOH/DMF (1:1, *v*/*v*, 2 mL × 3), DMF/H_2_O (8:2, *v*/*v*, 2 mL × 3), EtOAc (2 mL × 3), and DCM (2 mL × 3), and dried under *vacuum*. Successful *S*-alkylation was confirmed by RP-HPLC retention time shift, analyzed by UHPLC and/or LC-MS as above-described (Figures S54–S59, Supporting Information).

### UV Spectroscopic Measurement of Fmoc and Trt Removals

UV absorbance spectra were recorded using the V-750 UV–visible Spectrophotometer (Jasco, USA) equipped with a Peltier temperature controller. Resin loading was determined by Fmoc quantification.^37,50^ Briefly, a known amount of Fmoc-protected resin (12.3 mg) was treated with 20% piperidine in DMF, to remove the Fmoc group, generating the dibenzofulvene-piperidine adduct. After filtration, the resulting solution was collected (6 mL total volume). An aliquot of this solution was then diluted 30-fold with 20% piperidine in DMF prior to UV analysis. A blank solution consisting of 20% piperidine in DMF solution, prepared in the absence of resin was used as reference. An aliquot (500 μL) of the diluted sample was transferred into a quartz cuvette (1 cm optical path length), and UV spectra were recorded in the 200–500 nm range. The absorbance at 301 nm was used for quantification. The concentration of the dibenzofulvene-piperidine adduct in the diluted sample was calculated according to the Beer–Lambert law: *A=* ε *× c × l*, where *A* is the absorbance at 301 nm, ε is the molar extinction coefficient of the dibenzofulvene-piperidine adduct at 301 nm (7.8 mL μmol^–1^ cm^–1^), *c* is the concentration of the adduct in the diluted sample, and *l* is the optical path length (cm). An absorbance value of 0.2430 at 301 nm was recorded for the diluted sample. The concentration in the original deprotection solution was obtained by applying the dilution factor (DF = 30). The total amount of released adduct was then obtained from the corrected concentration and the total volume of the deprotection solution. Finally, the resin loading (Ψ, mmol g^–1^) was determined by dividing the number of moles of released adduct by the mass of the resin. Subsequently, the efficiency of the *S*-detritylation procedure was evaluated by UV analysis of the released Trt-derived species Trt–OH (Figures S36–S38, Supporting Information). After the detritylation reaction, the filtrated solution of entry **7** Table 3, was collected in a graduated cylinder and diluted (DF = 300) with DCM prior to UV analysis. An aliquot of the diluted solution was transferred into a quartz cuvette, and the absorbance was measured at 227.5 nm, corresponding to the released Trt-species. Quantification was performed using a calibration curve prepared from Trt–OH, AgCF_3_CO_2_ (0.8 equiv) in DCM at known concentrations (25, 50, 75, 100, and 150 μM). The concentration of released Trt-derived species in the diluted sample was determined from the measured absorbance using the linear regression equation obtained from the calibration curve: *A =* 0.0041*c +* 0.0114, where *A* is the absorbance at 227.5 nm, *c* is the concentration of Trt-derived species (μM). The calibration curve showed excellent linearity (*R* ^2^ = 0.9993). An absorbance value of 0.6004 at 227.5 nm was recorded for the diluted sample. The concentration in the original filtrate was obtained by applying the DF as 300, and the total amount of released Trt-derived species was calculated from the corrected concentration and the total volume of filtrate volume (4 mL). The *S*-detritylation efficiency was then expressed relative to the amount of cysteine loaded on the resin, as determined by Fmoc quantification.

### NMR Analysis

NMR spectra were recorded on a Bruker instrument (400 MHz for ^1^H and 100 MHz for ^13^C). Chemical shifts (δ) are reported in parts per million (ppm). NMR experiments were recorded at room temperature (298 K) in DMSO-*d*_6_ (2.50/39.52 ppm). Free induction decays (FIDs) were processed into spectral data using TopSpin software. Abbreviations are used to describe peak multiplicities: s (singlet) and m (multiplet). For characterization purposes, the filtrate obtained from the *S*-detritylation procedure (entry **7**, Table 3) was evaporated under reduced pressure to afford a pale-yellow powder. A known amount of the residue was dissolved in DMSO-*d*_6_ and analyzed by NMR spectroscopy (Figure S35, Supporting Information). ^1^H NMR (400 MHz, DMSO-*d*_6_) δ 7.32–7.19 (m, 15H), 6.44 (s, 1H). ^13^C NMR (100 MHz, DMSO-*d*_6_) δ 147.8, 127.8, 127.5, 126.6, 80.5.

## Supplementary Material



## Notes

List of commonly used Fmoc-protected cysteine derivatives for SPPS; analytical data, HPLC chromatograms and MS spectra for model peptides and selected reaction products; LC-MS, HRMS, NMR and UV–vis spectroscopic data supporting the optimization and mechanistic investigation of the Ag­(CF_3_CO_2_)-mediated *S*-detritylation; and additional analytical data supporting protecting-group compatibility studies and on-resin *S*-alkylation (PDF)
