# Supplementary material for: A Thiophilic Metal-Mediated Cysteine S‑Detritylation Strategy: Enhancing Orthogonality in Solid-Phase Peptide Synthesis
Source: ACS Omega. 2026 Sep 25;11(39):59327–38. doi: 10.1021/acsomega.6c05277 (PMC13648059; doi:10.1021/acsomega.6c05277)
Supplement: Supplementary file 1 [file ao6c05277_si_001.pdf]

A Thiophilic Metal-Mediated Cysteine *S*-Detritylation Strategy: Enhancing Orthogonality in Solid-Phase Peptide Synthesis

Salvatore Mottola,[a,§] Rosa Bellavita,[b,§] Ida Boccino,[b] Gennaro Maria Bailo,[b] Roberto Cutolo,[a] Mariateresa Giustiniano,[b] Stefania Galdiero,[b] Paolo Grieco,[b] Salvatore Di Maro,[a] Anna Messere[a,] and Francesco Merlino[b,*]*

[a]DiSTABiF, University of Campania Luigi Vanvitelli, via Vivaldi 43, 81100 Caserta, Italy; [b]Department of Pharmacy, University of Naples Federico II, via Domenico Montesano 49, 80131, Naples, Italy.

[*]Email: anna.messere@unicampania.it; francesco.merlino@unina.it

Table of contents

1. List of commonly used Fmoc-protected Cys for SPPS (Table S1) S2
2. Analytical data of model tetrapeptide **1** (Figure S1) S3
3. Analytical data for entries **18** and **20** of Table 1 (Figures S2 and S3) S4
4. HPLC chromatograms for entries **1-18** of Table 2 (Figures S4-S21) S6
5. HPLC chromatograms for entries **1-7** of Table 3 (Figures S22-S30) S15
6. LC-MS, HRMS spectra of filtrates from S-detritylation reaction and standard Trt-OH, including HPLC chromatograms overlap (Figures S31-S34) S20
7. NMR spectra of filtrates from S-detritylation reaction and standrad Trt-OH (Figure S35) S22
8. UV spectra of filtrates from S-detritylation reaction (Figure S36-S38) S24
9. HPLC chromatograms for $Ag(CF_3CO_2)$ 0.2 equiv and entries **1-5** of Figure 6A (Figures S39-S44) S26
10. HPLC chromatograms for entries **6-9** of Figure 6B (Table S2 and Figures S45-S48) S29
11. Analytical data for entries **10-13** of Figure 6B (Figures S49-S53) S32
12. Analytical data for entries **1-6** of Figure 7 (Figures S54-S59) S36

Table S1. Survey of commercial availability and costs of Fmoc-protected cysteine derivatives bearing acid-labile side-chain protecting groups.

[a]Protecting groups listed are those highly sensitive to acidic conditions

Cys protecting group[a]	Source	Costs[b]
Fmoc-Cys(**Mmt**)	Merck/Sigma-Aldrich	~90 € per gram
Fmoc-Cys(**Xan**)	Merck/Sigma-Aldrich	~500 € per gram
Fmoc-Cys(**Dpm**)	Merck/Sigma-Aldrich	~65 € per 5 g (~13 € per gram)
Fmoc-Cys(**Ddm**)	IRIS-Biotech	~450 € per gram
Fmoc-Cys(**Trt**)	Merck/Sigma-Aldrich	~80 € per 25 g (**~3.2 € per gram**)

and commercially available for use in Fmoc-based SPPS. [b] Costs were estimated based on data from Sigma-Aldrich/Merck and IRIS Biotech websites (accessed April 2026). Mmt: 4-methoxyltrityl; Xan: 9*H*-xanthen-9-yl; Dpm: diphenylmethyl; Ddm: di(4-methoxyphenyl)methyl.

*2. Analytical data for the model tetrapeptide **1***

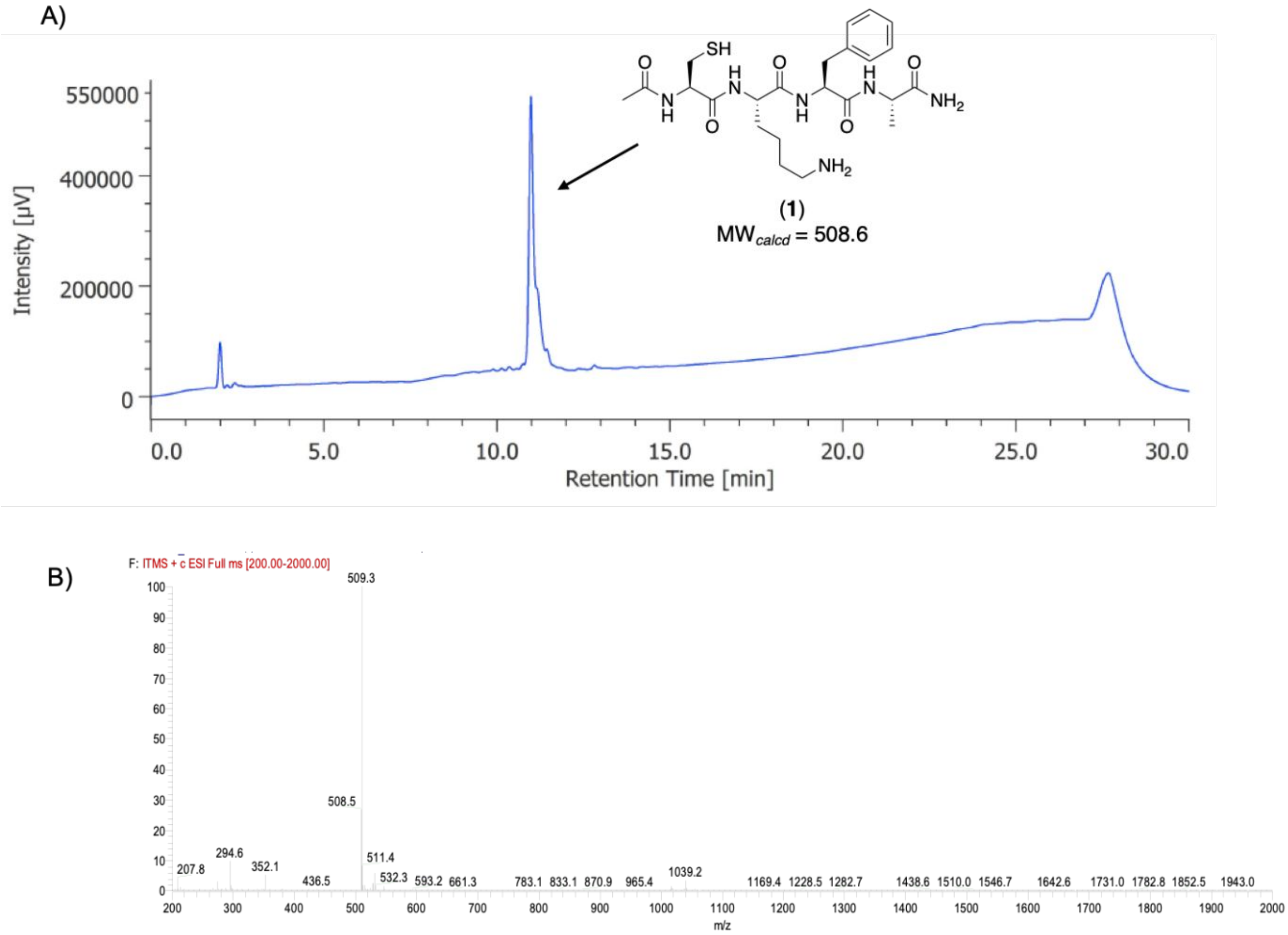

Figure S1. (A) HPLC chromatogram of peptide **1** obtained using Method **A**, at a flow rate of 1 mL min^{-1}, with UV detection at 220 nm. The retention time (t_R) of 10.2 min corresponds to the non-alkylated peptide (**1**, starting material for this study). (B) ESI-MS spectrum acquired for the fraction collected at the t_R corresponding to the single peak highlighted in panel A. Calculated mass for $[M+H]^+$: 509.6; found *m/z* 509.3.

*3. Analytical data for entries **18** and **20** (Table 1)*

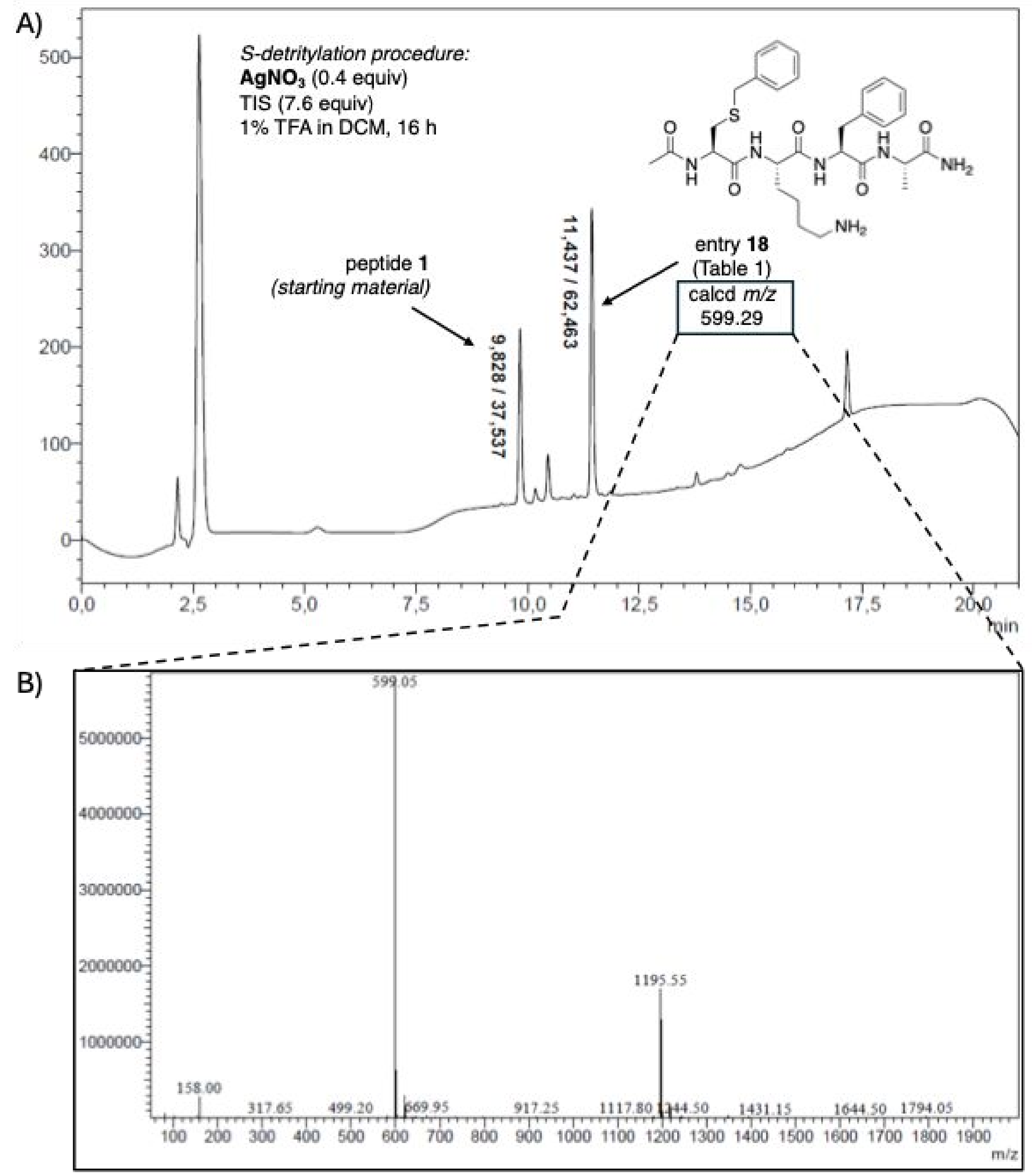

Figure S2. (A) Representative HPLC chromatogram of entry **18** (Table 1) obtained after the *S*-detritylation procedure ($AgNO_3$, 0.4 equiv; TIS, 7.6 equiv; 1% TFA in DCM, 16 h). Analysis was performed using Method **A**, at a flow rate of 1 mL min^{-1}, with UV detection at 220 nm. Retention time (t_R): 11.4 min. (B) Calculated mass for $[M+H]^+$: 599.29; found m/z 599.05; $[M\ dimer + H]^+$ 1195.55.

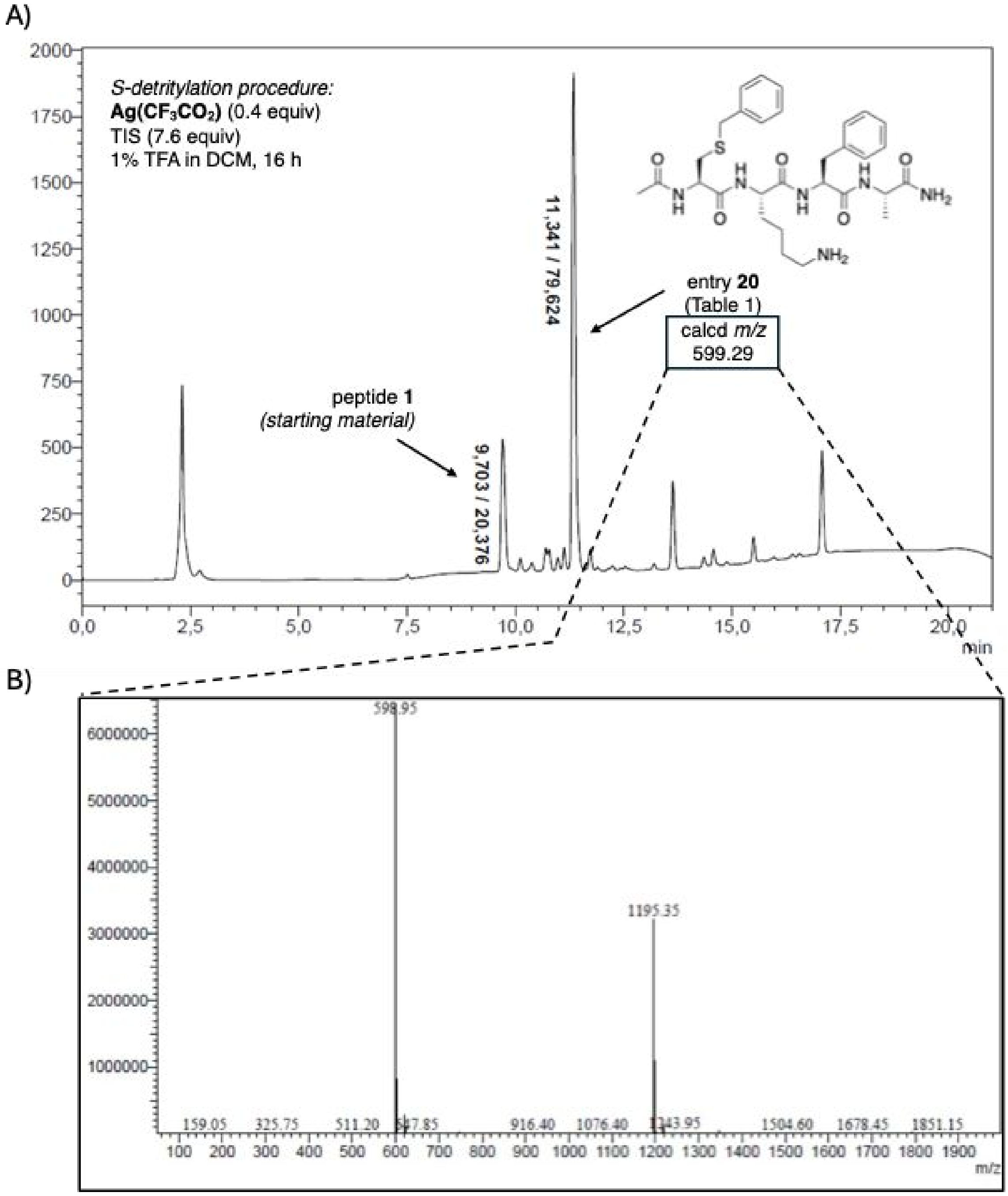

Figure S3. (A) Representative HPLC chromatogram of entry **20** (Table 1) obtained after the *S*-detritylation procedure [Ag(CF_3CO_2), 0.4 equiv; TIS, 7.6 equiv; 1% TFA in DCM, 16 h]. Analysis was performed using Method **A**, at a flow rate of 1 mL min^{-1}, with UV detection at 220 nm. Retention time (t_R): 11.3 min. (B) Calculated mass for $[M+H]^+$: 599.29; found *m/z* 598.95; [dimer +H] $^+$ 1195.35.

*4. HPLC chromatograms for entries **1-18** (Table 2)*

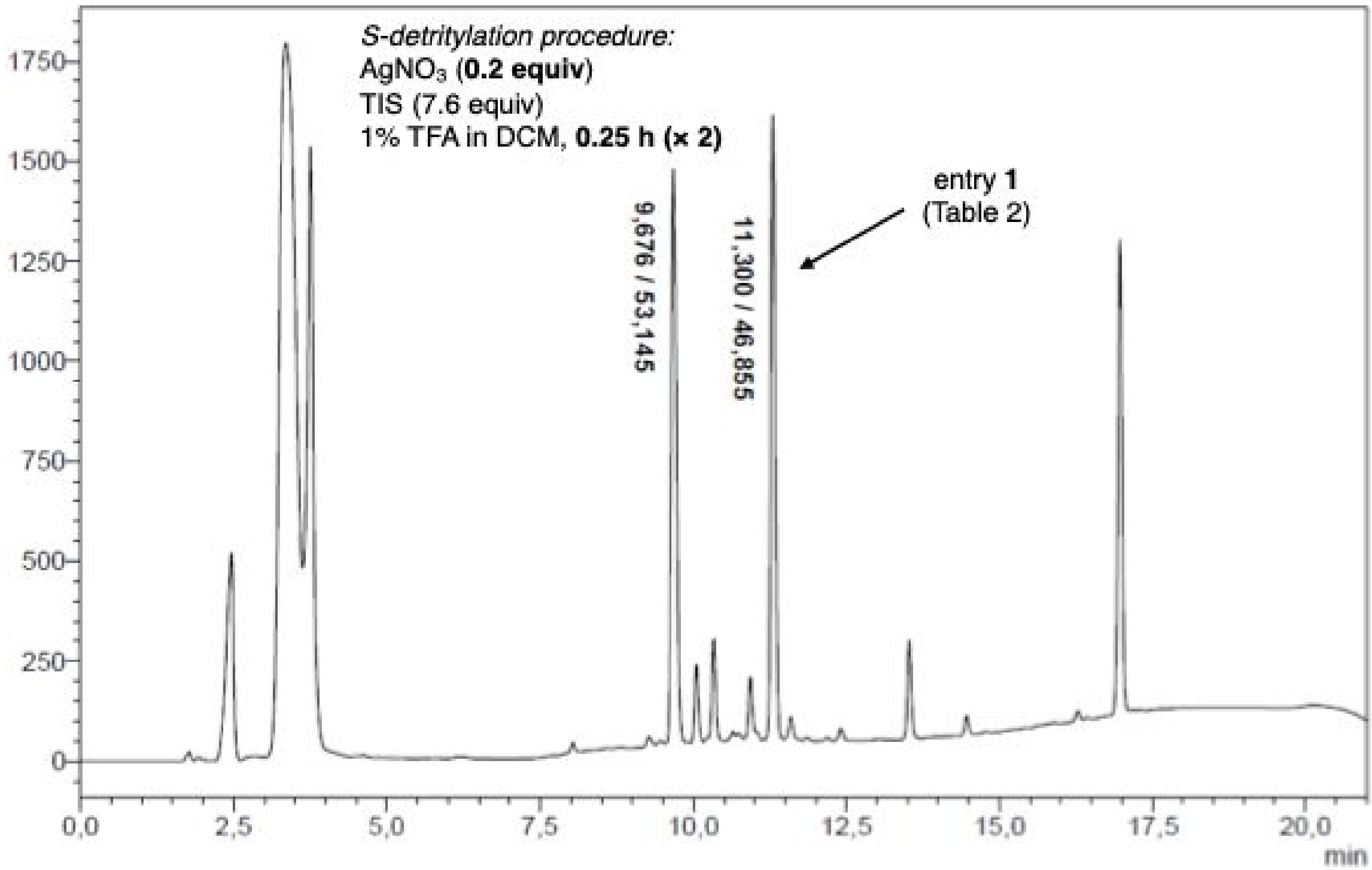

Figure S4. Representative HPLC chromatogram of entry **1** (Table 2) showing 46% conversion to the S-benzylated product, t_R= 11.3 min. Analysis was performed using Method **A**, at a flow rate of 1 mL min^{-1}, with UV detection at 220 nm.

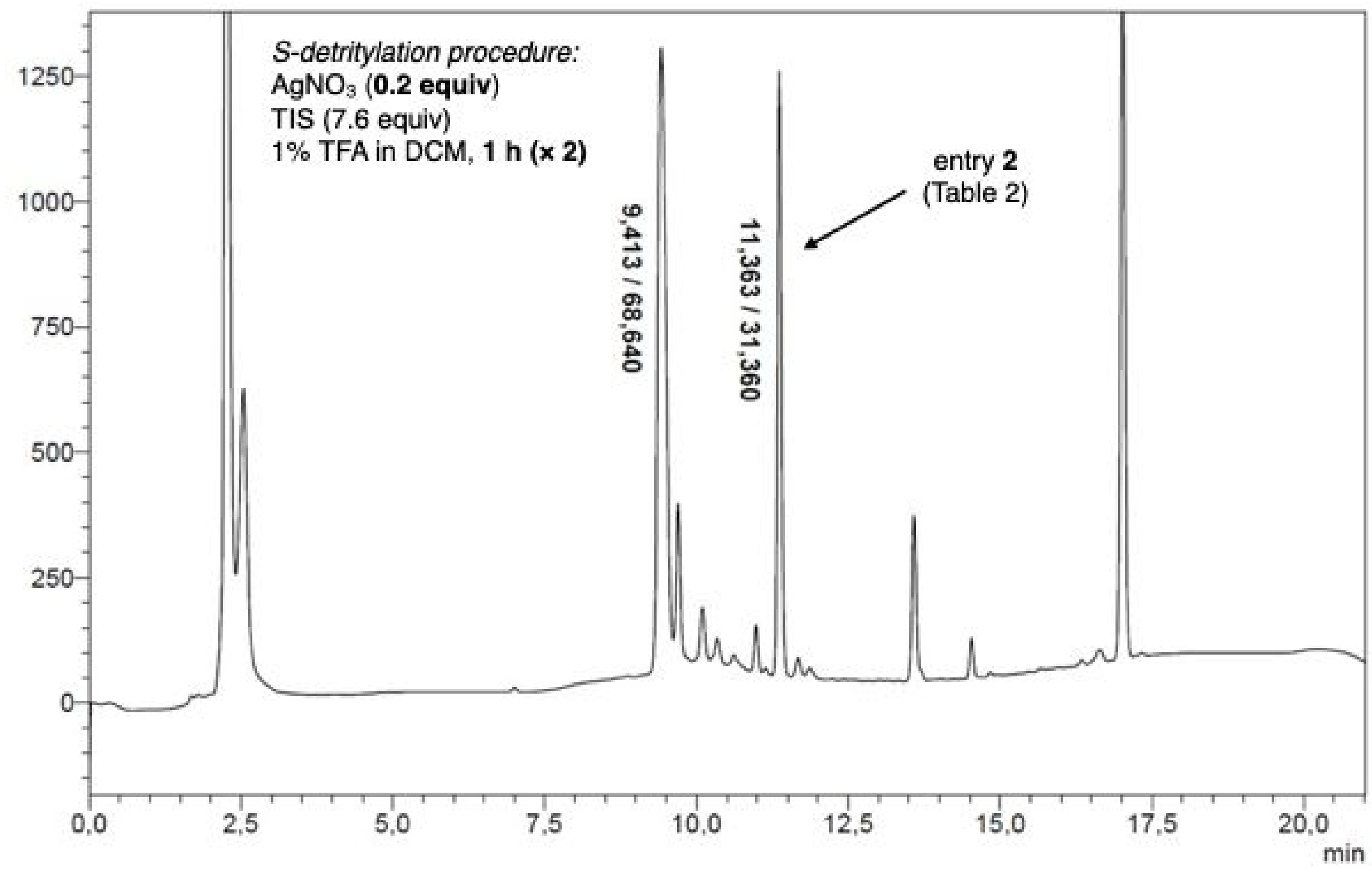

Figure S5. Representative HPLC chromatogram of entry **2** (Table 2) showing 31% conversion to the S-benzylated product, t_R= 11.3 min. Analysis was performed using Method **A**, at a flow rate of 1 mL min^{-1}, with UV detection at 220 nm.

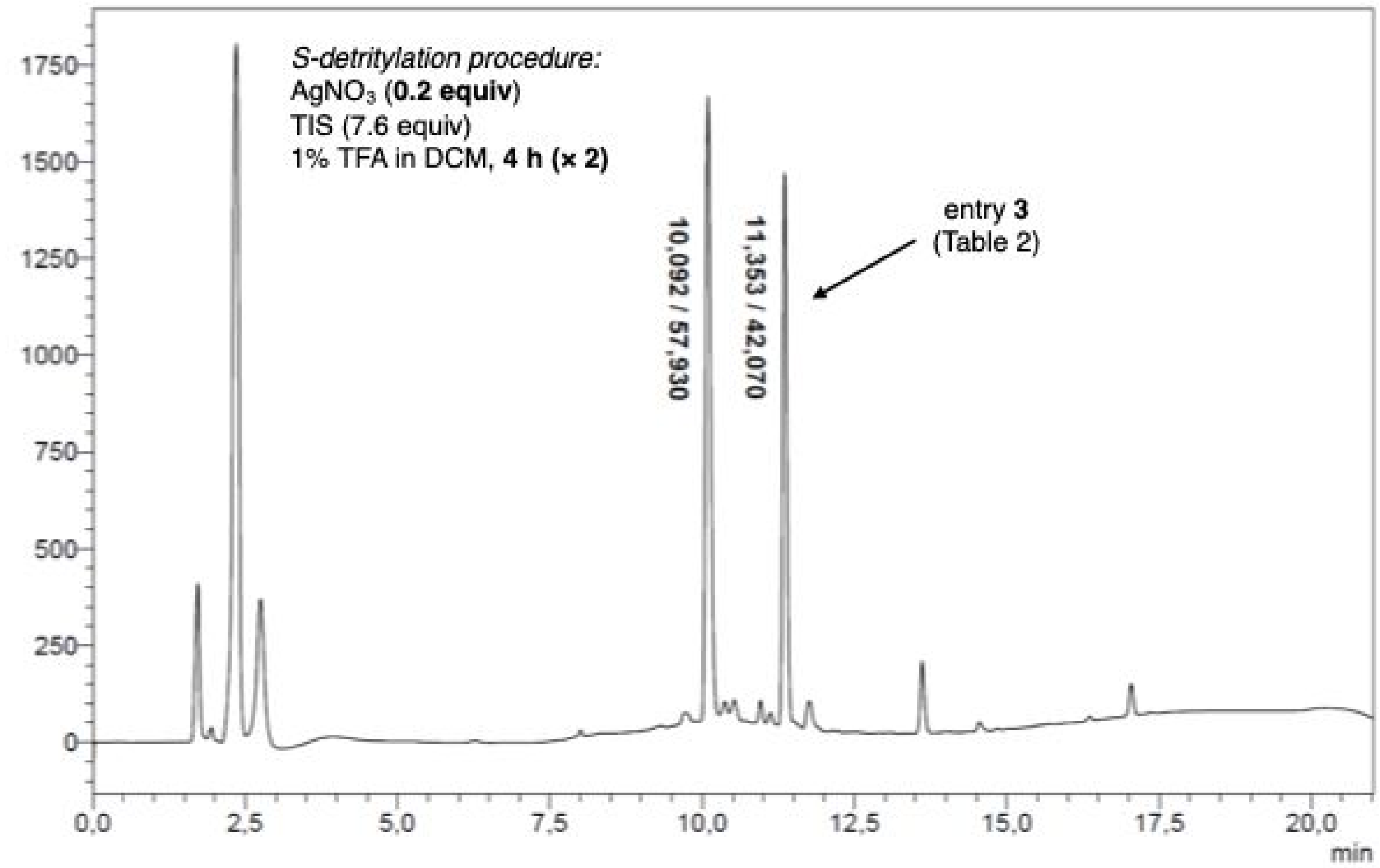

Figure S6. Representative HPLC chromatogram of entry **3** (Table 2) showing 42% conversion to the *S*-benzylated product, t_R= 11.3 min. Analysis was performed using Method **A**, at a flow rate of 1 mL min^{-1}, with UV detection at 220 nm.

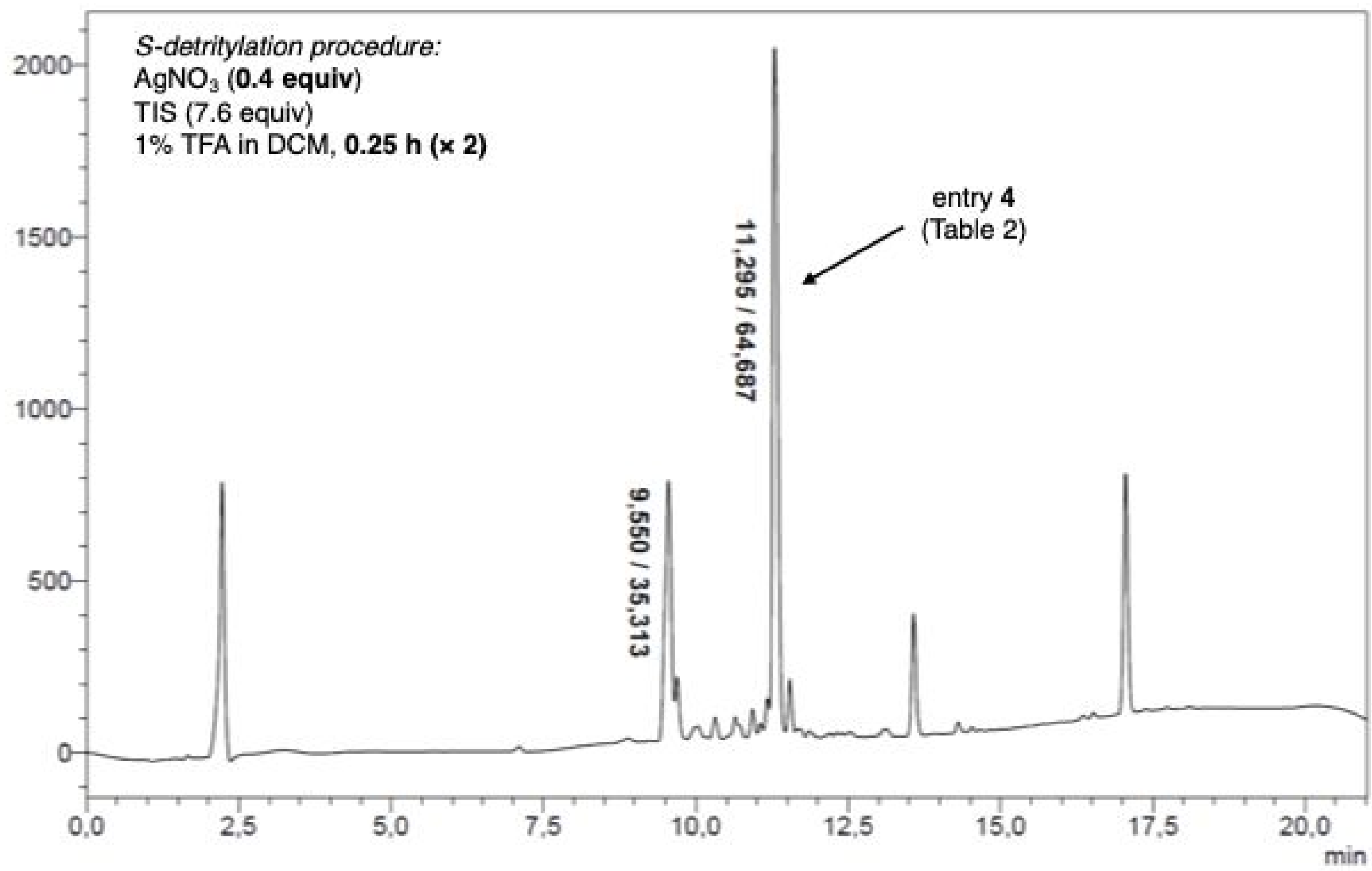

Figure S7. Representative HPLC chromatogram of entry **4** (Table 2) showing 64% conversion to the *S*-benzylated product, t_R= 11.2 min. Analysis was performed using Method **A**, at a flow rate of 1 mL min^{-1}, with UV detection at 220 nm.

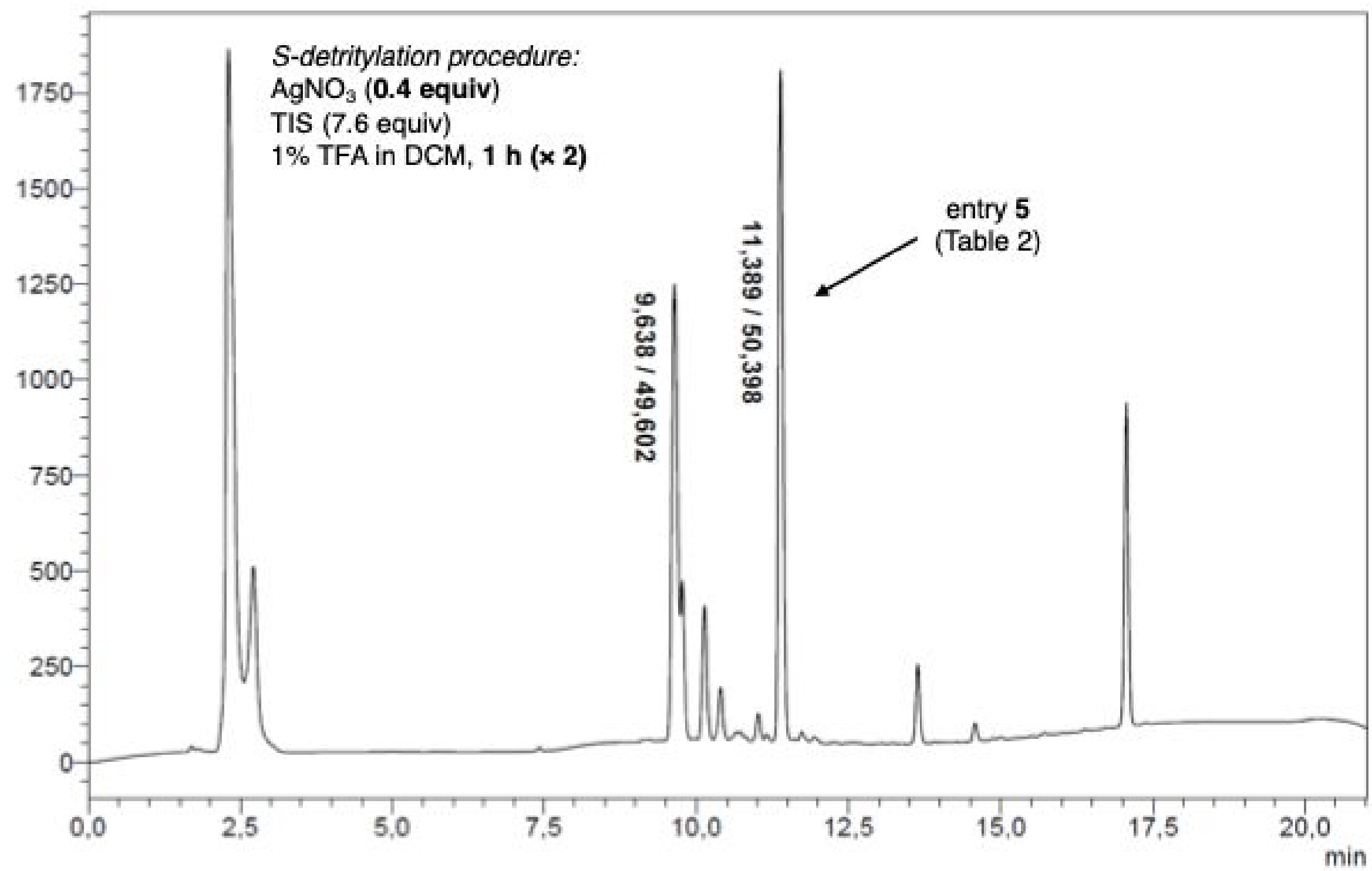

Figure S8. Representative HPLC chromatogram of entry **5** (Table 2) showing 50% conversion to the *S*-benzylated product, t_R= 11.3 min. Analysis was performed using Method **A**, at a flow rate of 1 mL min^{-1}, with UV detection at 220 nm.

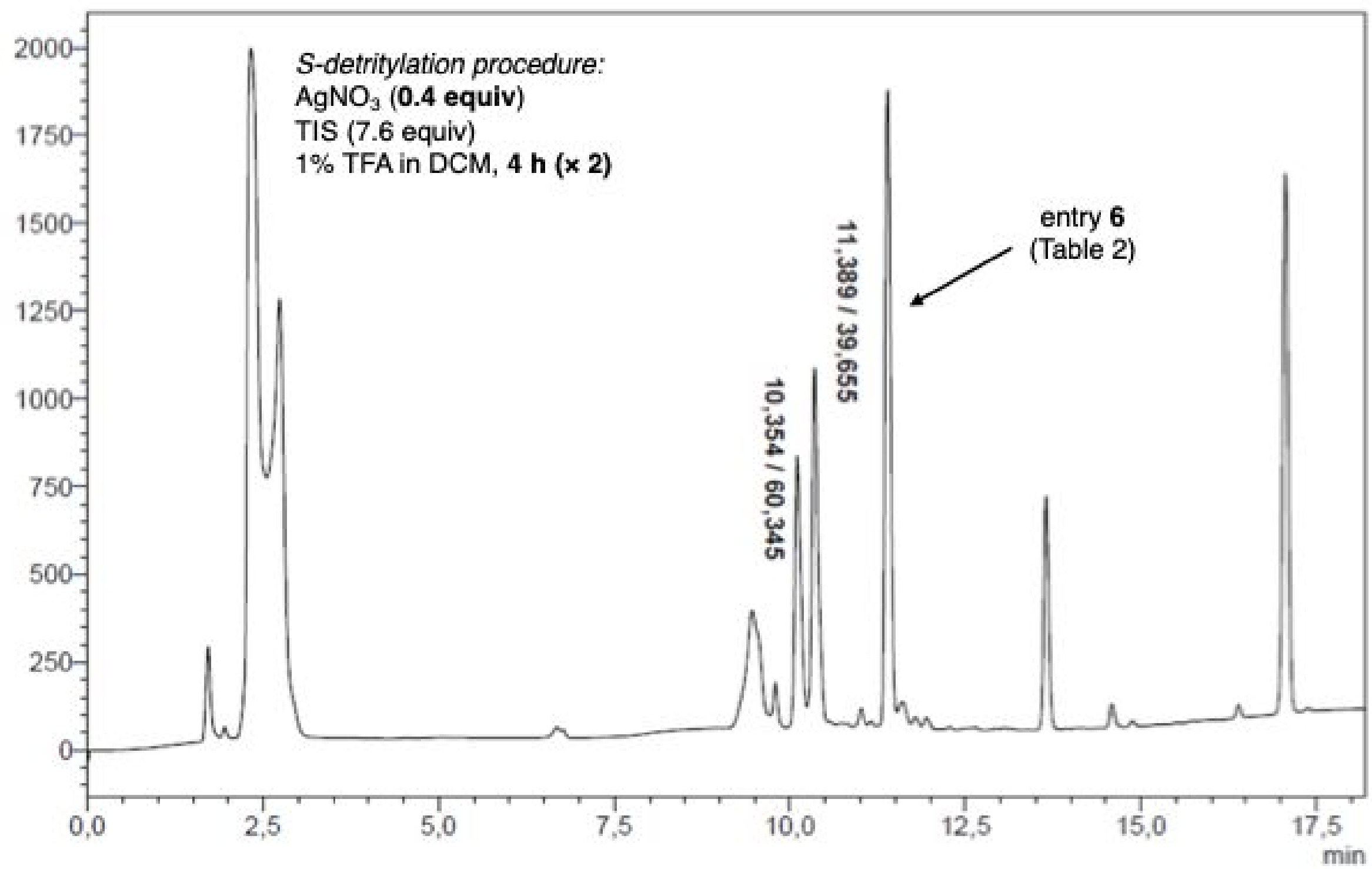

Figure S9. Representative HPLC chromatogram of entry **6** (Table 2) showing 39% conversion to the *S*-benzylated product, t_R= 11.3 min. Analysis was performed using Method **A**, at a flow rate of 1 mL min^{-1}, with UV detection at 220 nm.

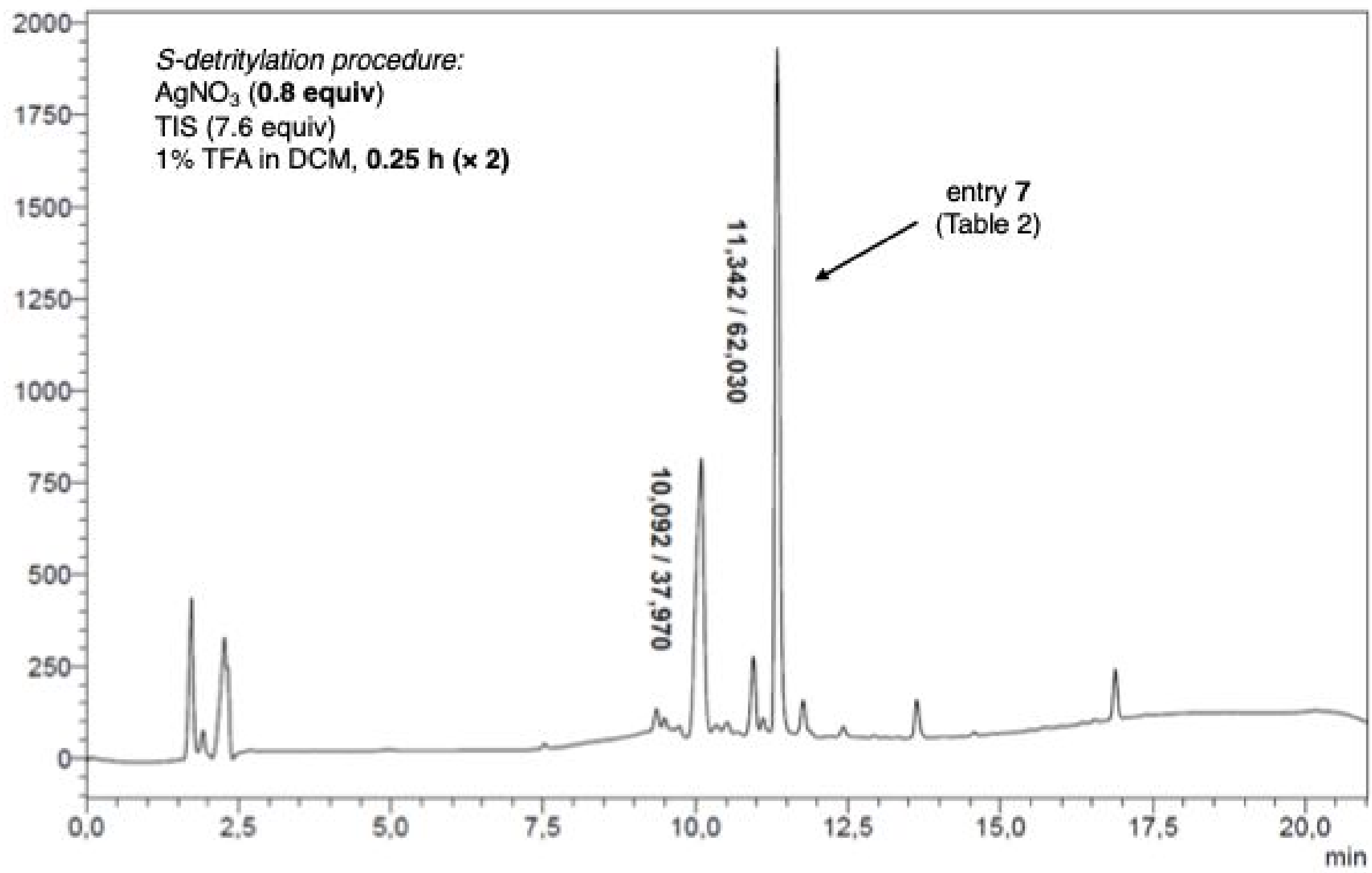

Figure S10. Representative HPLC chromatogram of entry **7** (Table 2) showing 62% conversion to the *S*-benzylated product, t_R= 11.3 min. Analysis was performed using Method **A**, at a flow rate of 1 mL min^{-1}, with UV detection at 220 nm.

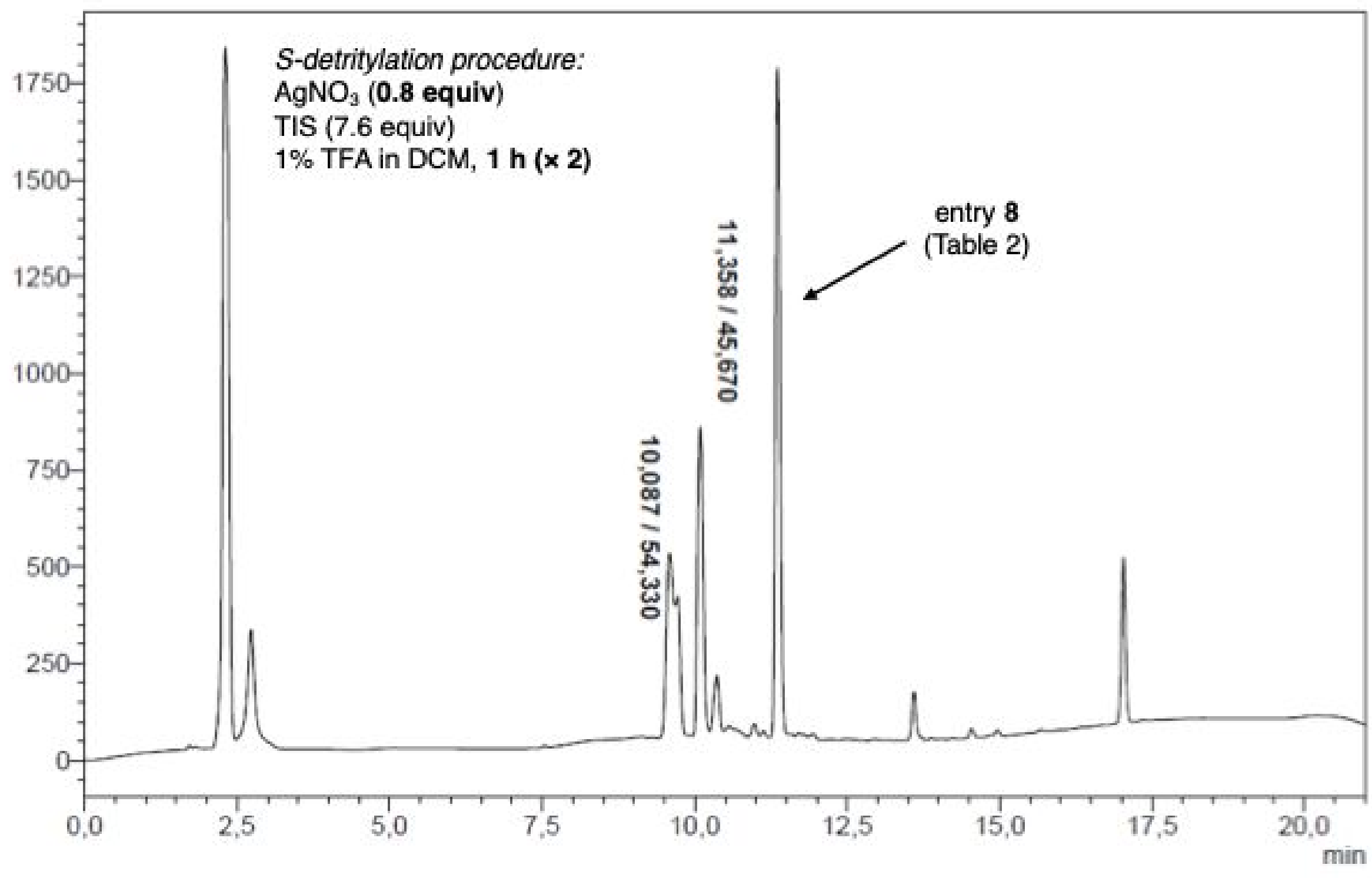

Figure S11. Representative HPLC chromatogram of entry **8** (Table 2) showing 45% conversion to the *S*-benzylated product, t_R= 11.3 min. Analysis was performed using Method **A**, at a flow rate of 1 mL min^{-1}, with UV detection at 220 nm.

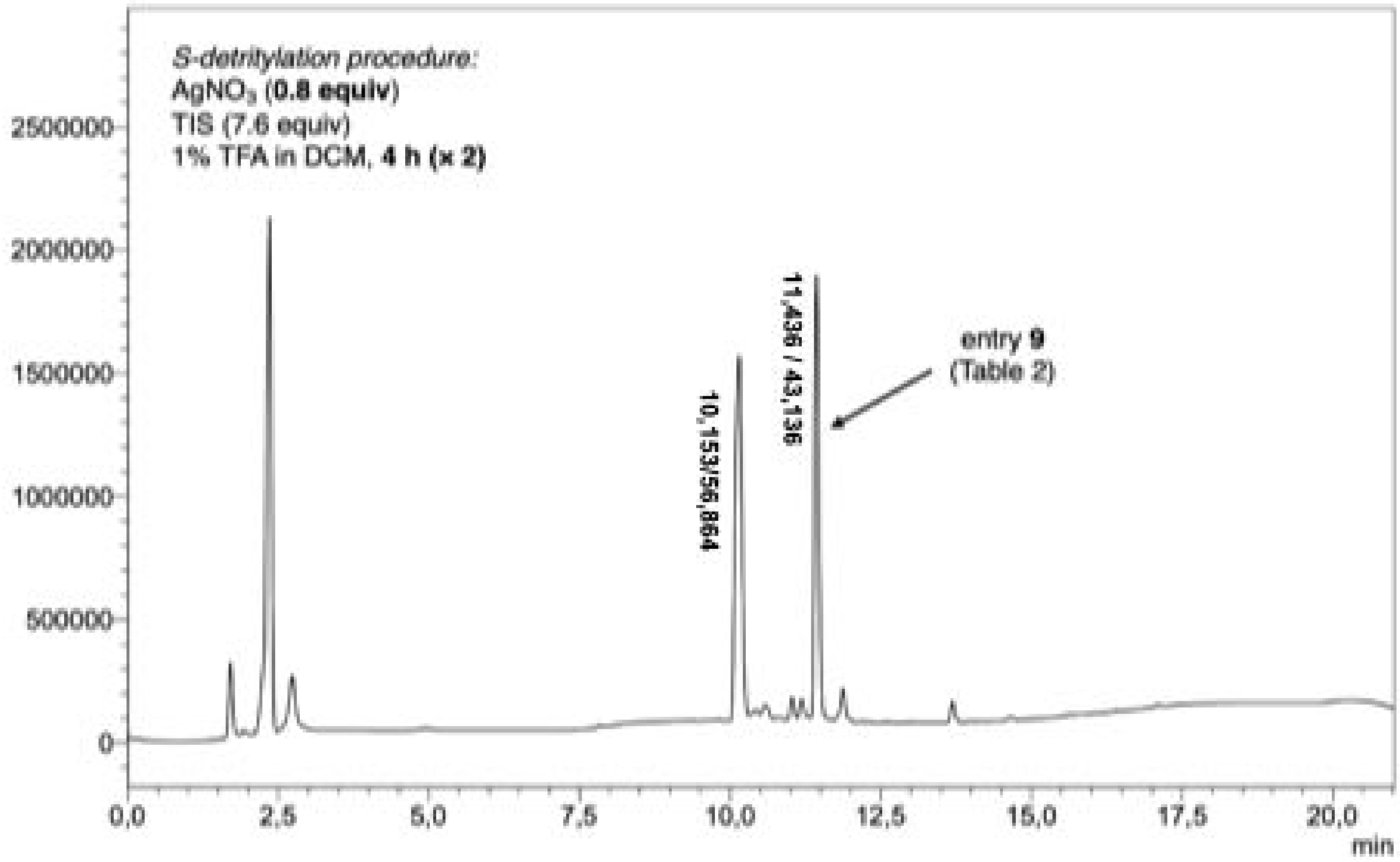

Figure S12. Representative HPLC chromatogram of entry **9** (Table 2) showing 43% conversion to the *S*-benzylated product, t_R= 11.4 min. Analysis was performed using Method **A**, at a flow rate of 1 mL min^{-1}, with UV detection at 220 nm.

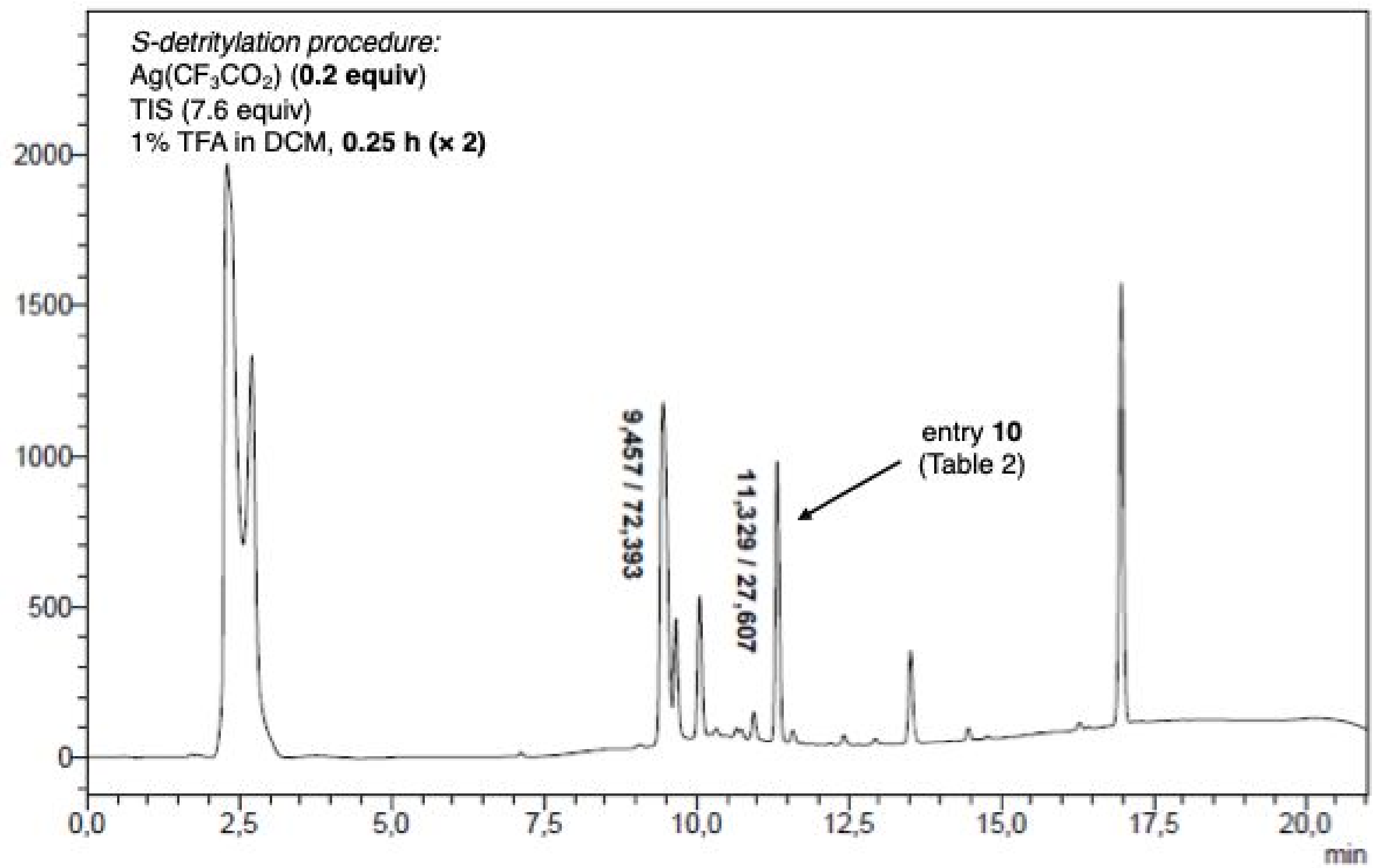

Figure S13. Representative HPLC chromatogram of entry **10** (Table 2) showing 27% conversion to the *S*-benzylated product, t_R= 11.3 min. Analysis was performed using Method **A**, at a flow rate of 1 mL min^{-1}, with UV detection at 220 nm.

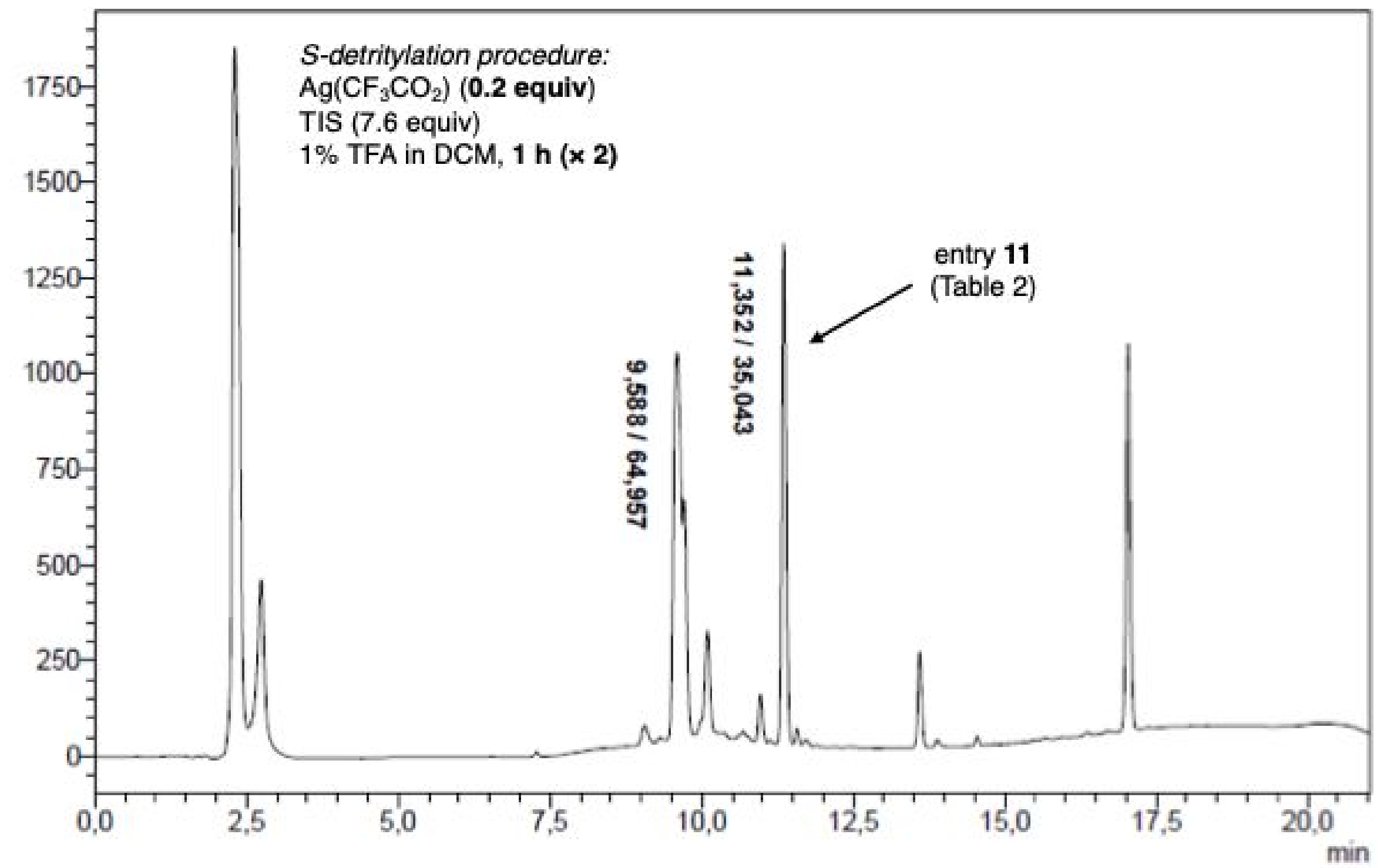

Figure S14. Representative HPLC chromatogram of entry **11** (Table 2) showing 35% conversion to the *S*-benzylated product, t_R= 11.3 min. Analysis was performed using Method **A**, at a flow rate of 1 mL min^{-1}, with UV detection at 220 nm.

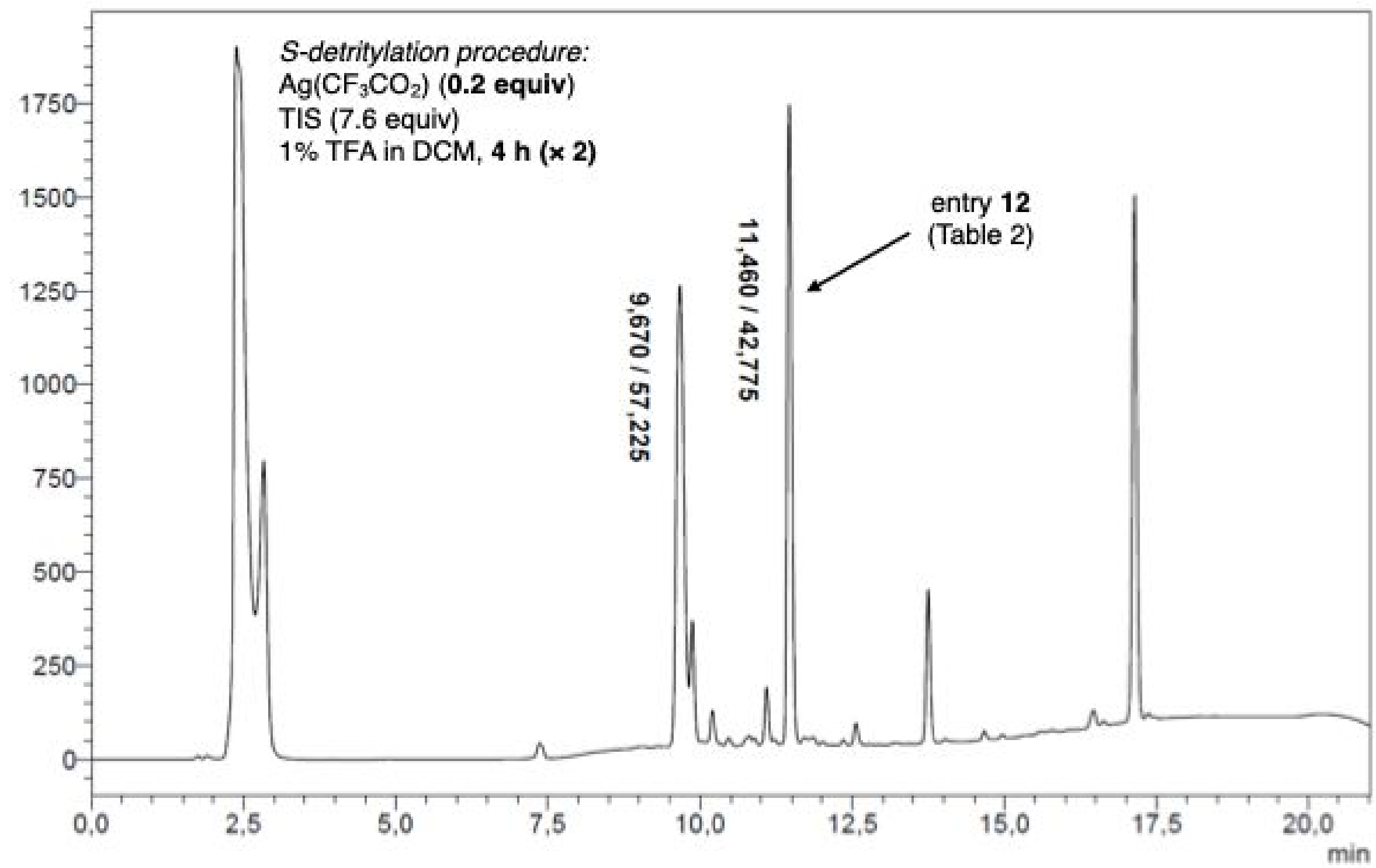

Figure S15. Representative HPLC chromatogram of entry **12** (Table 2) showing 42% conversion to the *S*-benzylated product, t_R= 11.4 min. Analysis was performed using Method **A**, at a flow rate of 1 mL min^{-1}, with UV detection at 220 nm.

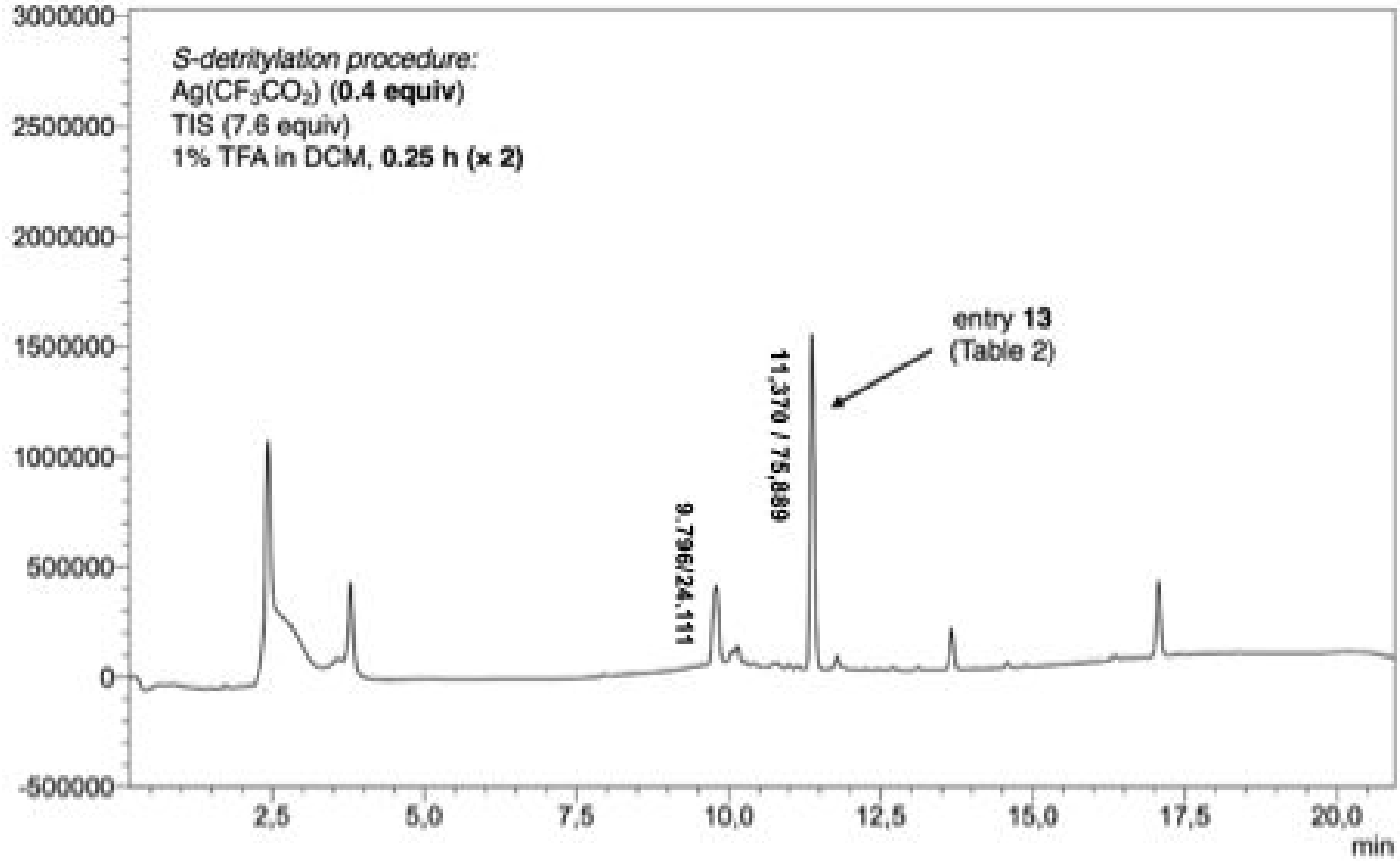

Figure S16. Representative HPLC chromatogram of entry **13** (Table 2) showing 75% conversion to the *S*-benzylated product, t_R= 11.3 min. Analysis was performed using Method **A**, at a flow rate of 1 mL min^{-1}, with UV detection at 220 nm.

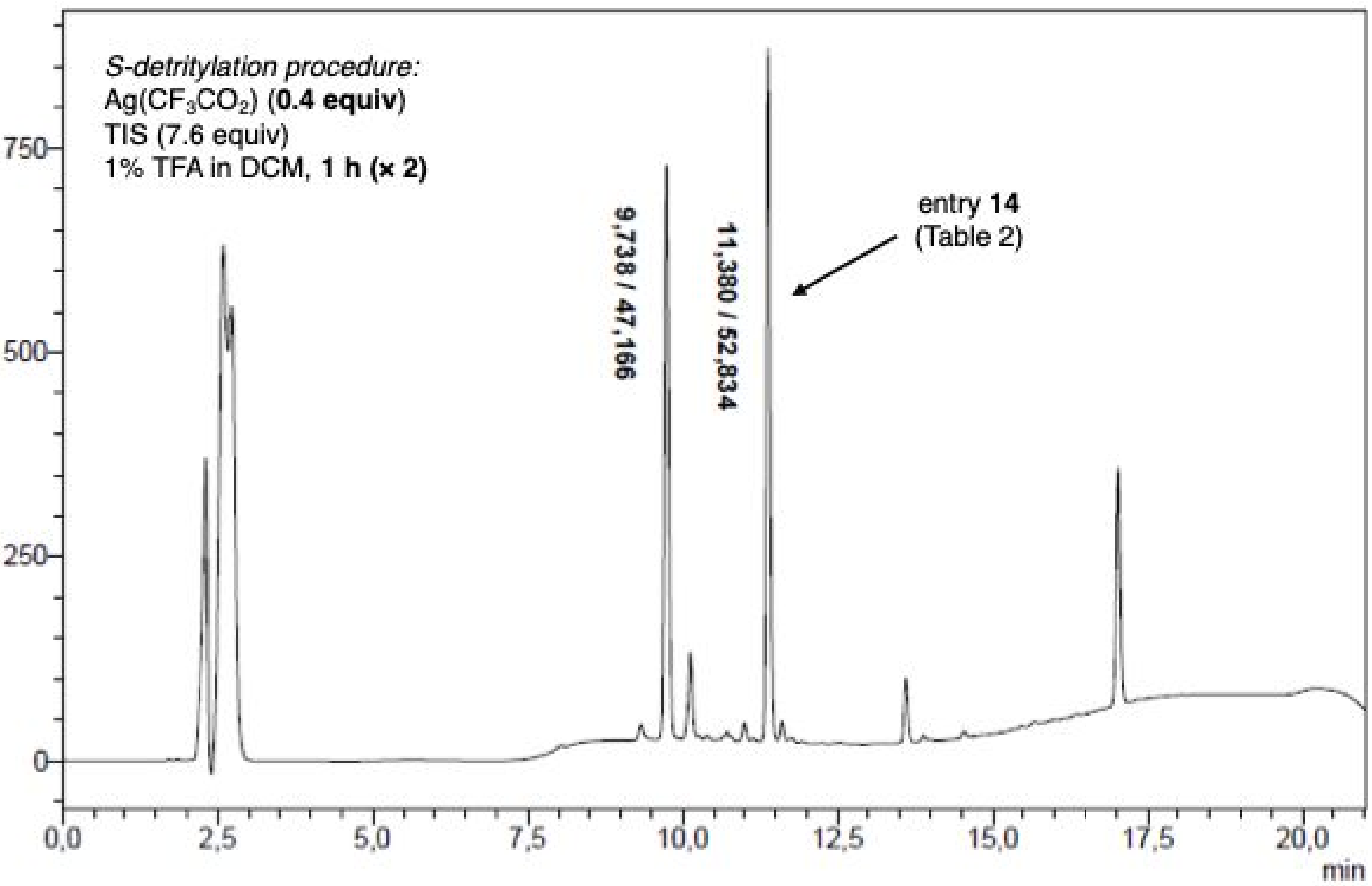

Figure S17. Representative HPLC chromatogram of entry **14** (Table 2) showing 52% conversion to the *S*-benzylated product, t_R= 11.3 min. Analysis was performed using Method **A**, at a flow rate of 1 mL min^{-1}, with UV detection at 220 nm.

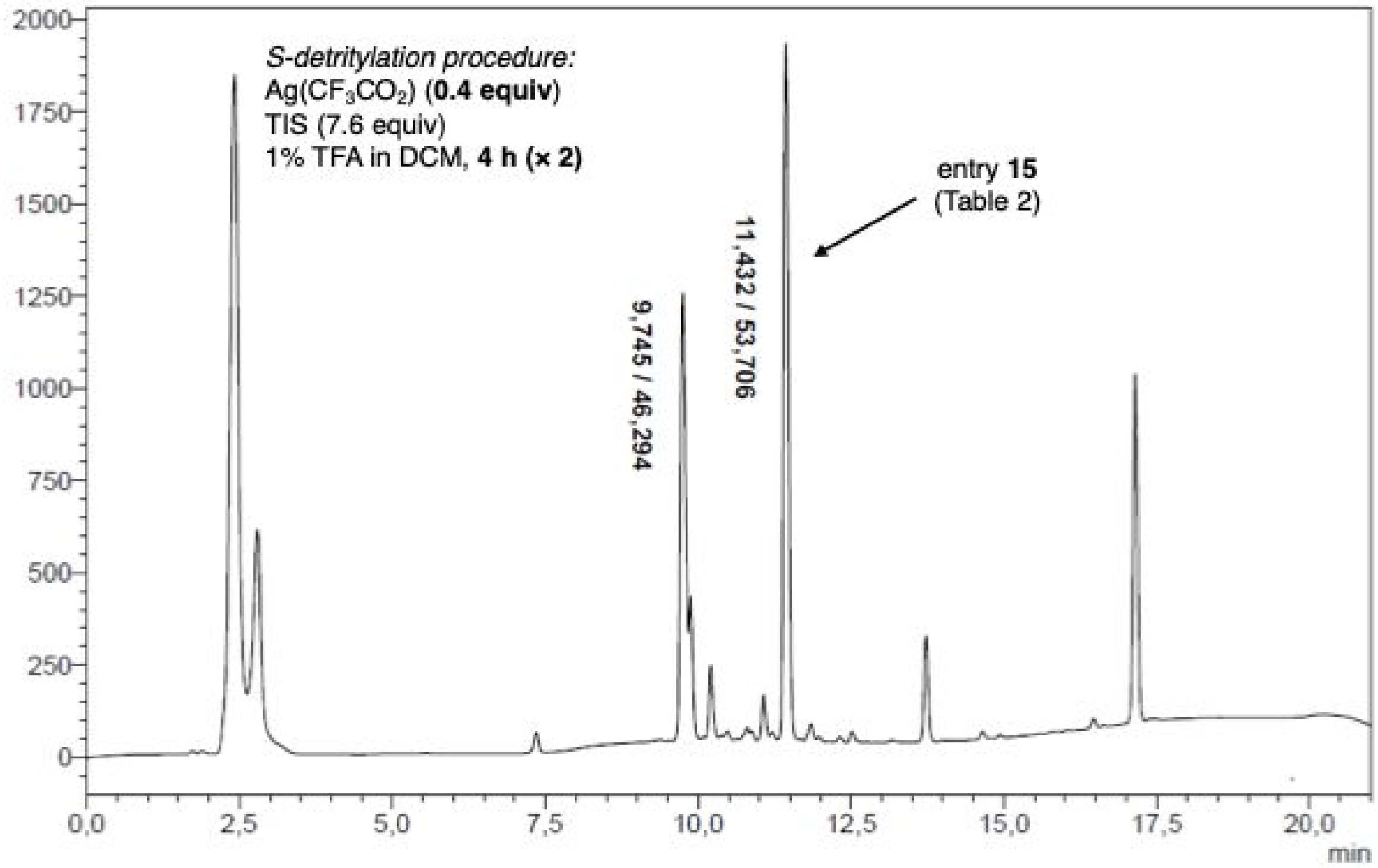

Figure S18. Representative HPLC chromatogram of entry **15** (Table 2) showing 53% conversion to the *S*-benzylated product, t_R= 11.4 min. Analysis was performed using Method **A**, at a flow rate of 1 mL min^{-1}, with UV detection at 220 nm.

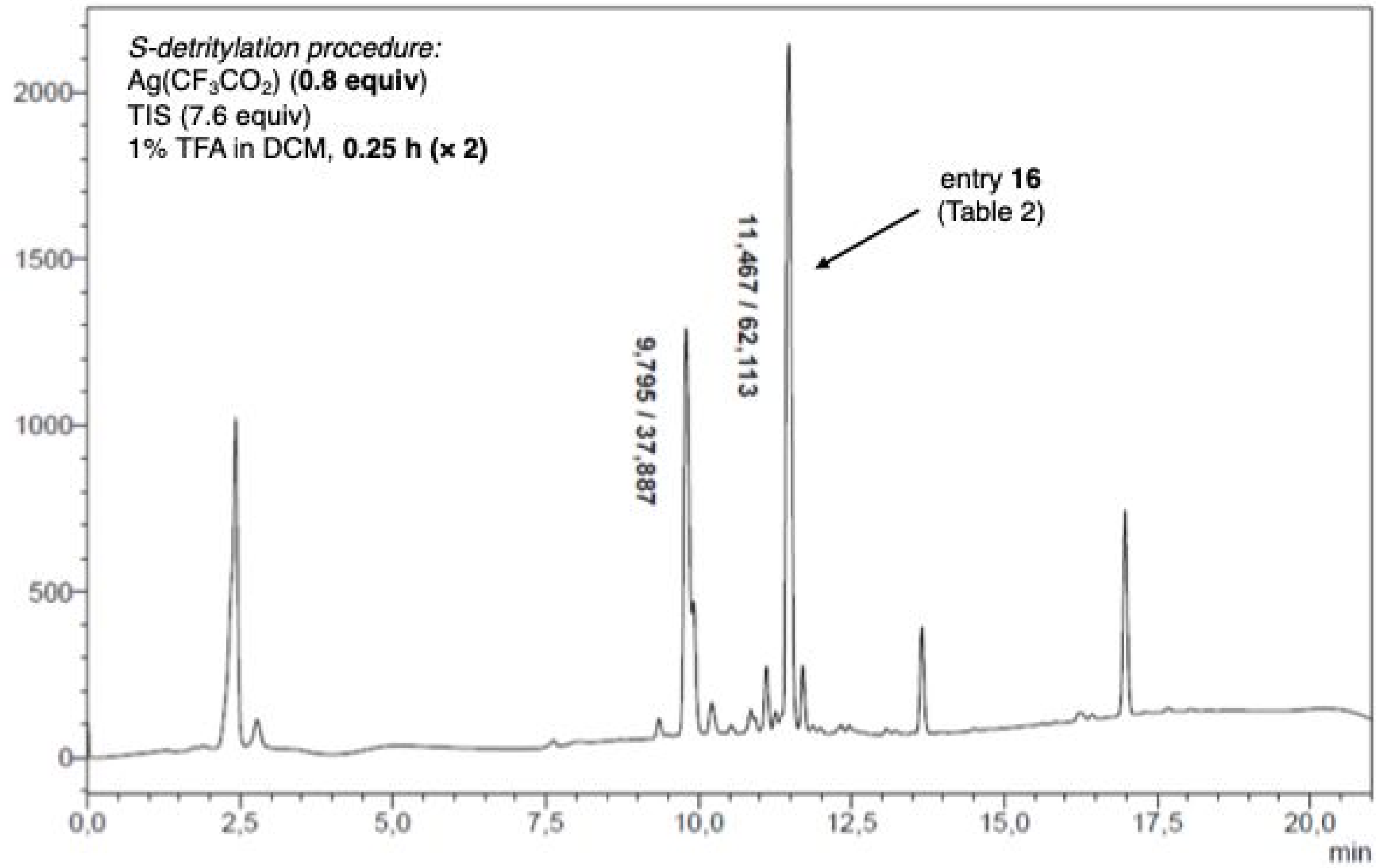

Figure S19. Representative HPLC chromatogram of entry **16** (Table 2) showing 62% conversion to the *S*-benzylated product, t_R= 11.4 min. Analysis was performed using Method A, at a flow rate of 1 mL min^{-1}, with UV detection at 220 nm.

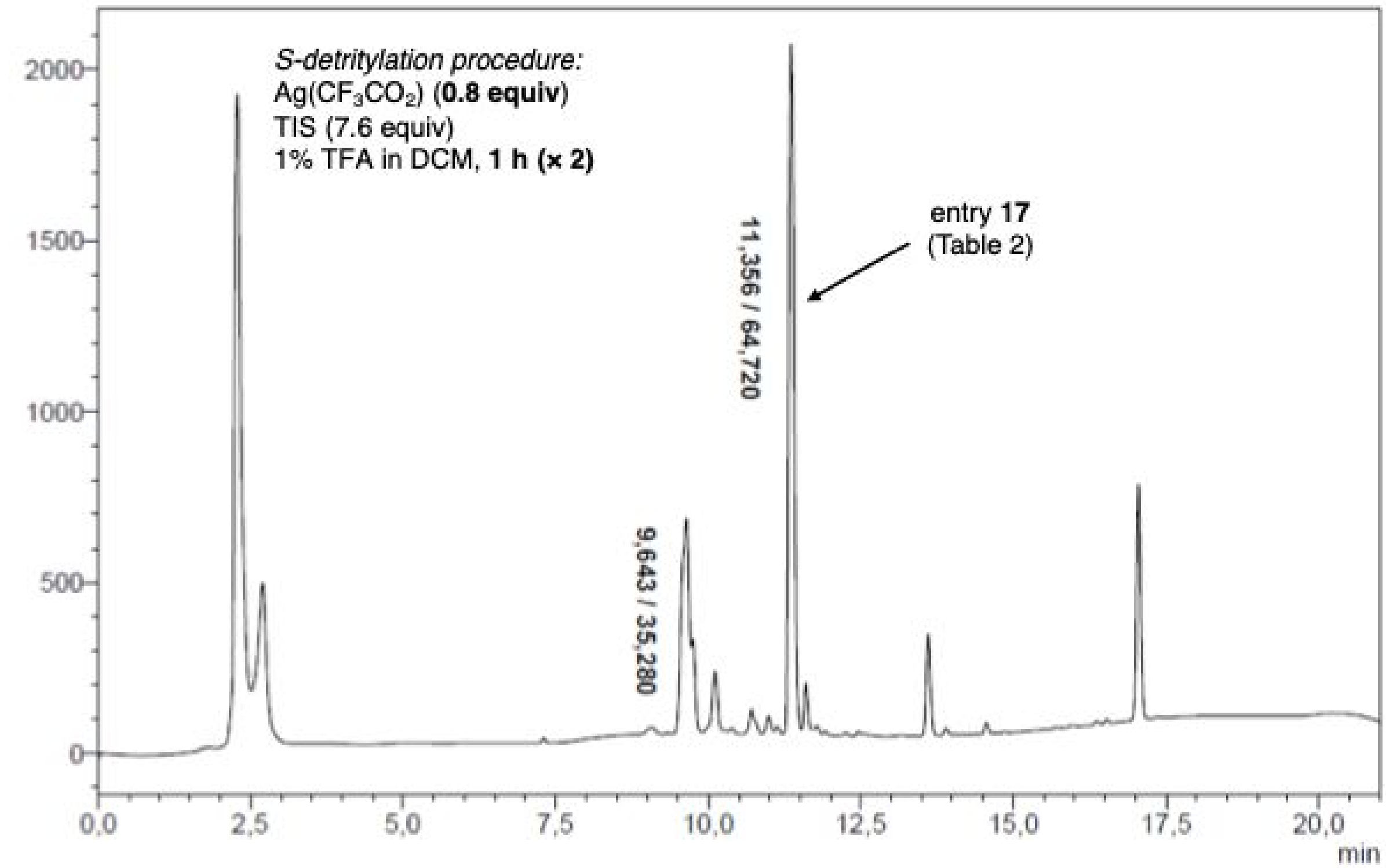

Figure S20. Representative HPLC chromatogram of entry **17** (Table 2) showing 64% conversion to the *S*-benzylated product, t_R= 11.3 min. Analysis was performed using Method **A**, at a flow rate of 1 mL min^{-1}, with UV detection at 220 nm.

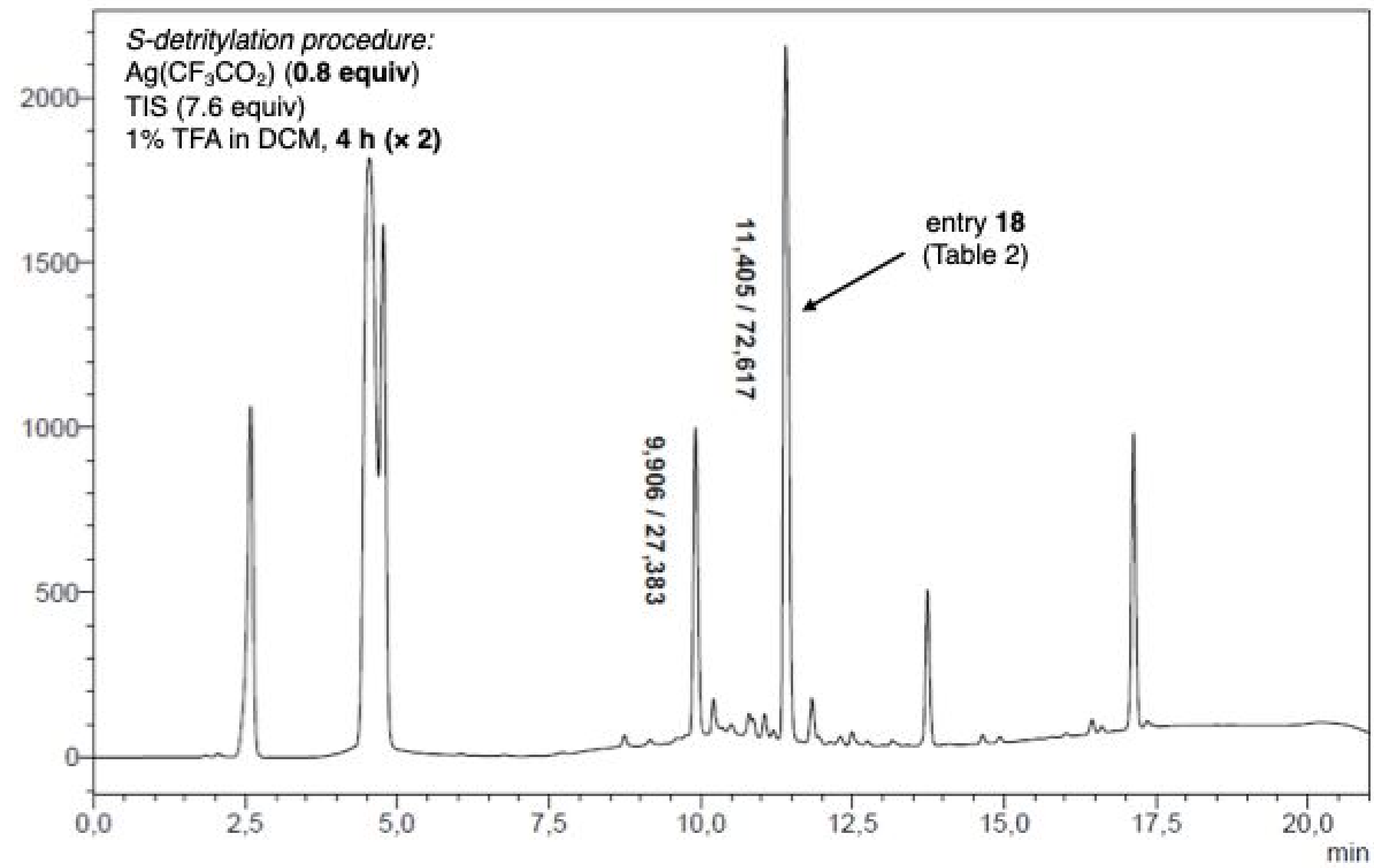

Figure S21. Representative HPLC chromatogram of entry **18** (Table 2) showing 72% conversion to the *S*-benzylated product, t_R= 11.4 min. Analysis was performed using Method **A**, at a flow rate of 1 mL min^{-1}, with UV detection at 220 nm.

*5. HPLC chromatograms for entries **1-7** (Table 3)*

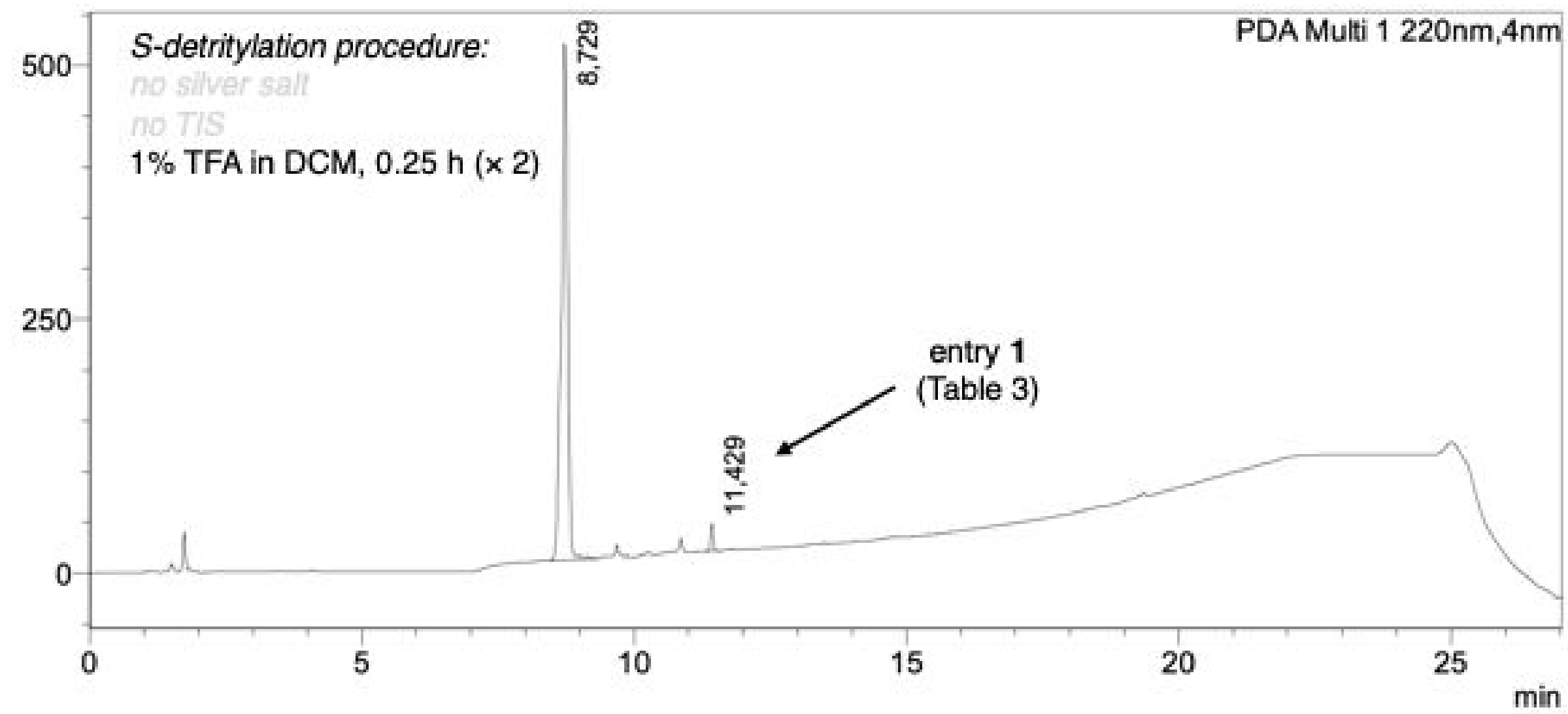

Figure S22. Representative HPLC chromatogram of entry **1** (Table 3) showing <5% conversion to the *S*-benzylated product, t_R= 11.4 min. Analysis was performed using Method **A**, at a flow rate of 1 mL min^{-1}, with UV detection at 220 nm.

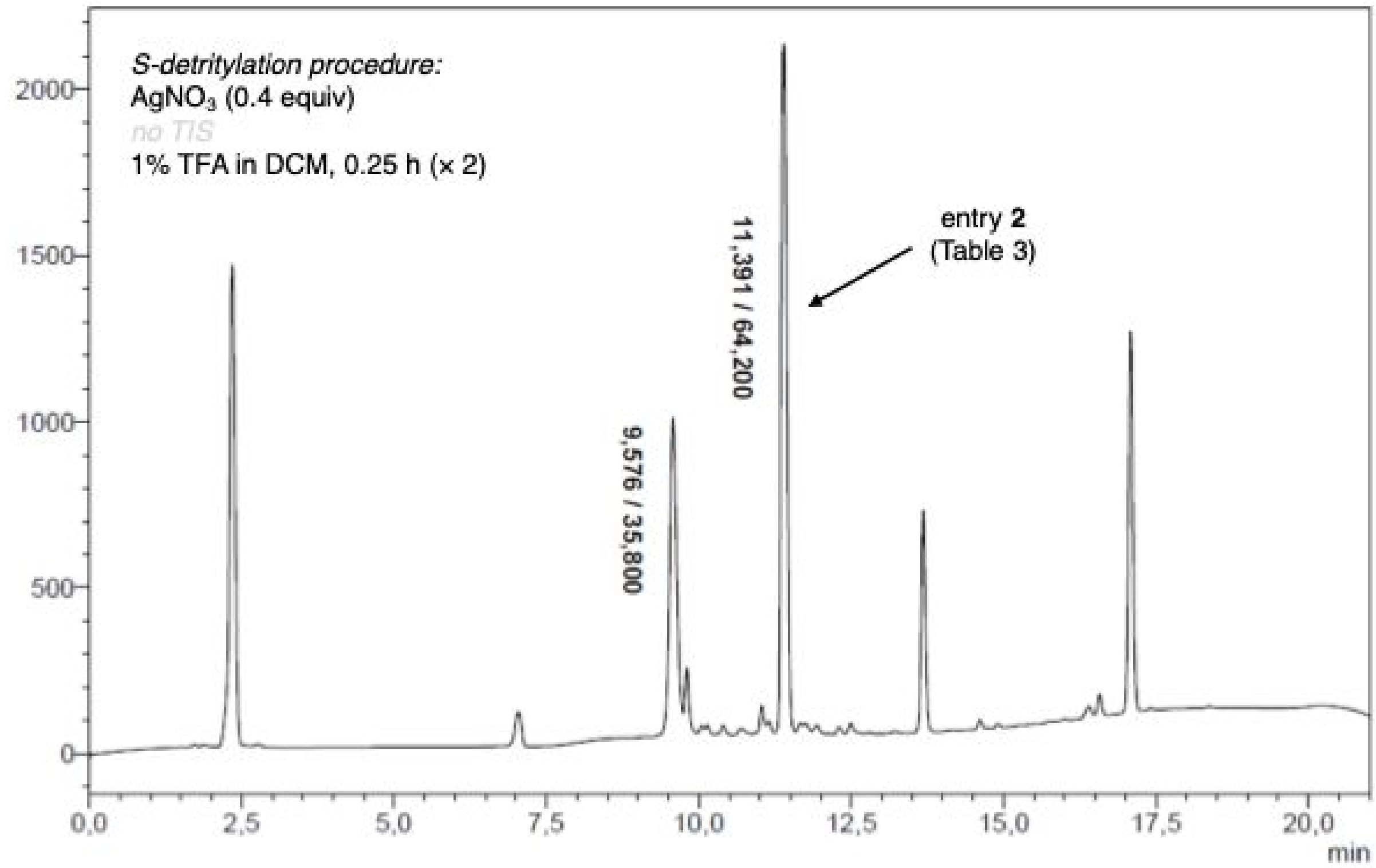

Figure S23. Representative HPLC chromatogram of entry **2** (Table 3) showing 64% conversion to the *S*-benzylated product, t_R= 11.3 min. Analysis was performed using Method **A**, at a flow rate of 1 mL min^{-1}, with UV detection at 220 nm.

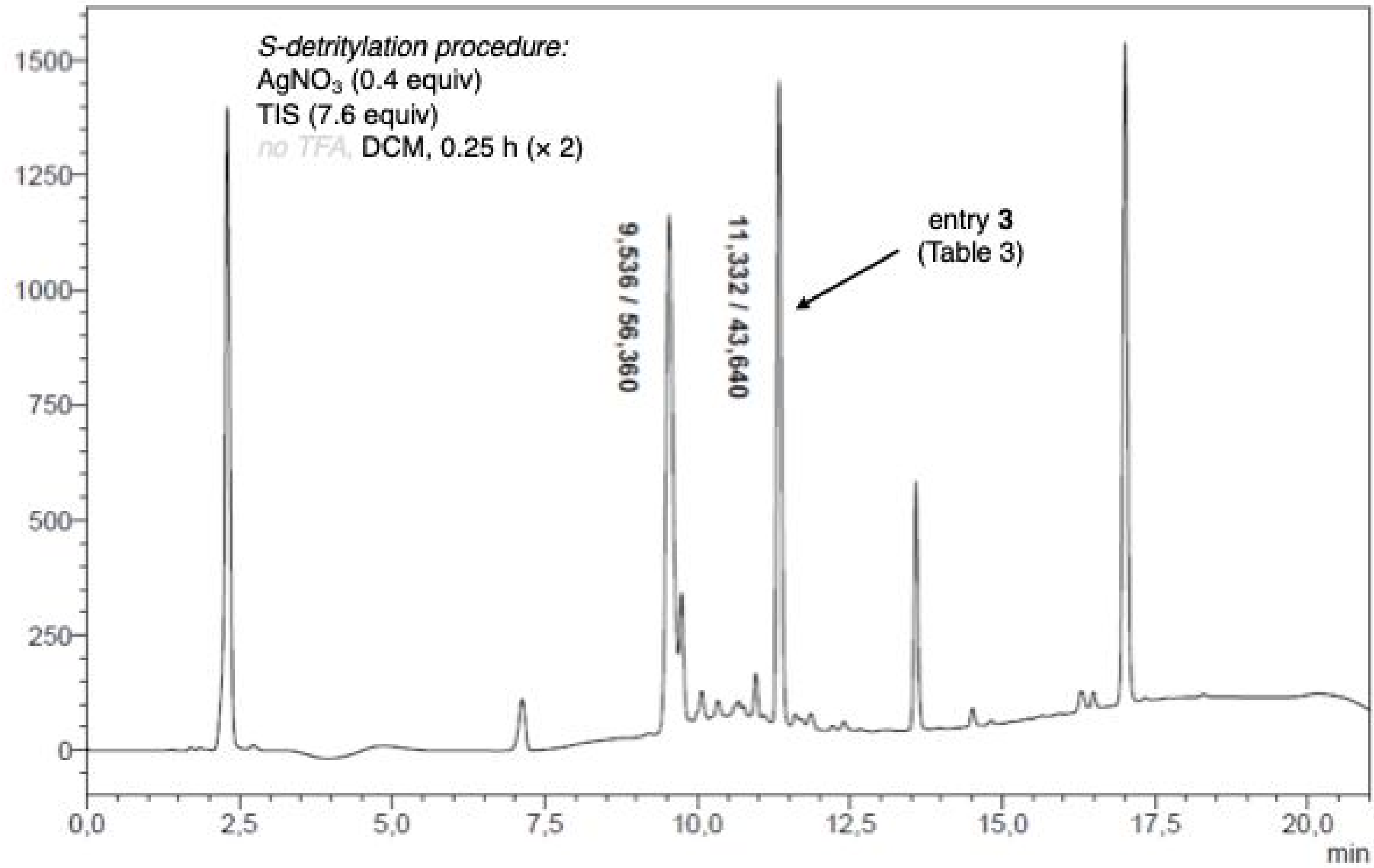

Figure S24. Representative HPLC chromatogram of entry **3** (Table 3) showing 43% conversion to the *S*-benzylated product, t_R= 11.3 min. Analysis was performed using Method **A**, at a flow rate of 1 mL min^{-1}, with UV detection at 220 nm.

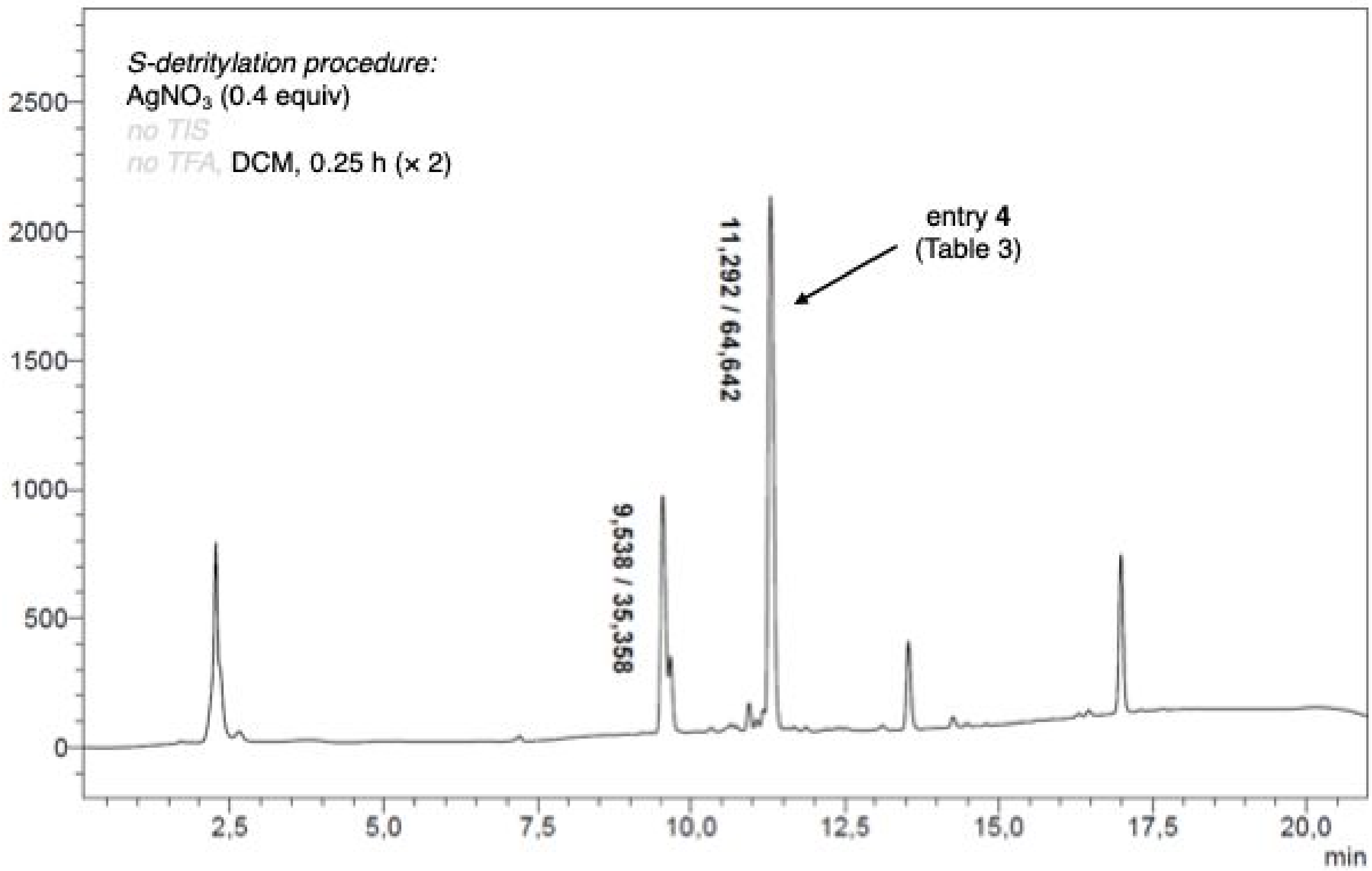

Figure S25. Representative HPLC chromatogram of entry **4** (Table 3) showing 64% conversion to the *S*-benzylated product, t_R= 11.3 min. Analysis was performed using Method **A**, at a flow rate of 1 mL min^{-1}, with UV detection at 220 nm.

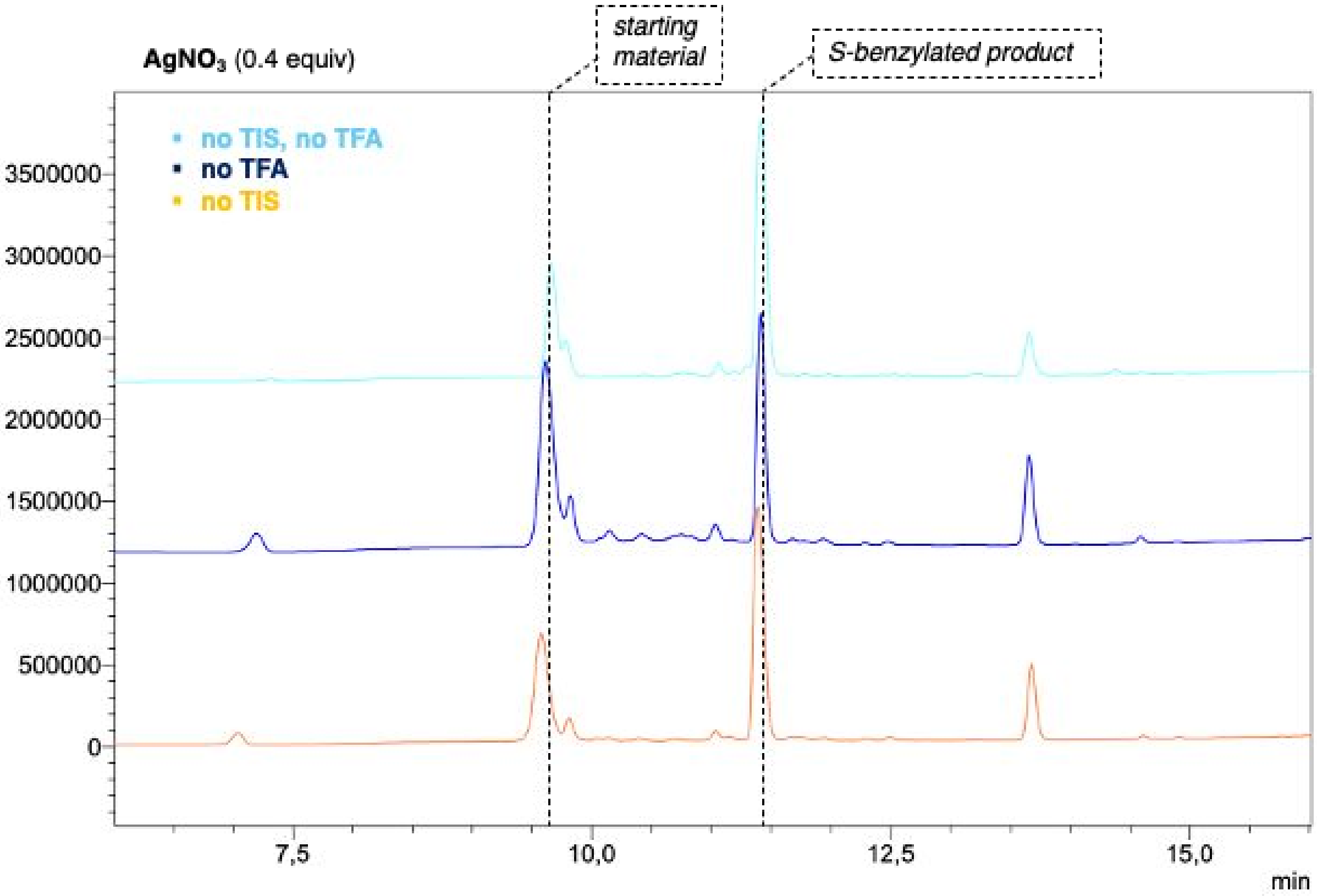

Figure S26. Overlap of chromatograms from entries **2-4** Table 3.

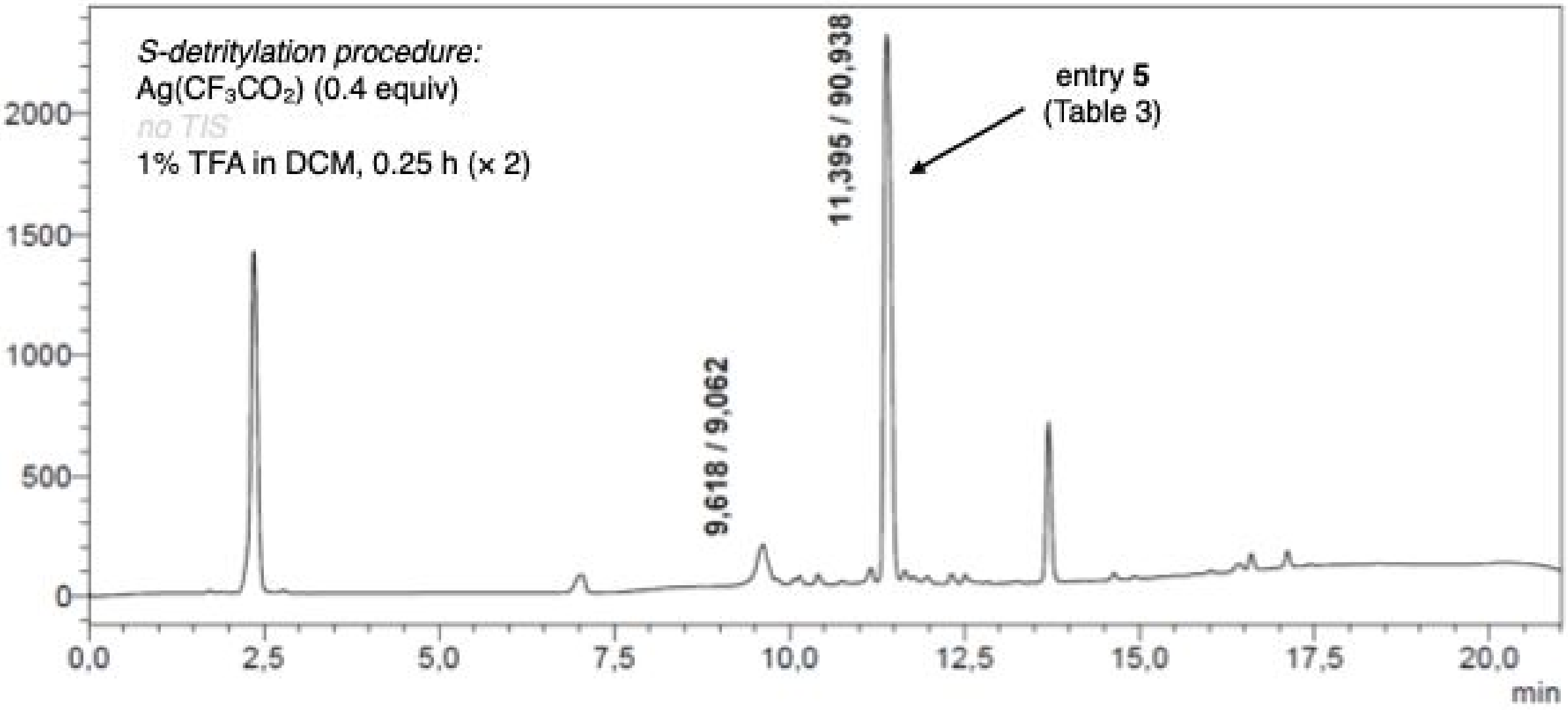

Figure S27. Representative HPLC chromatogram of entry **5** (Table 3) showing 90% conversion to the *S*-benzylated product, t_R= 11.3 min. Analysis was performed using Method A, at a flow rate of 1 mL min^{-1}, with UV detection at 220 nm.

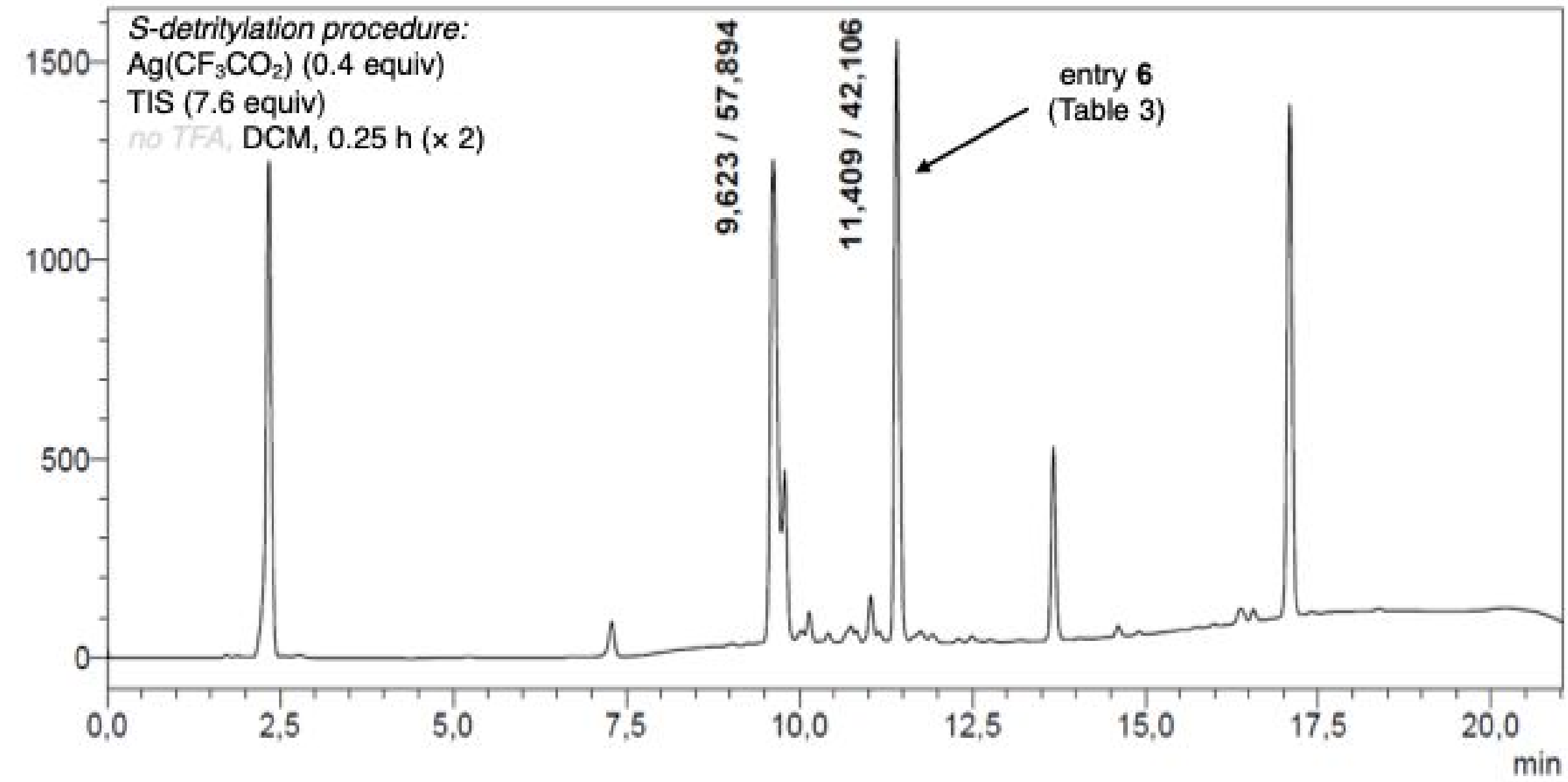

Figure S28. Representative HPLC chromatogram of entry **6** (Table 3) showing 42% conversion to the *S*-benzylated product, t_R= 11.4 min. Analysis was performed using Method **A**, at a flow rate of 1 mL min^{-1}, with UV detection at 220 nm.

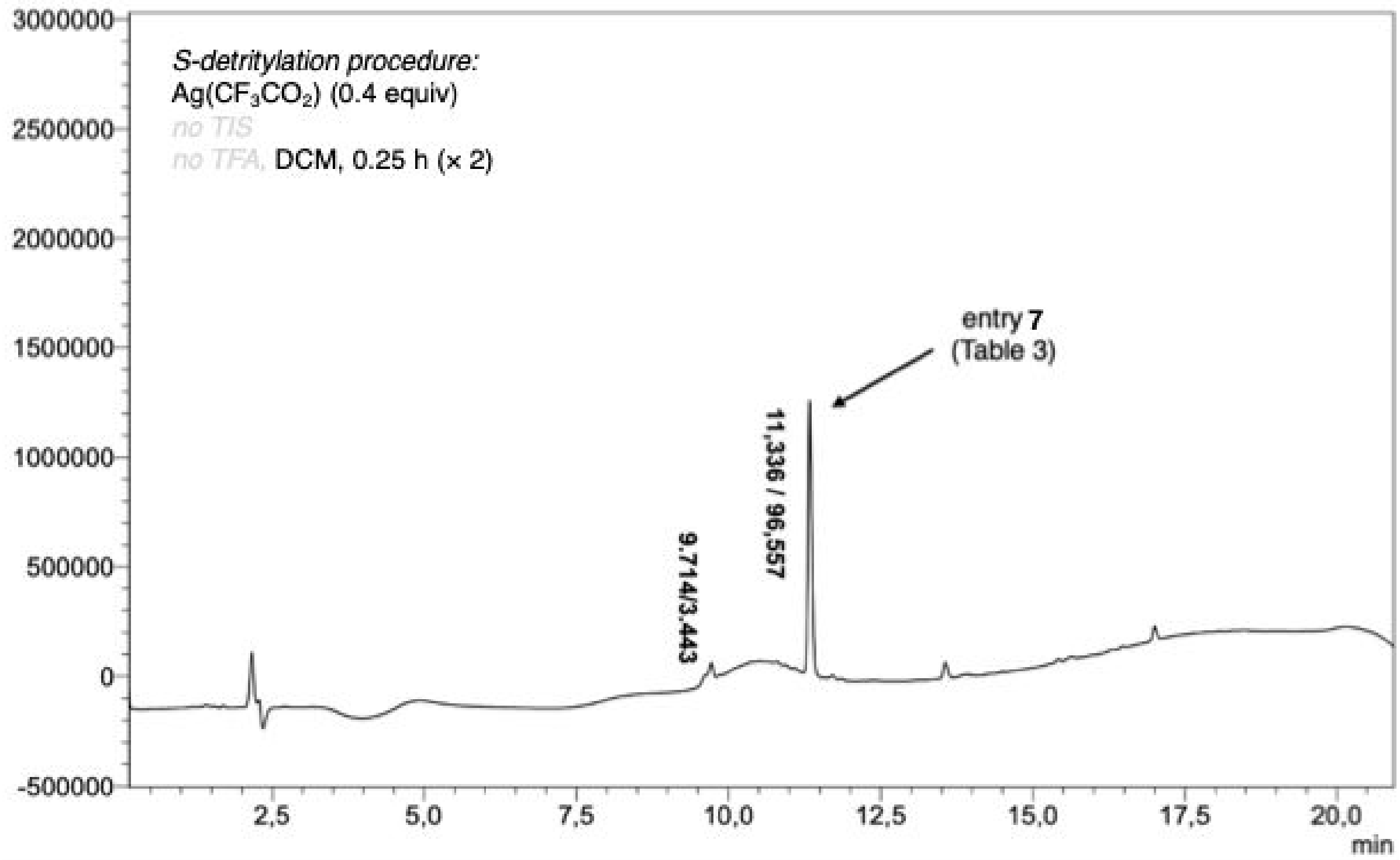

Figure S29. Representative HPLC chromatogram of entry **7** (Table 3) showing 96% conversion to the *S*-benzylated product, t_R= 11.3 min. Analysis was performed using Method **A**, at a flow rate of 1 mL min^{-1}, with UV detection at 220 nm.

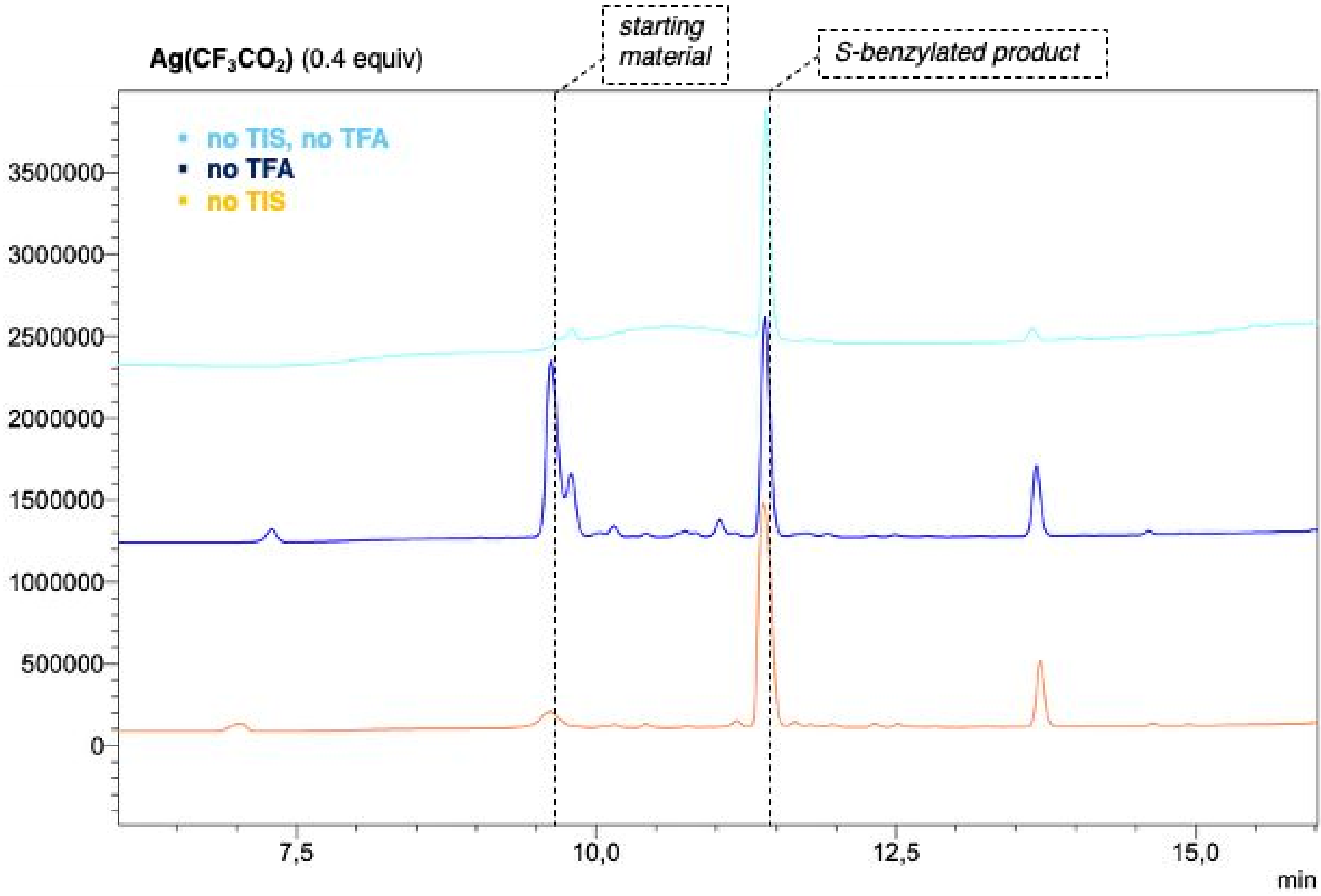

Figure S30. Overlap of chromatograms from entries **5-7** Table 3.

*6. LC-MS and HRMS spectra of S-detritylation reaction mixture residue obtained from the protocol of entry **7**, Table 3*

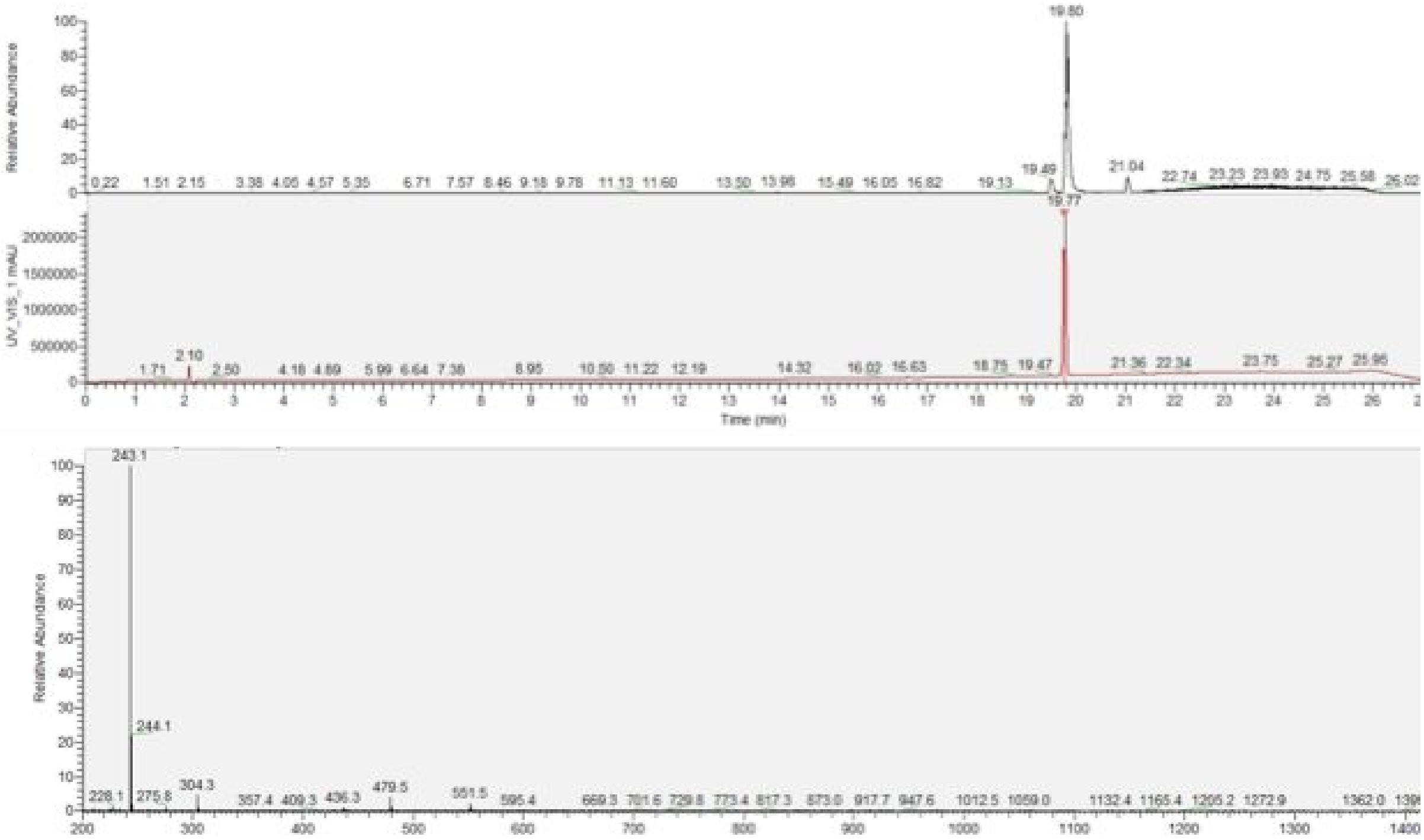

Figure S31. LC-MS of filtrates from entry **7** Table 3 (Method **B**, at a flow rate of 0.8 mL min^{-1}, with UV detection at 220 nm, t_R= 19.7 min).

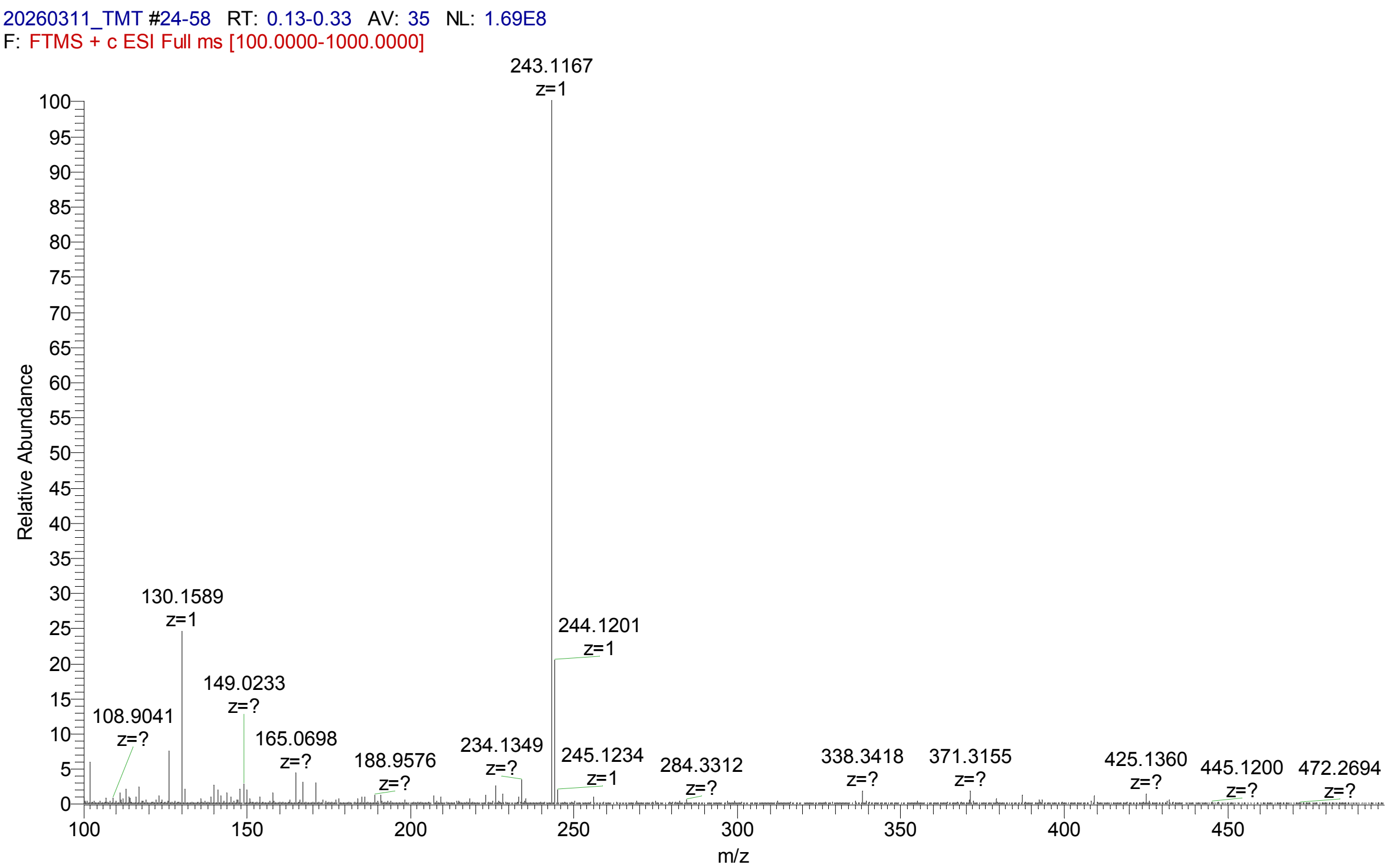

Figure S32. HRMS of filtrates from entry **7** Table 3.

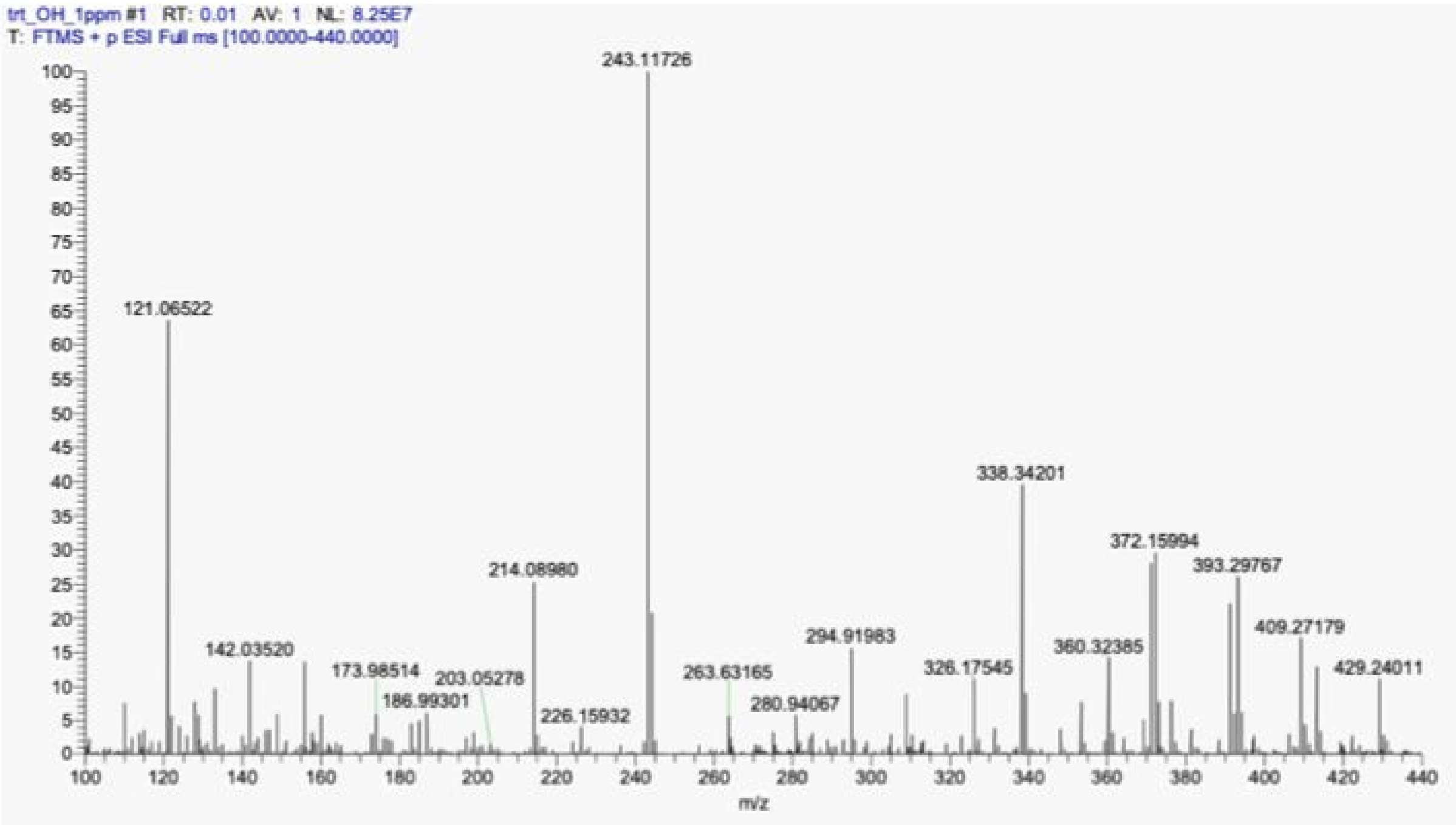

Figure S33. HRMS of commercially available Trt-OH.

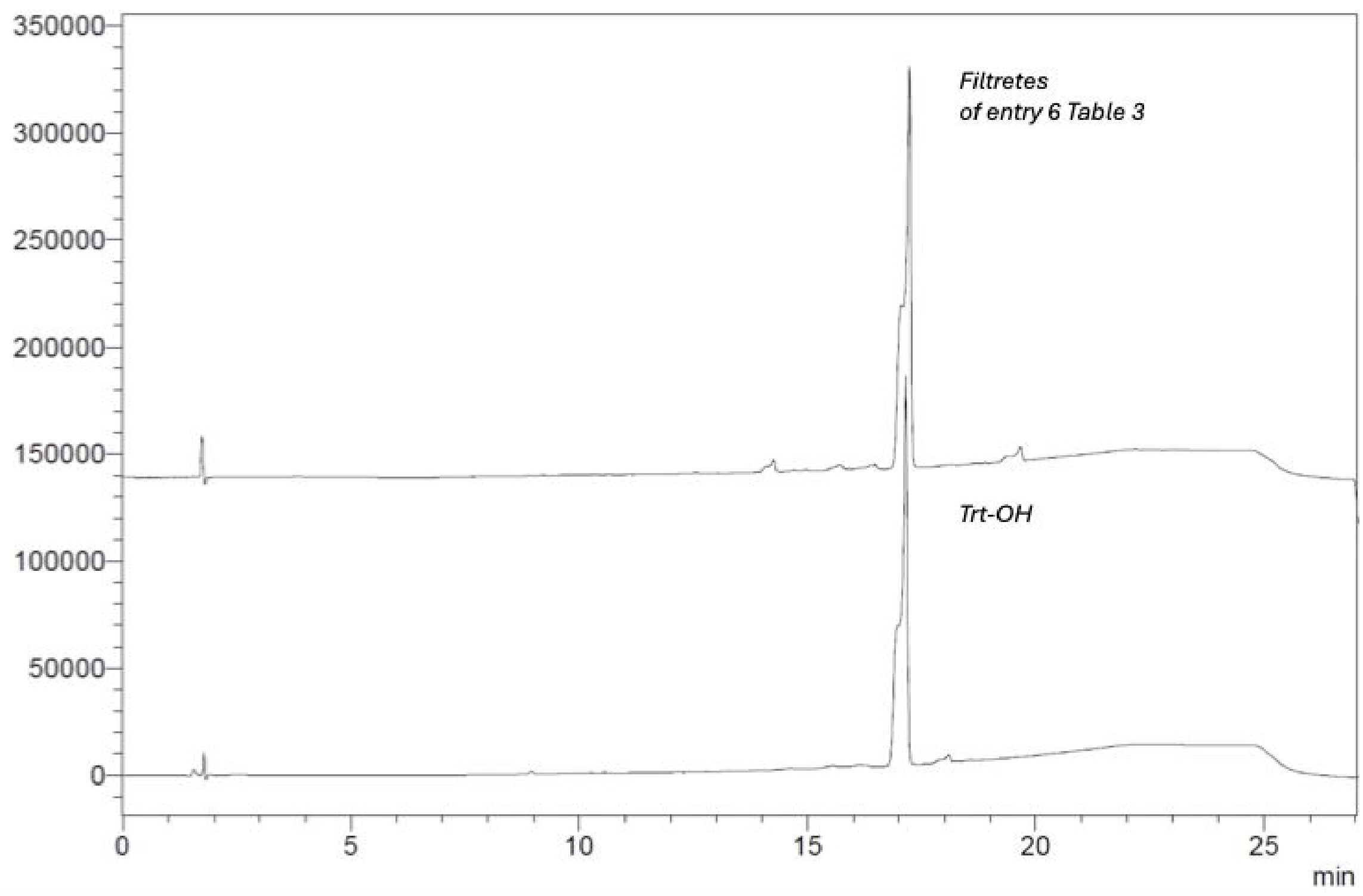

Figure S34. Overlap of chromatograms from filtrates from entry **7** Table 3 and commercially available Trt-OH. Both chromatograms were acquired using Method **B**, at a flow rate of 1 mL min^{-1}, with UV detection at 220 nm.

*7. NMR spectra of S-detritylation reaction mixture residue obtained from the protocol of entry **7**, Table 3*

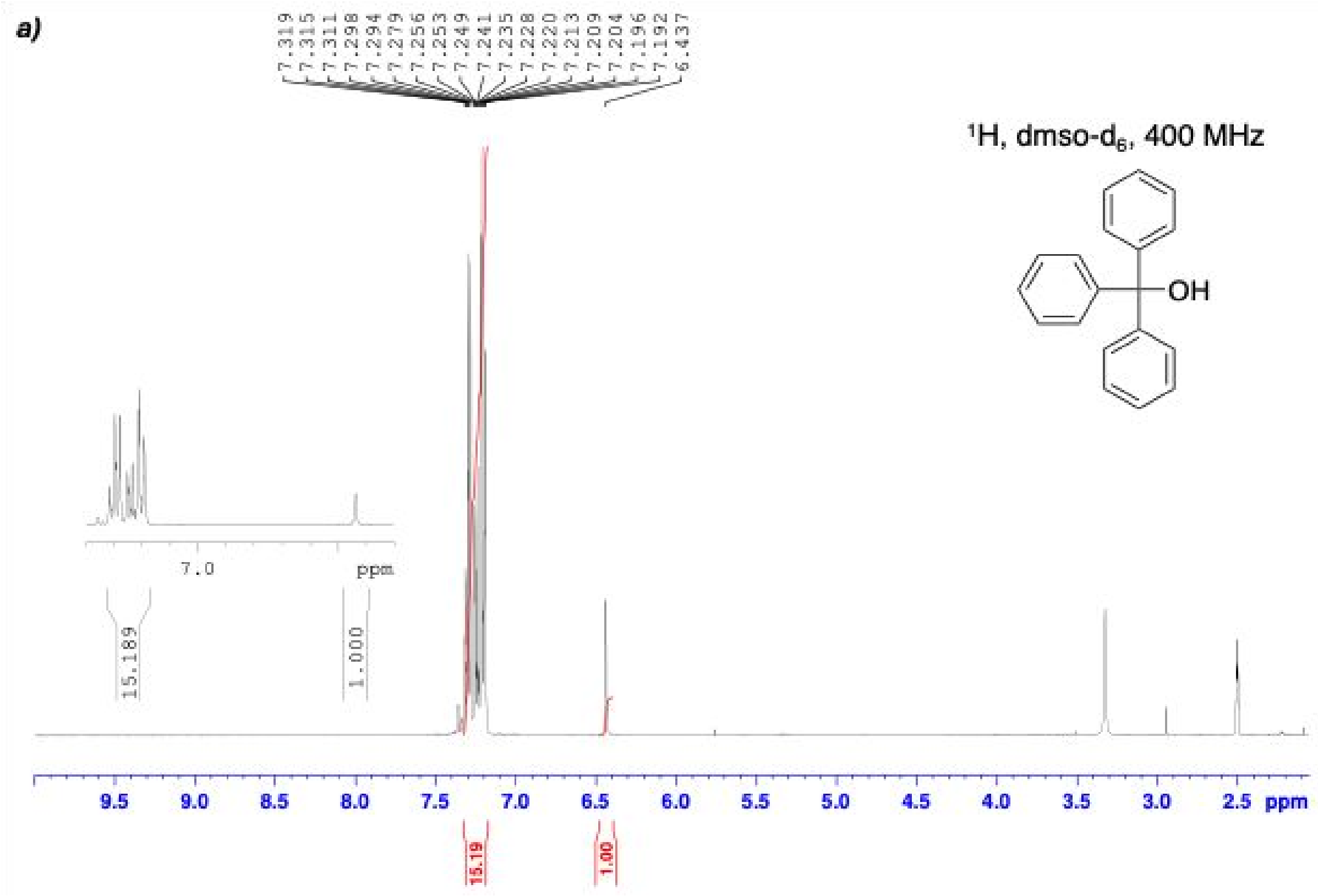

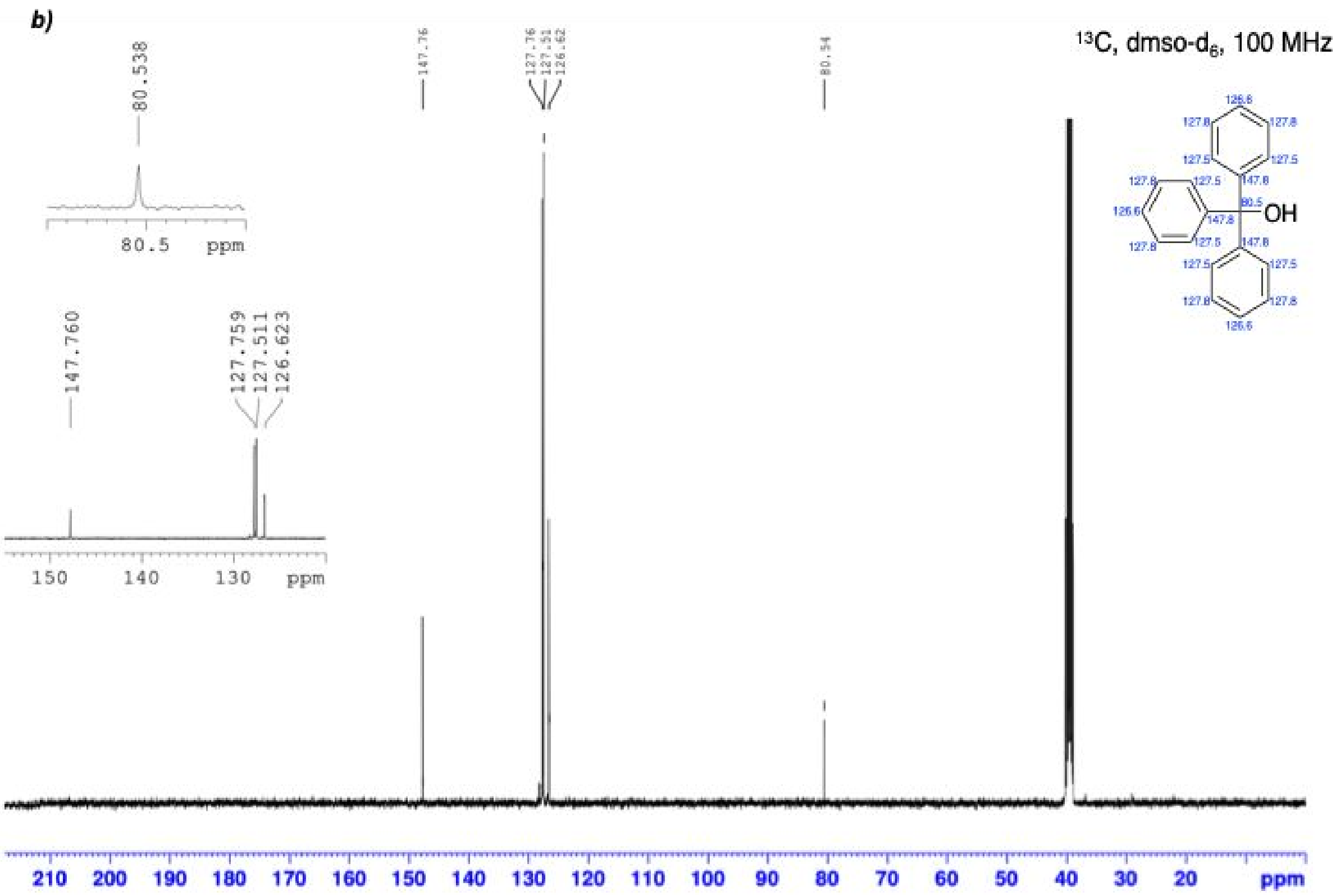

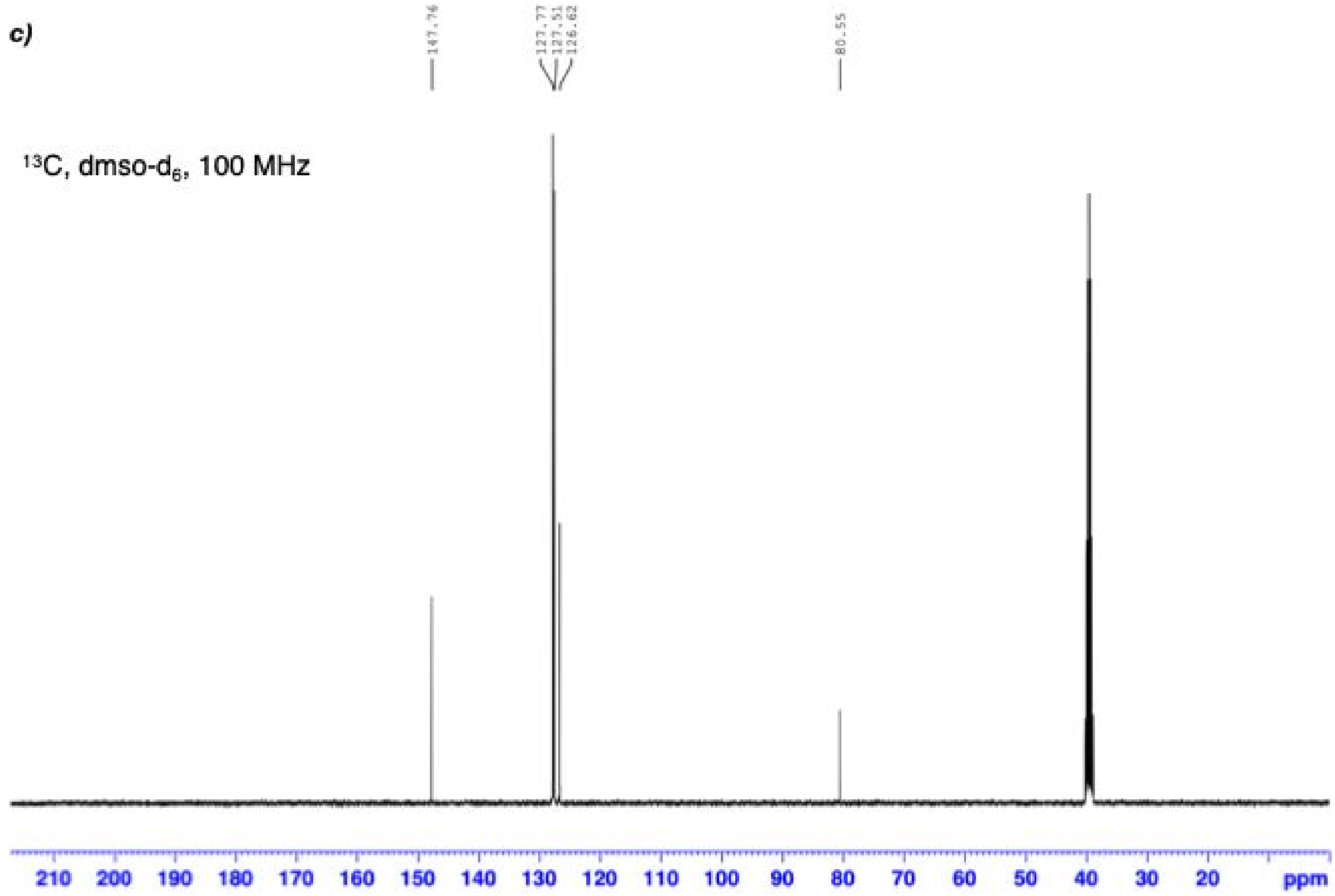

Figure S35. ^{1}H (400 MHz) (a) and ^{13}C (100 MHz) (b) NMR spectra of filtrates from entry **7** Table 3 (panels *a* and *b*). (c) ^{13}C (100 MHz) NMR spectrum of commercially available Trt-OH. All NMR spectra were recorded in DMSO-d_6.

*8. UV spectra of S-detritylation reaction mixture residue obtained from the protocol of entry **7**, Table 3*

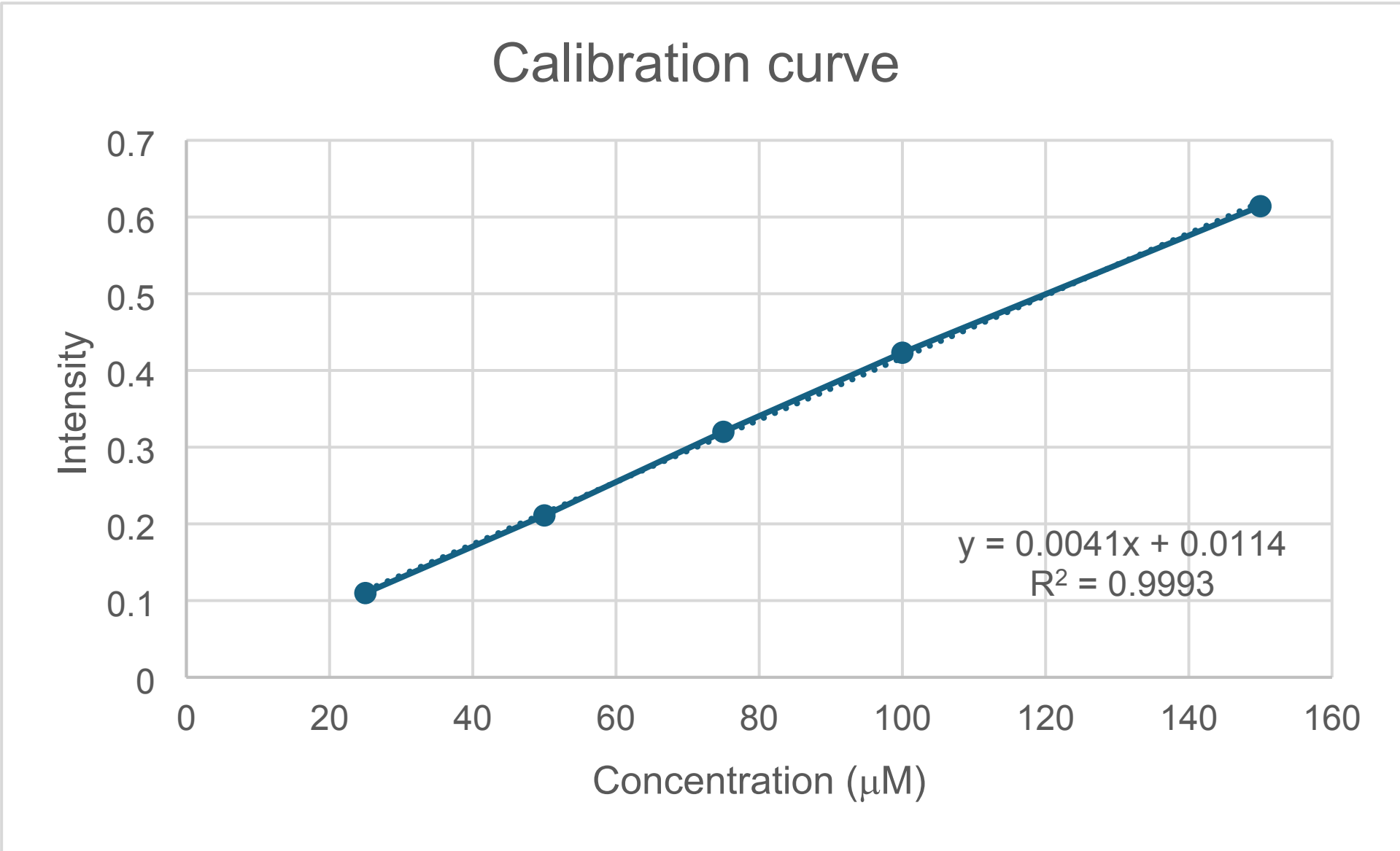

Figure S36. Calibration curve obtained by measuring the absorbance at 227.5 nm using a solution of Trt-OH, $AgCF_3CO_2$ (0.8 equiv) in DCM as a standard. The curve was constructed from a series of standard solutions at different concentrations (25, 50, 75, 100 and 150 mM). Linear regression analysis afforded the equation y= 0.0041x + 0.0114, with R^2= 0.9993.

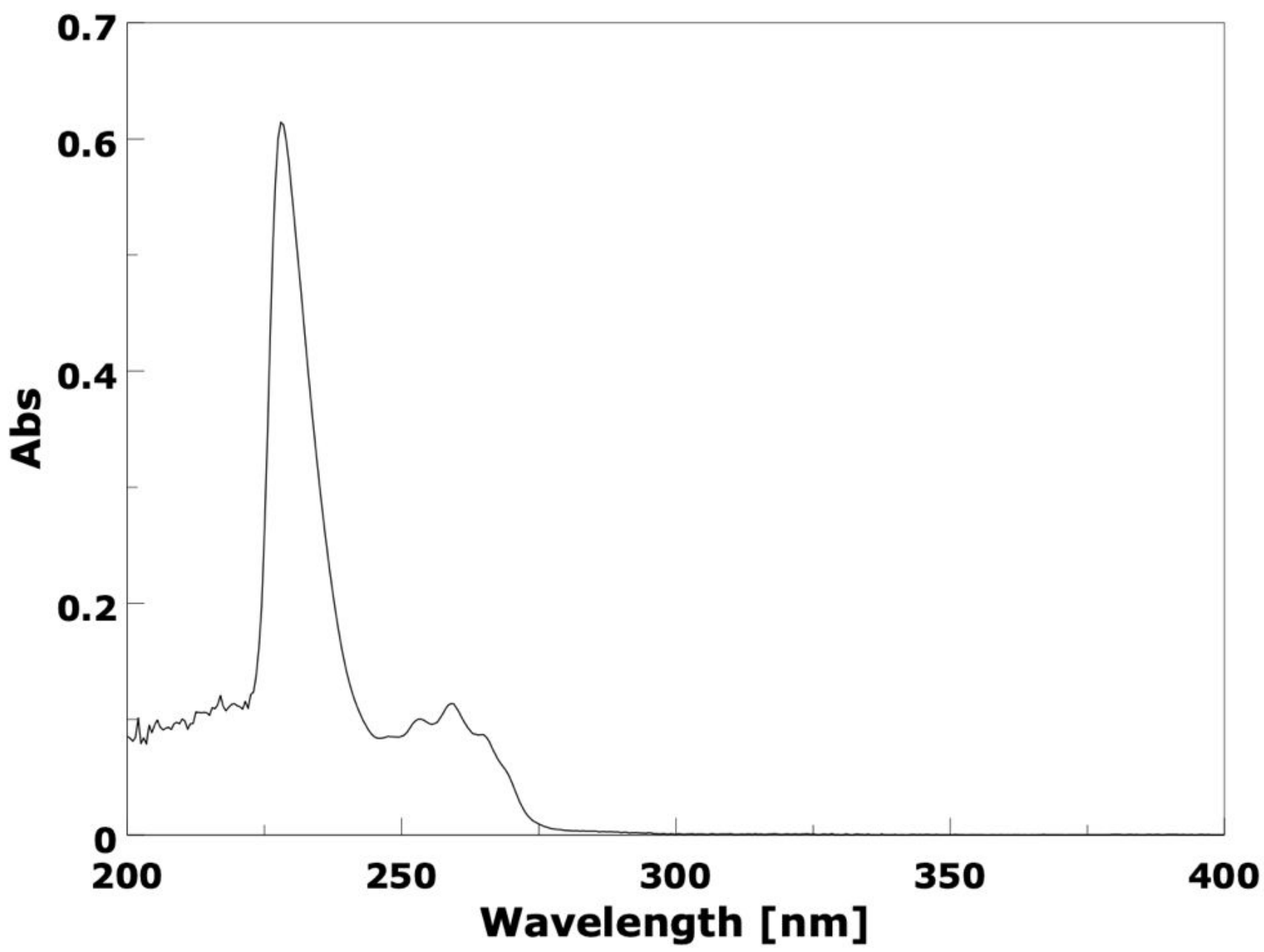

Figure S37. UV-Vis spectrum of the standard solution Trt-OH, $AgCF_3CO_2$ in DCM at 150 μM (0.6141 Abs). This concentration was chosen as it provides an absorbance value comparable to that measured for the analyzed sample.

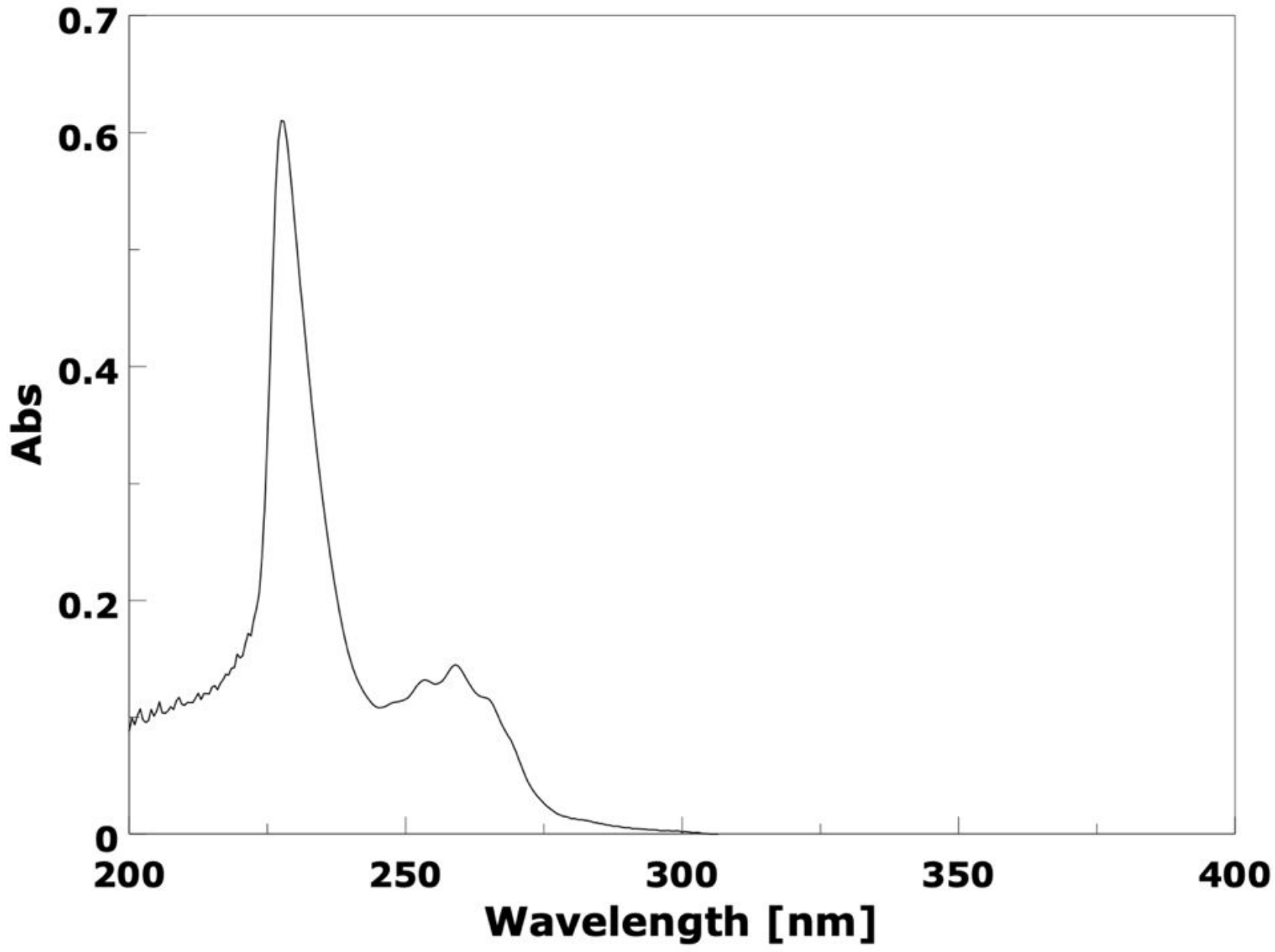

Figure S38. UV-Vis spectrum of filtrates solution from entry **7** Table 3 after *S*-Trt deprotection following the defined protocol, showing an absorbance of 0.6004 at 227.5 nm.

*9. HPLC chromatograms for Ag(CF_3CO_2) 0.2 equiv and entries **1-5**, Figure 6A*

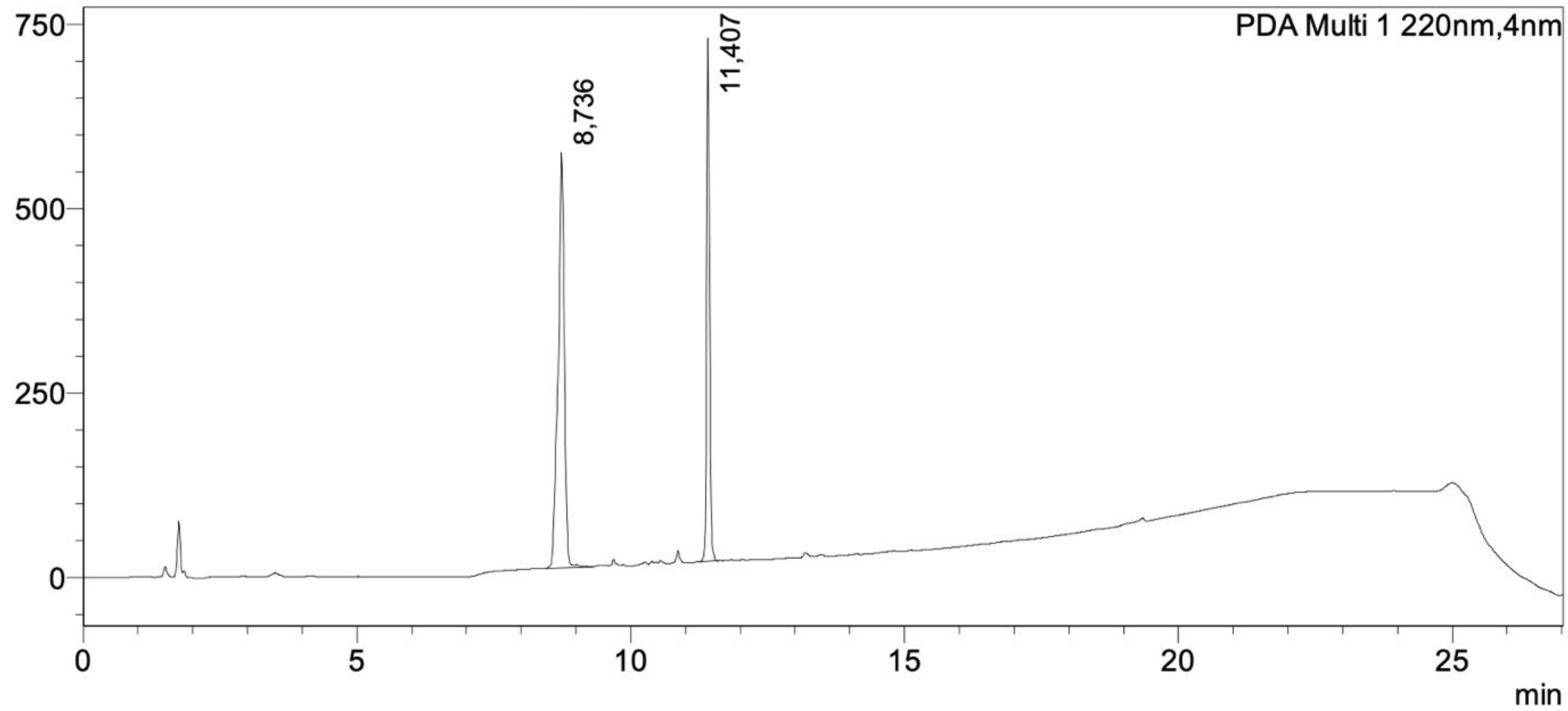

Figure S39. HPLC chromatogram obtained through the use of $Ag(CF_3CO_2)$ 0.2 equiv, showing 39% conversion to the *S*-benzylated product, t_R= 11.4 min. Analysis was performed using Method **B**, at a flow rate of 1 mL min^{-1}, with UV detection at 220 nm.

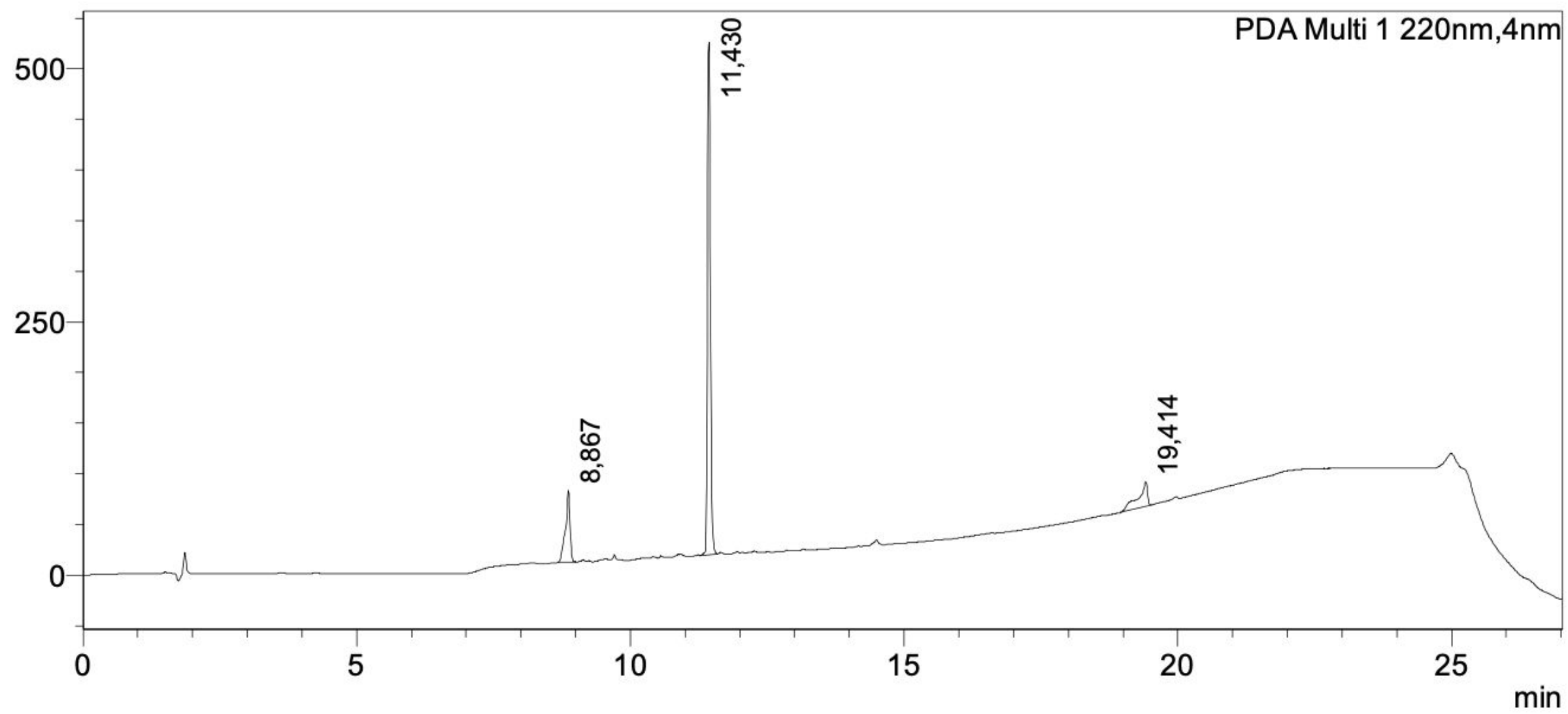

Figure S40. HPLC chromatogram of entry **1** (Figure 6A), showing 82% conversion to the *S*-benzylated product, t_R= 11.4 min. Analysis was performed using Method **B**, at a flow rate of 1 mL min^{-1}, with UV detection at 220 nm.

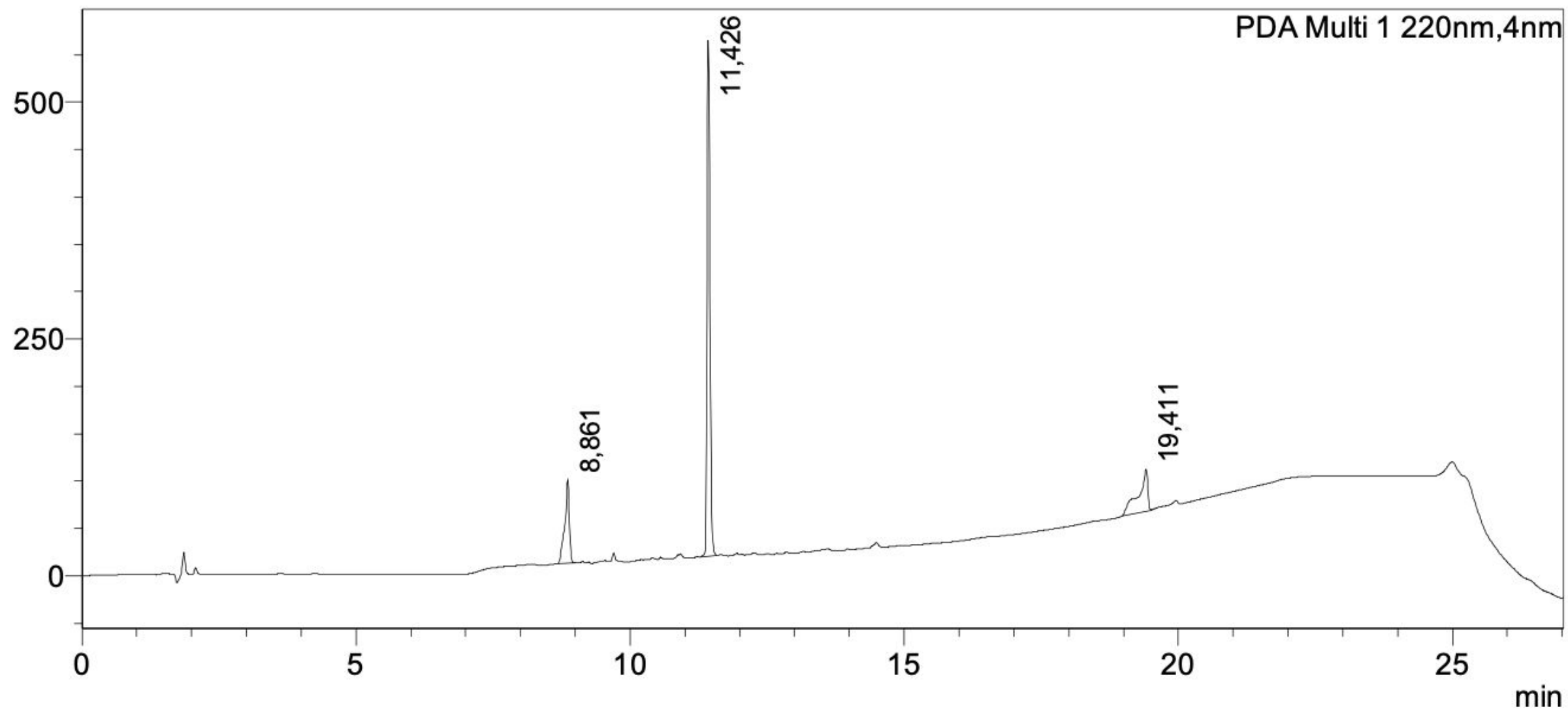

Figure S41. HPLC chromatogram of entry **2** (Figure *6A*), showing 80% conversion to the *S*-benzylated product, t_R= 11.4 min. Analysis was performed using Method **B**, at a flow rate of 1 mL min^{-1}, with UV detection at 220 nm.

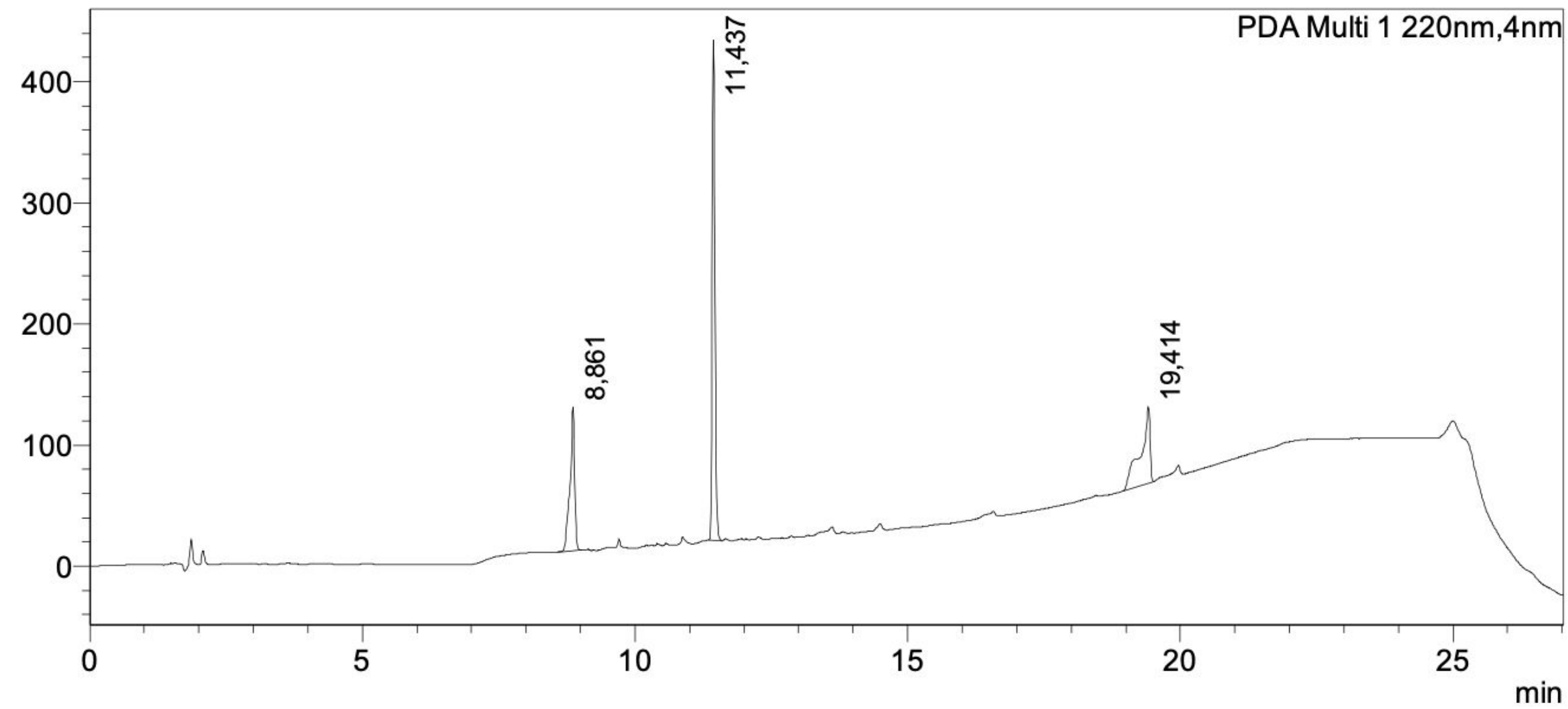

Figure S42. HPLC chromatogram of entry **3** (Figure *6A*), showing 68% conversion to the *S*-benzylated product, t_R= 11.4 min. Analysis was performed using Method **B**, at a flow rate of 1 mL min^{-1}, with UV detection at 220 nm.

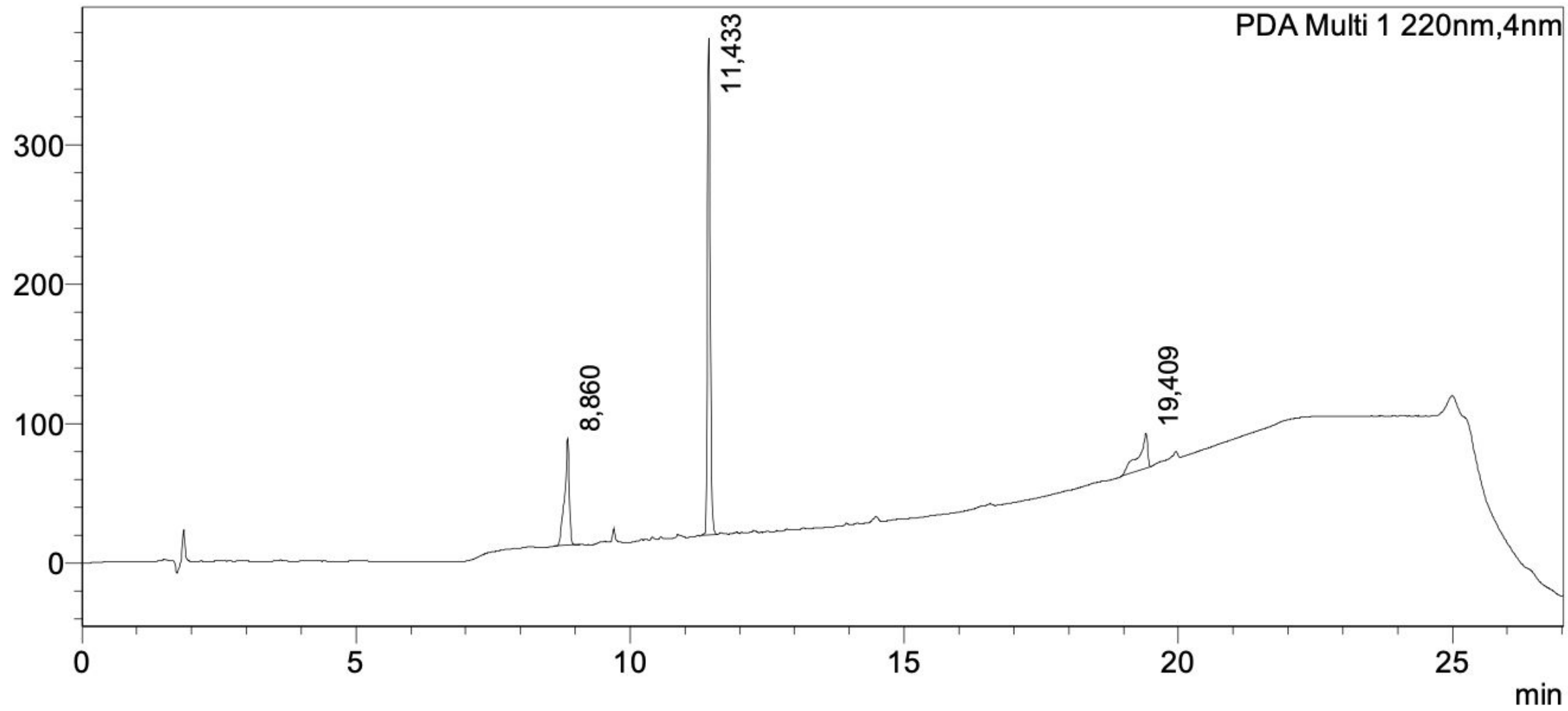

Figure S43. HPLC chromatogram of entry **4** (Figure *6A*), showing 73% conversion to the *S*-benzylated product, t_R= 11.4 min. Analysis was performed using Method **B**, at a flow rate of 1 mL min^{-1}, with UV detection at 220 nm.

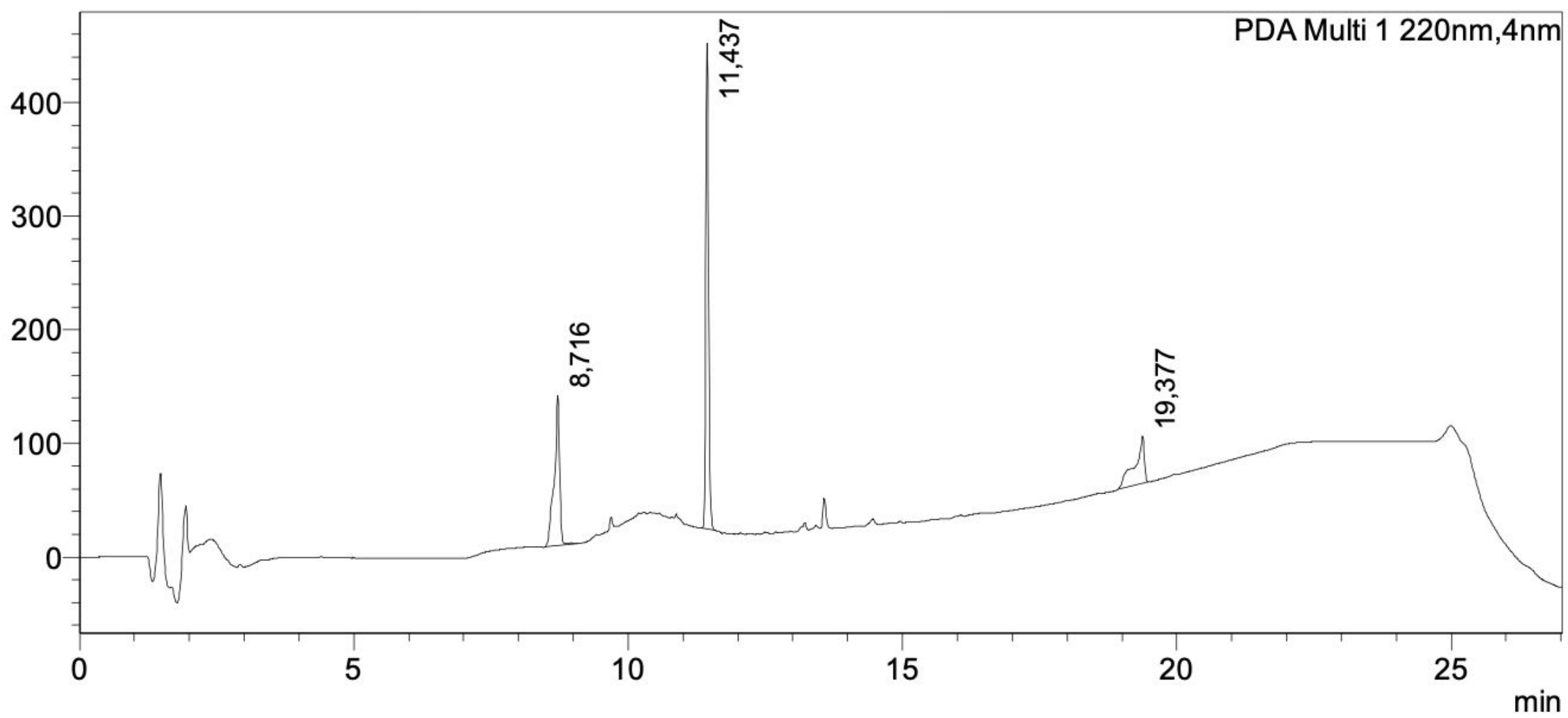

Figure S44. HPLC chromatogram of entry **5** (Figure 6A), showing 62% conversion to the *S*-benzylated product, t_R= 11.4 min. Analysis was perfomed using Method **B**, at a flow rate of 1 mL min^{-1}, with UV detection at 220 nm.

*10. HPLC chromatograms for entries **6-9**, Figure 6B*

Table S2. Screening of green solvents for *S*-detritylation, expressed as conversion to the *S*-benzylated product.

Green solvent	% Conv.
2-MeTHF	65±3
DMC	82±1
EtOAc	70±3
NBP	56±5

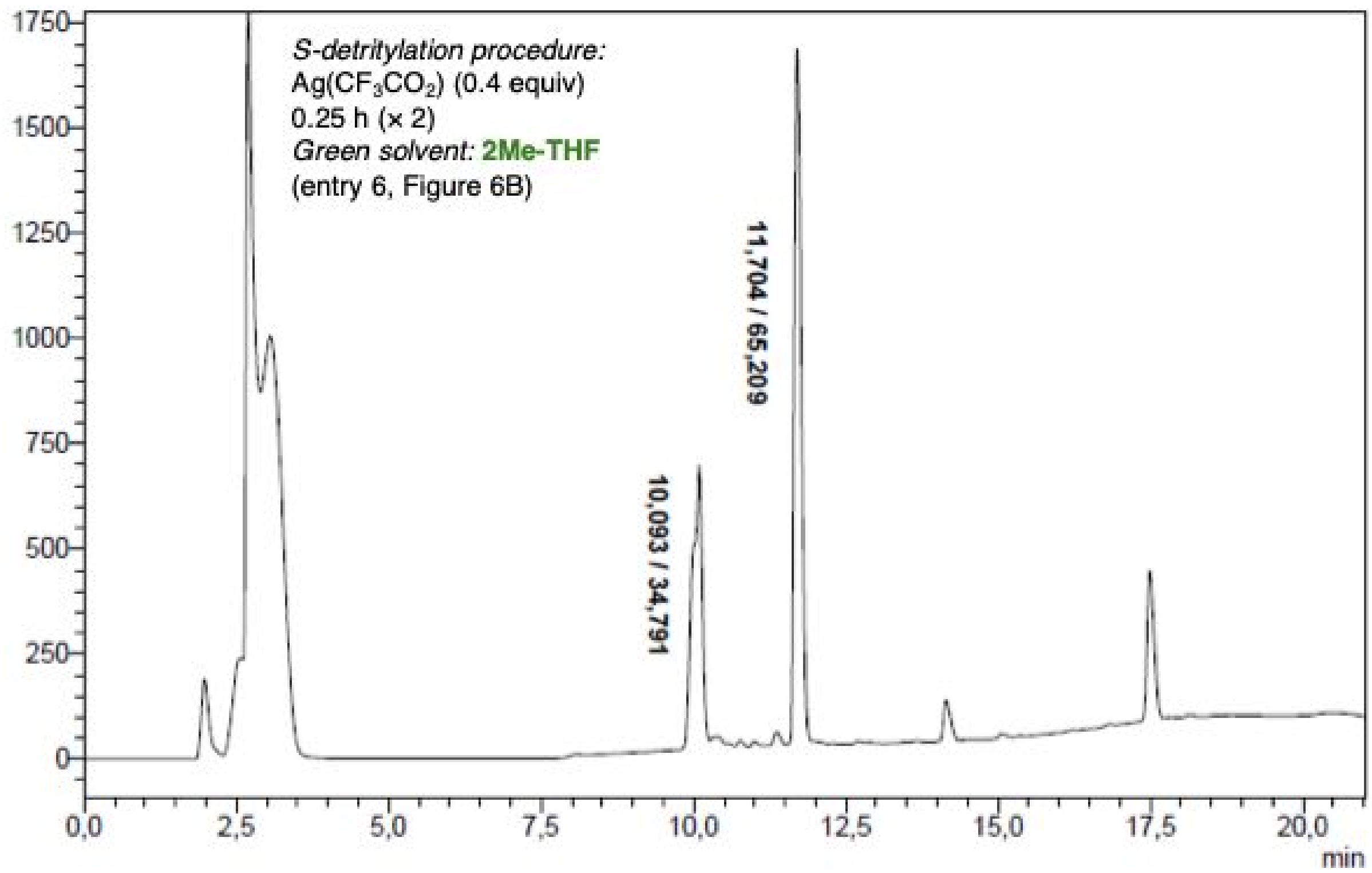

Figure S45. Representative HPLC chromatogram for entry **6** (Figure 6B) showing 65% conversion to the *S*-benzylated product, t_R= 11.7 min. Analysis was performed using Method **A**, at a flow rate of 1 mL min^{-1}, with UV detection at 220 nm.

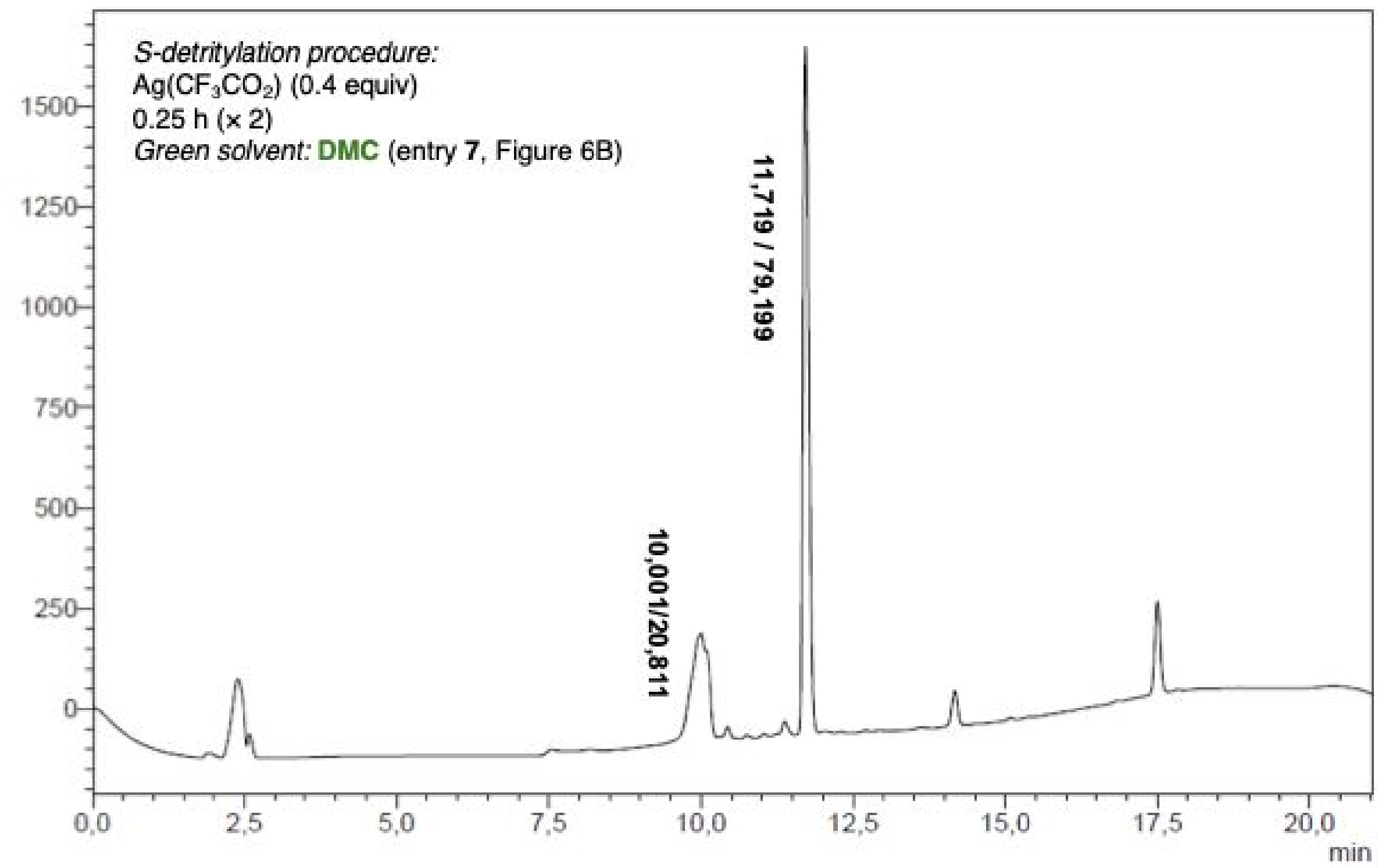

Figure S46. Representative HPLC chromatogram for entry **7** (Figure 6B) showing 79% conversion to the *S*-benzylated product, t_R= 11.7 min. Analysis was performed using Method **A**, at a flow rate of 1 mL min^{-1}, with UV detection at 220 nm.

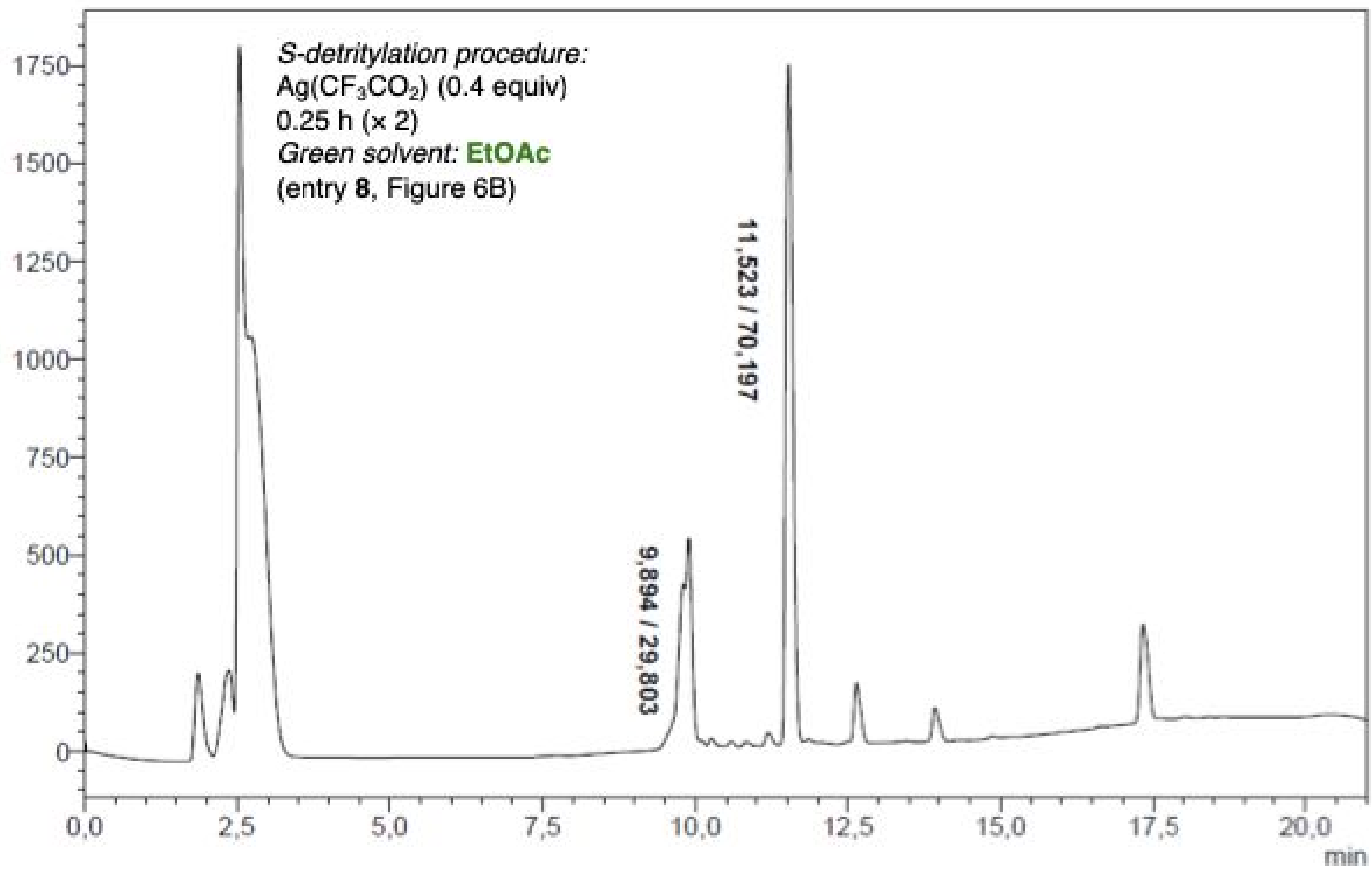

Figure S47. Representative HPLC chromatogram for entry **8** (Figure 6B) showing 70% conversion to the *S*-benzylated product, t_R= 11.5 min. Analysis was performed using Method **A**, at a flow rate of 1 mL min^{-1}, with UV detection at 220 nm.

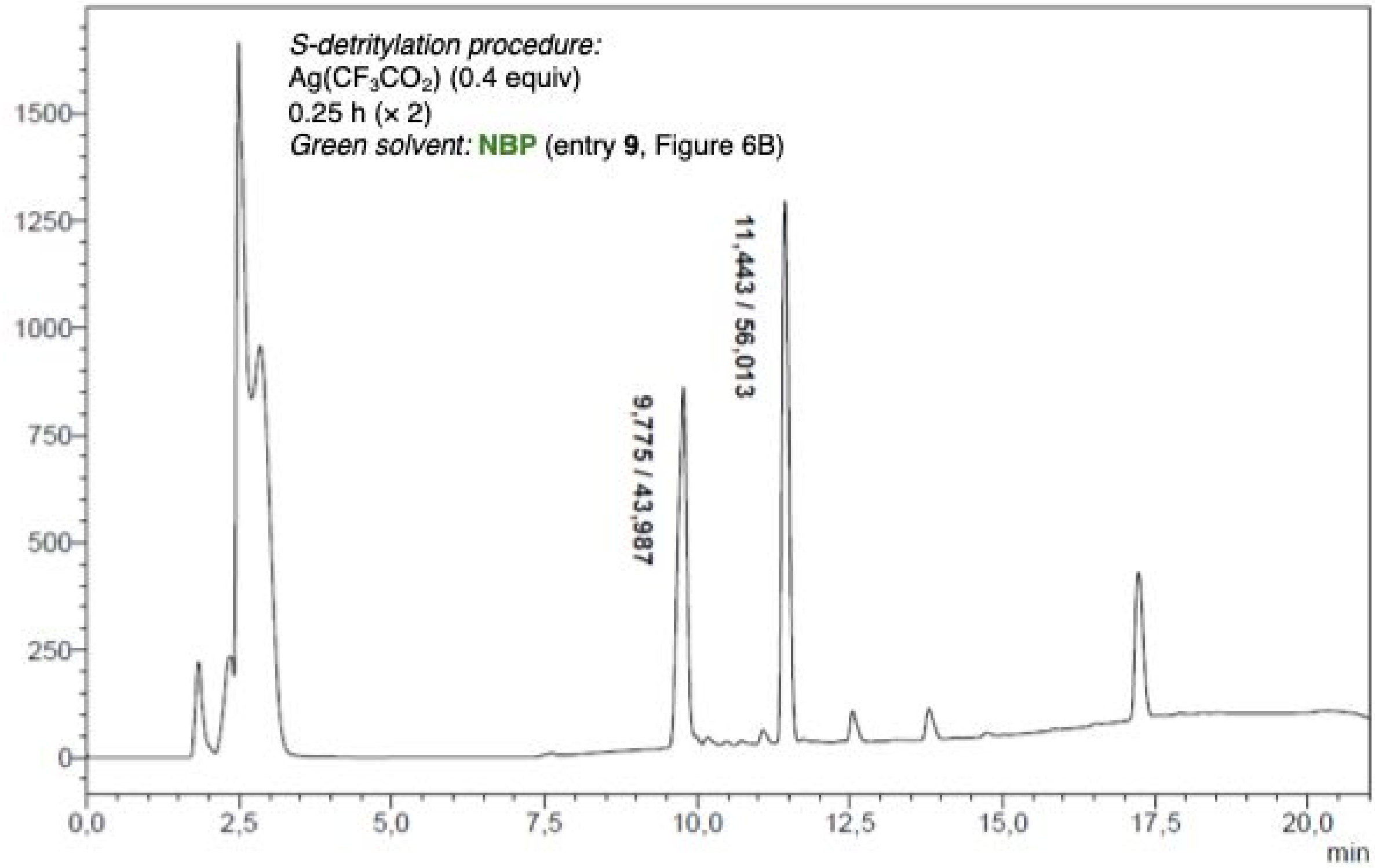

Figure S48. Representative HPLC chromatogram for entry **9** (Figure 6B) showing 56% conversion to the S-benzylated product, t_R= 11.4 min. Analysis was performed using Method A, at a flow rate of 1 mL min^{-1}, with UV detection at 220 nm.

*11. Analytical data for entries **10-13** of Figure 6B*

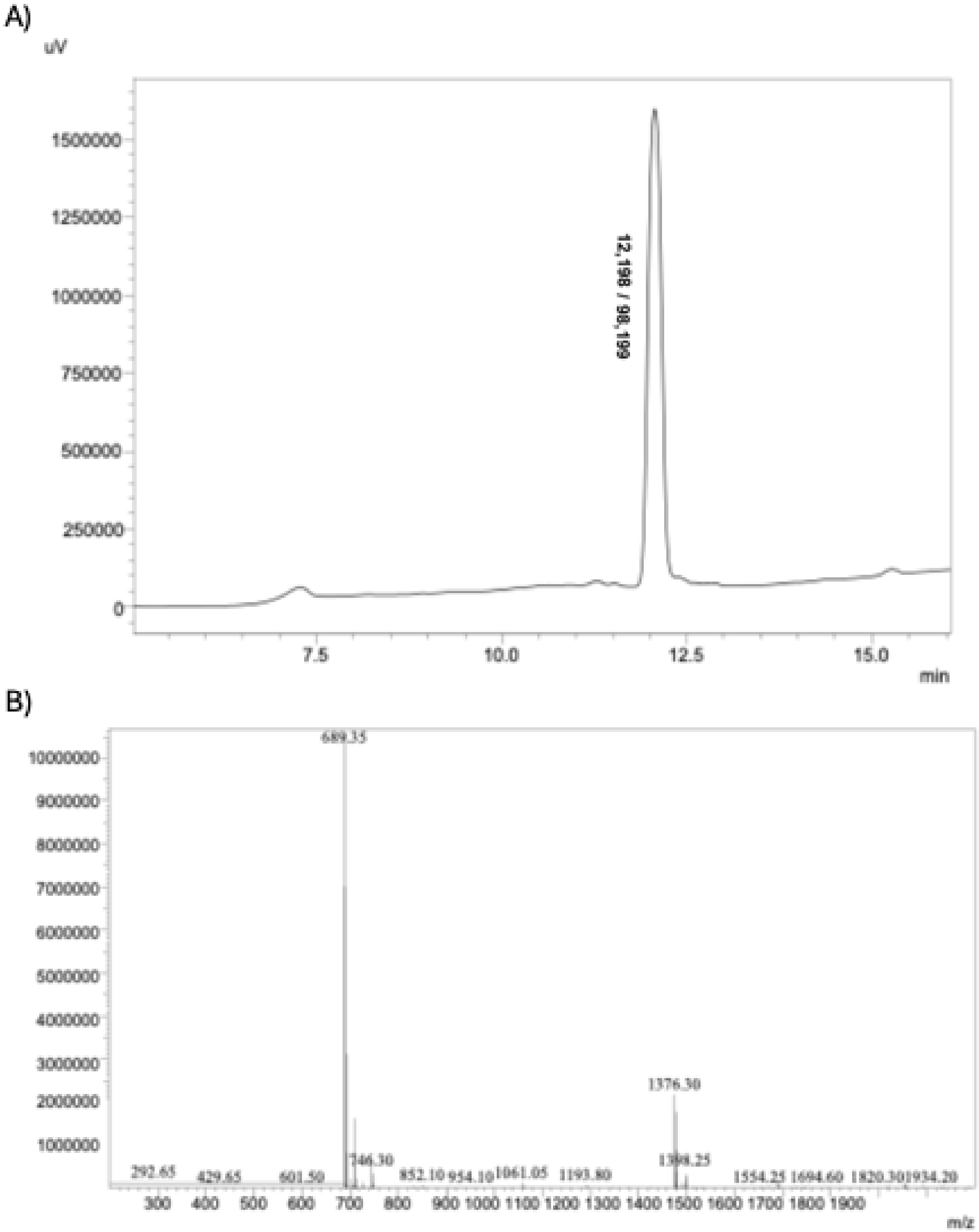

Figure S49. (A) Representative HPLC chromatogram of entry **10** (Figure 6B) obtained using Method **A**, at a flow rate of 1 mL min^{-1}, with UV detection at 220 nm. Retention time (t_R): 12.2 min. (B) Calculated mass for $[M+H]^+$: 688.83; found *m/z* 689.35; [M dimer $+H]^+$ 1376.30.

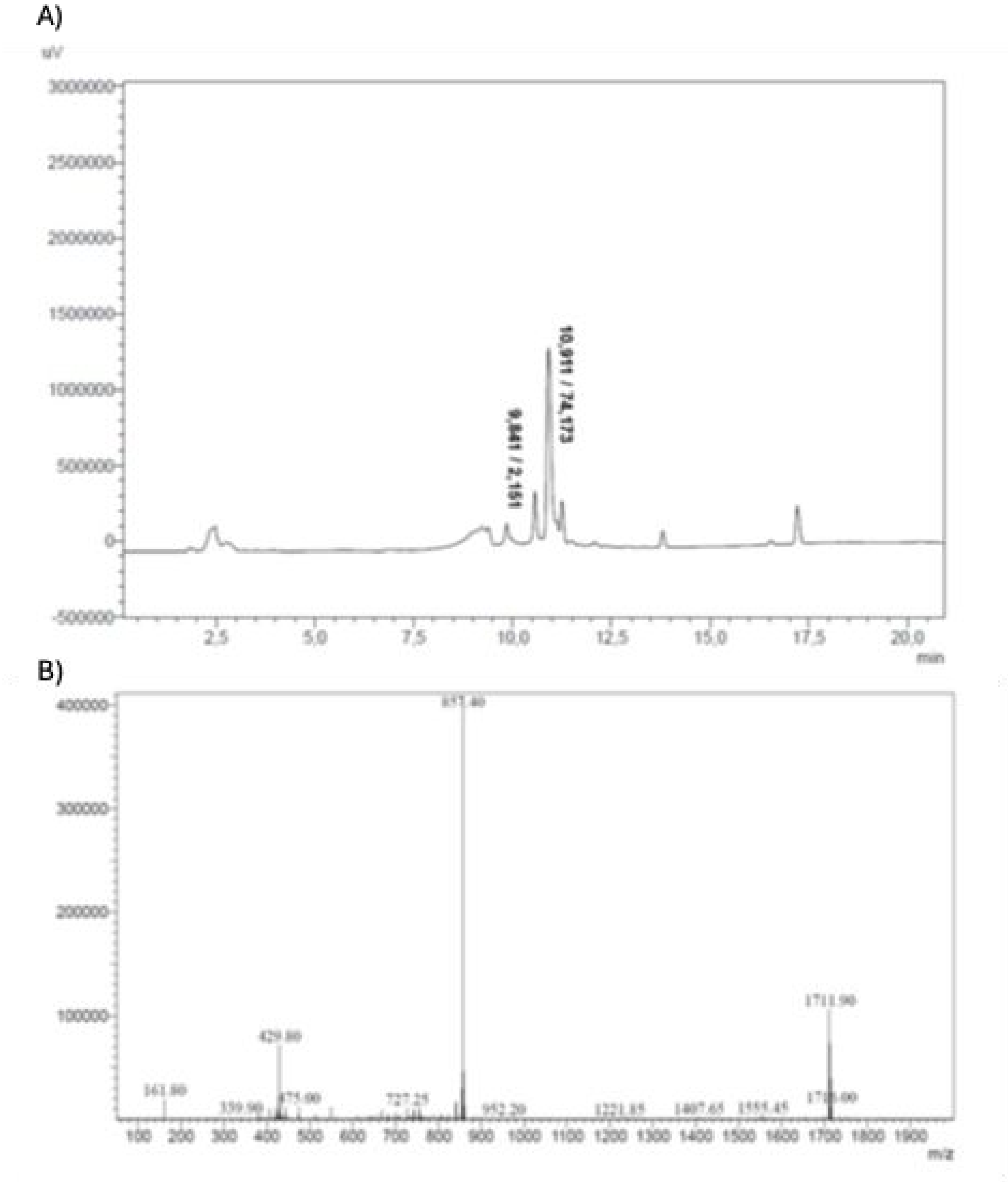

Figure S50. (A) Representative HPLC chromatogram of entry **11** (Figure 6B) obtained using Method **A**, at a flow rate of 1 mL min^{-1}, with UV detection at 220 nm. Retention time (t_R): 10.9 min. (B) Calculated mass for $[M+H]^+$: 856.37; found *m/z* 857.4; [M dimer +H]$^+$ 1711.9; $[M+2/2]^+$ 429.8.

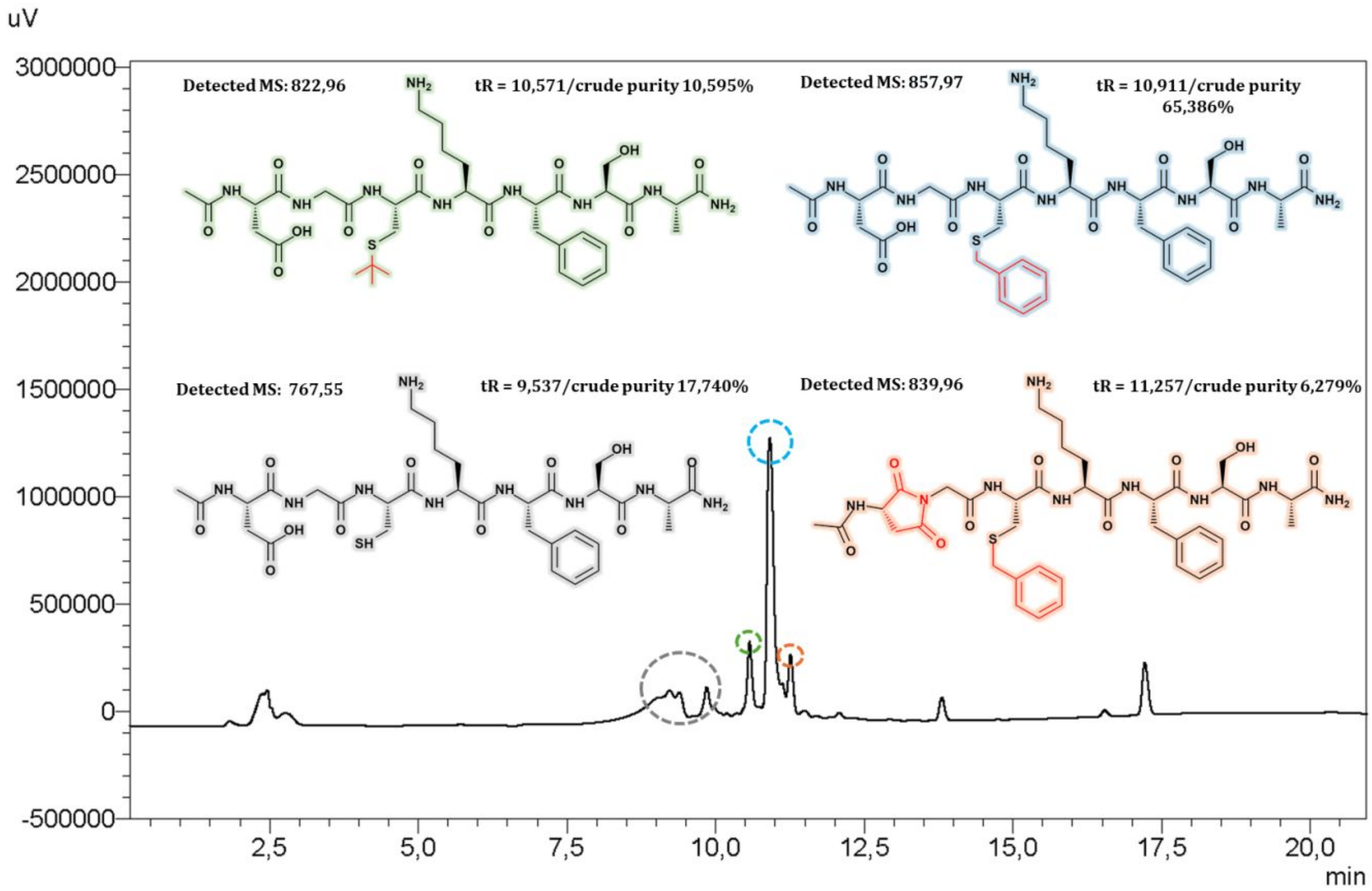

Figure S51. HPLC profile of entry **11** (Figure 6B), including the structure, crude purity, t_R and detected masses of the main products obtained from the reaction. The analysis was carried out using Method **A**, with a flow rate of 1 mL min^{-1} and UV detection at 220 nm.

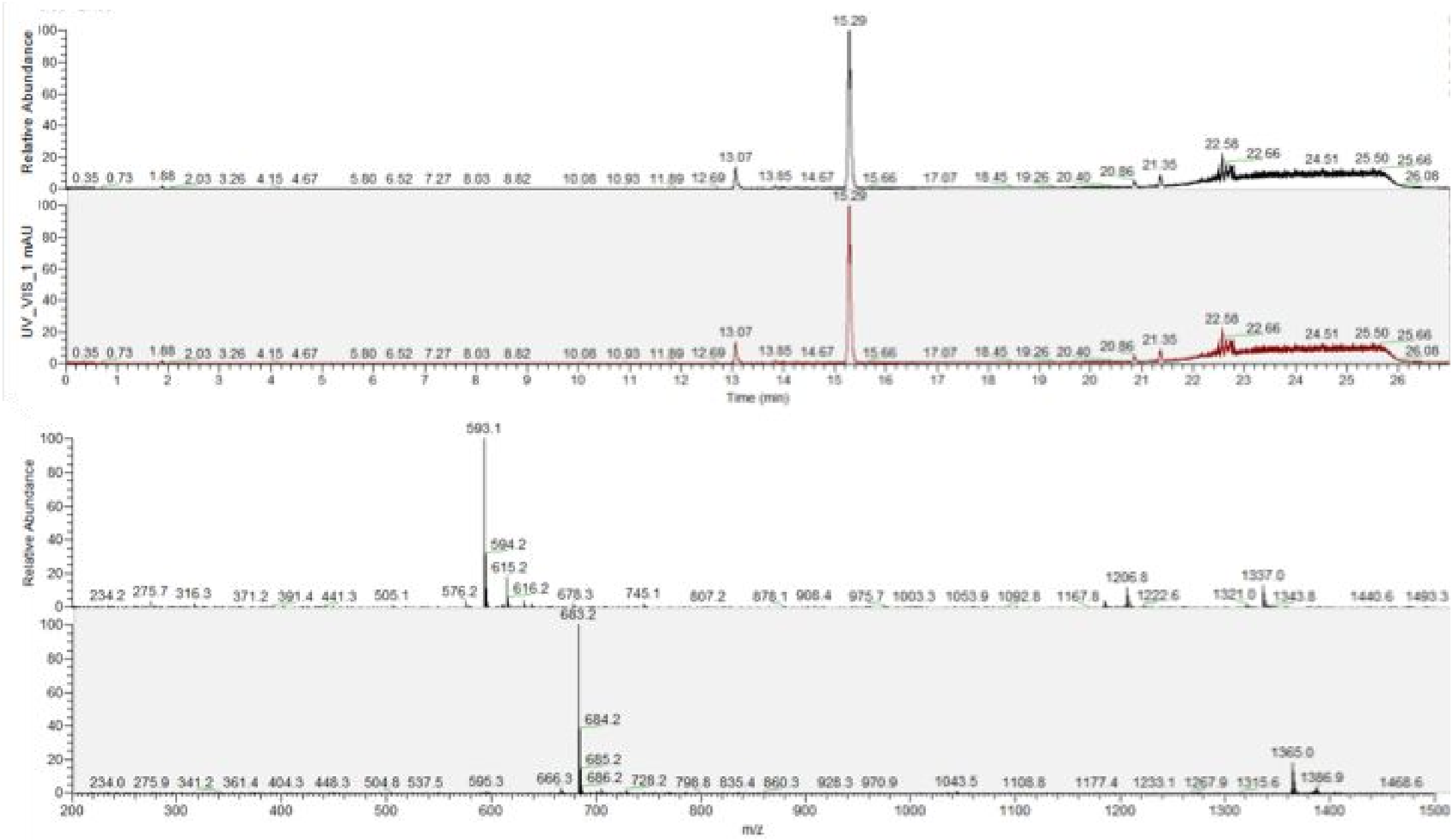

Figure S52. LC-MS chromatogram of entry **12** (Figure 6B) was performed using Method **B**, at a flow rate of 0.8 mL min^{-1}, with UV detection at 220 nm, t_R= 15.3 min.

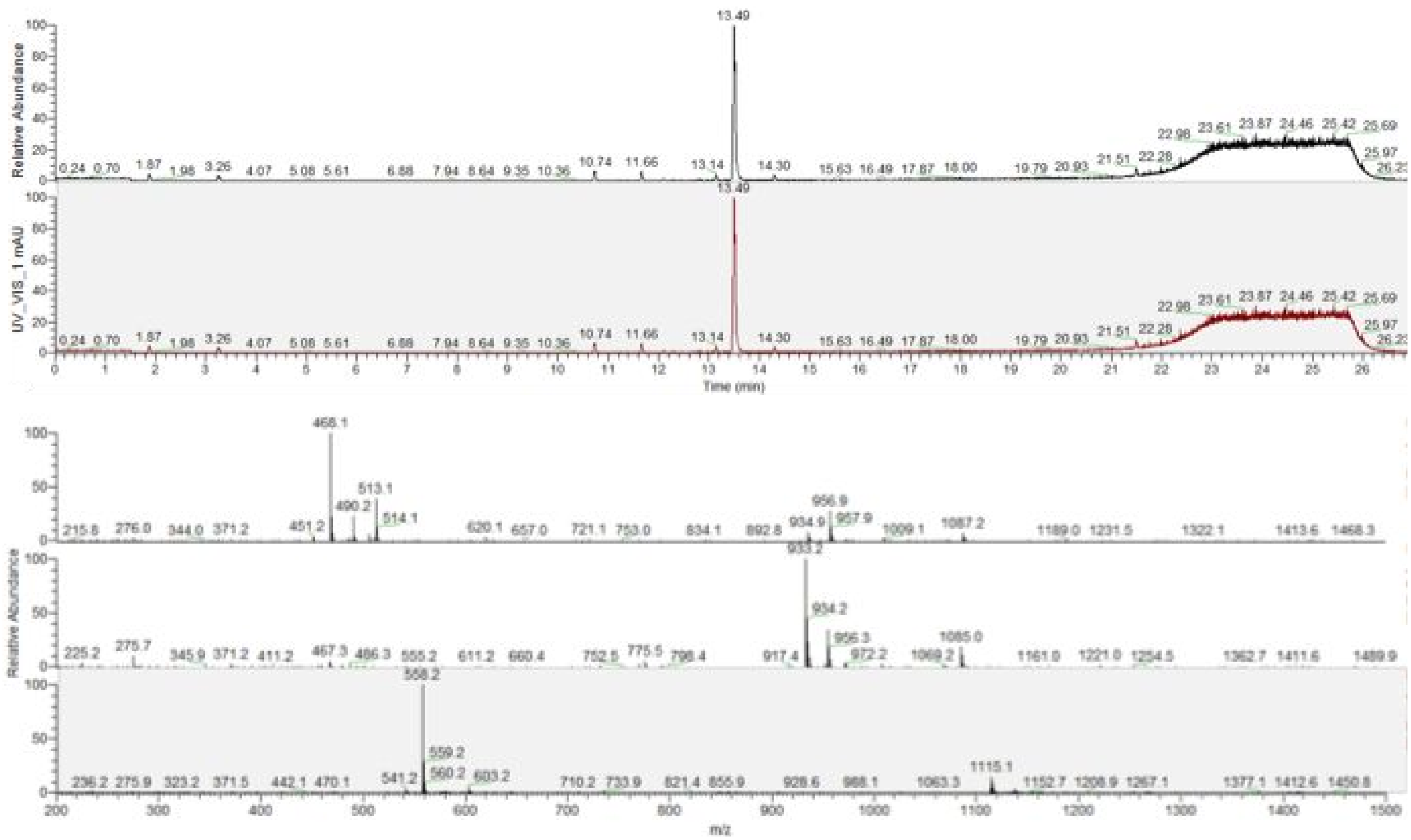

Figure S53. LC-MS chromatogram of entry **13** (Figure 6B) was performed using Method **B**, at a flow rate of 0.8 mL min^{-1}, with UV detection at 220 nm, t_R= 13.5 min.

*12. Analytical data for entries **1-6** of Figure **7***

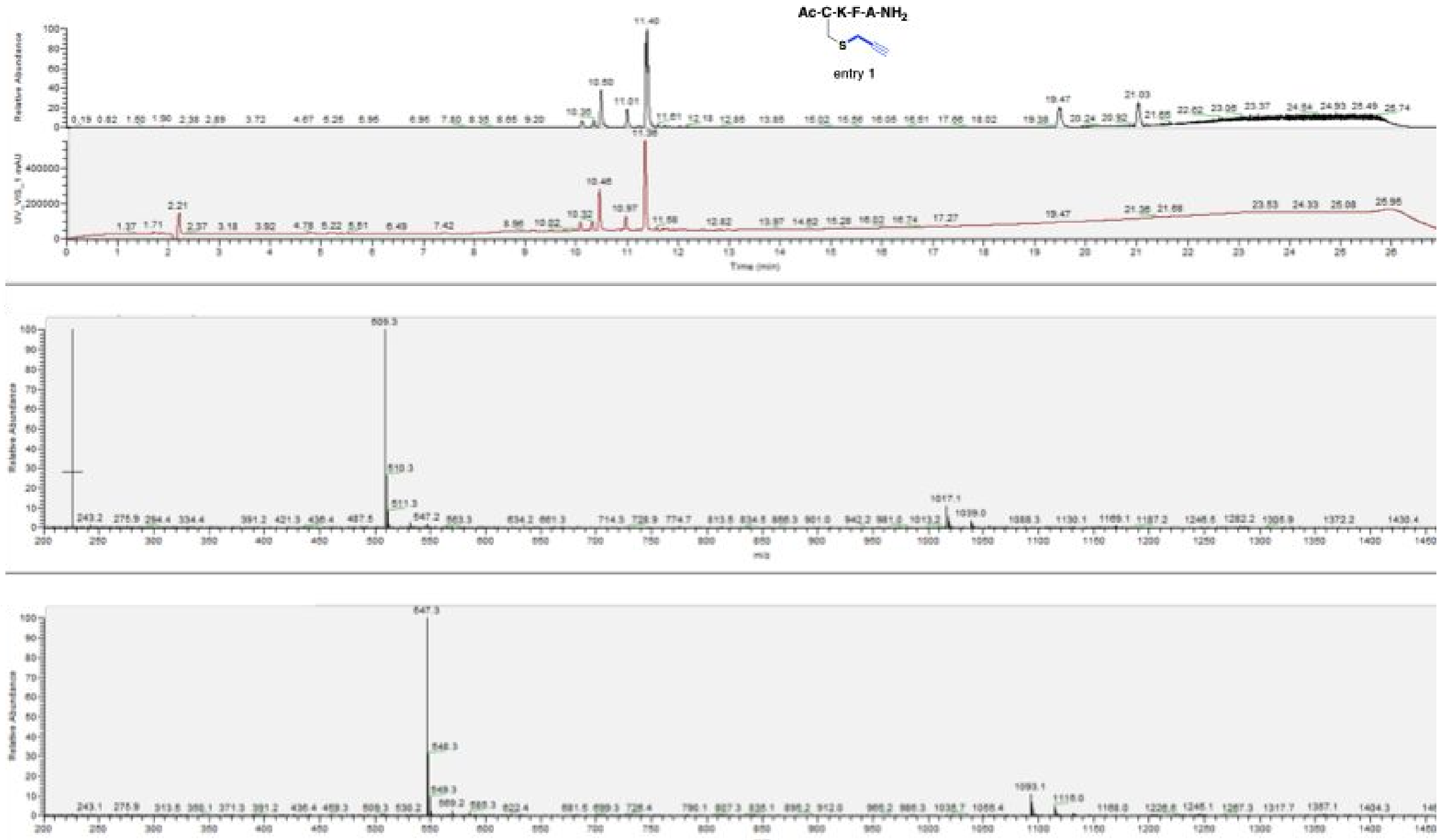

Figure S54. LC-MS chromatogram of entry **1** (Figure 7) was performed using Method **B**, at a flow rate of 0.8 mL min^{-1}, with UV detection at 220 nm, t_R= 11.4 min.

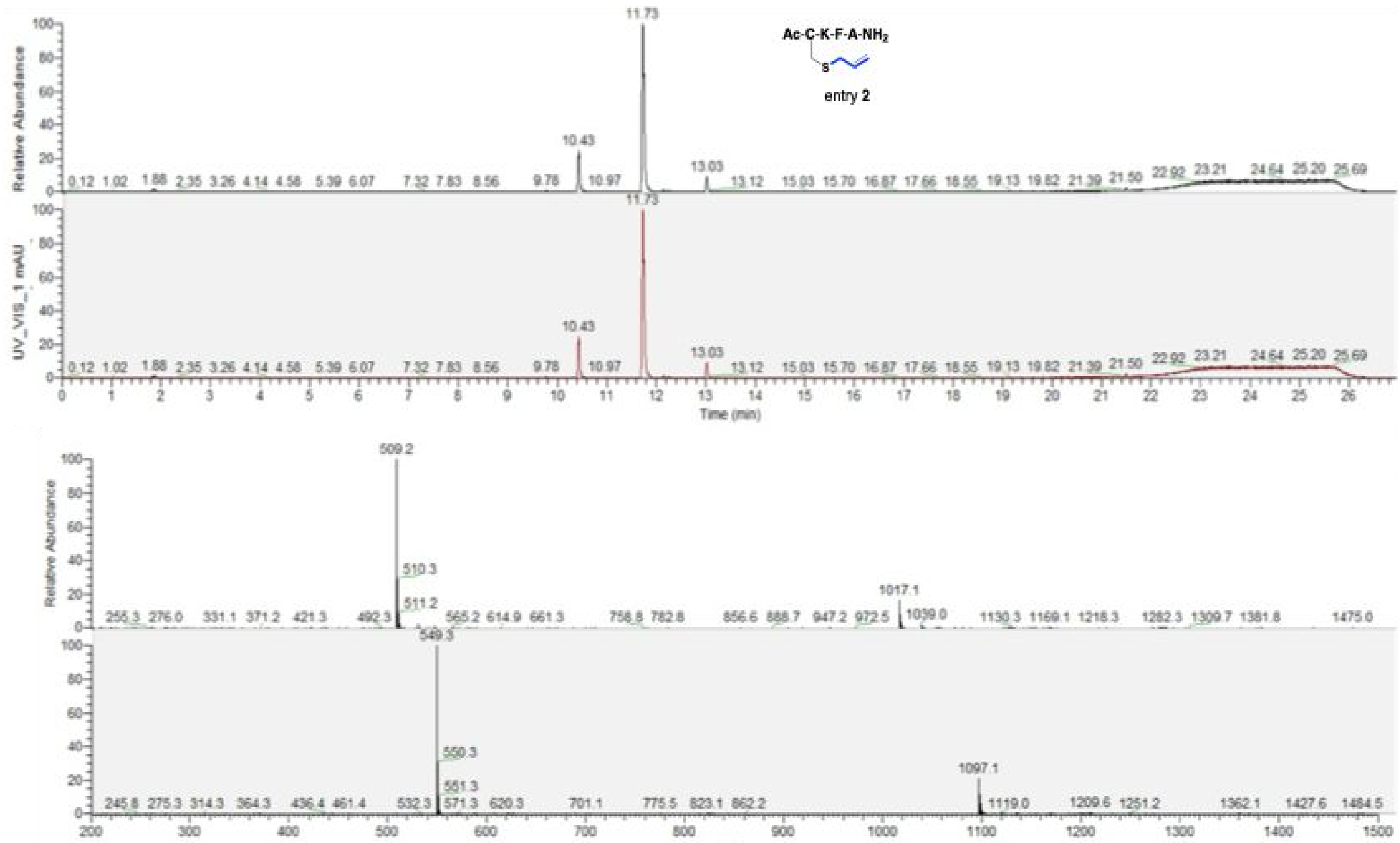

Figure S55. LC-MS chromatogram of entry **2** (Figure 7) was performed using Method **B**, at a flow rate of 0.8 mL min^{-1}, with UV detection at 220 nm, t_R= 11.7 min.

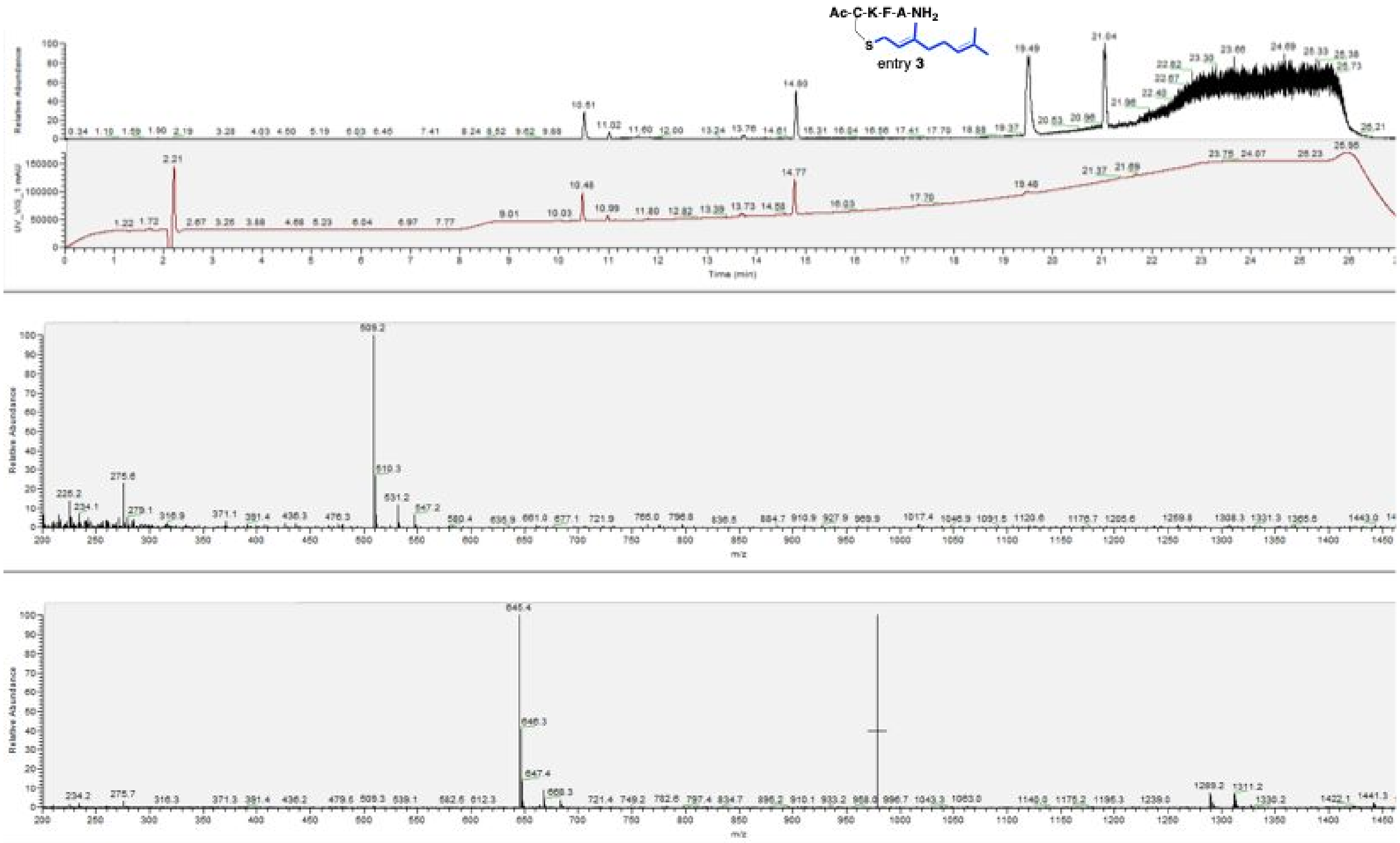

Figure S56. LC-MS chromatogram of entry **3** (Figure 7) was performed using Method **B**, at a flow rate of 0.8 mL min^{-1}, with UV detection at 220 nm, t_R= 14.8 min.

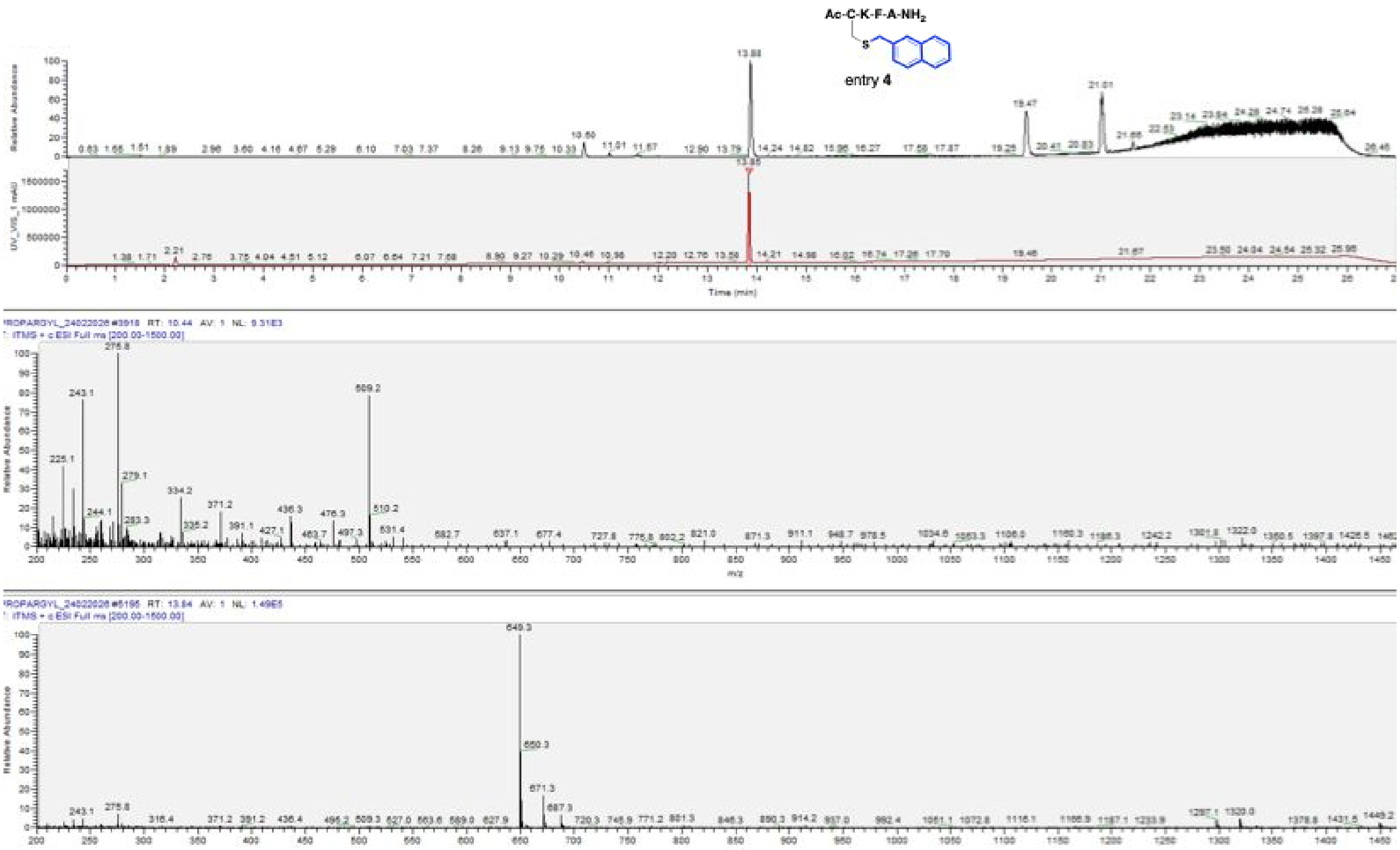

Figure S57. LC-MS chromatogram of entry **4** (Figure 7) was performed using Method **B**, at a flow rate of 0.8 mL min^{-1}, with UV detection at 220 nm, t_R= 13.9 min.

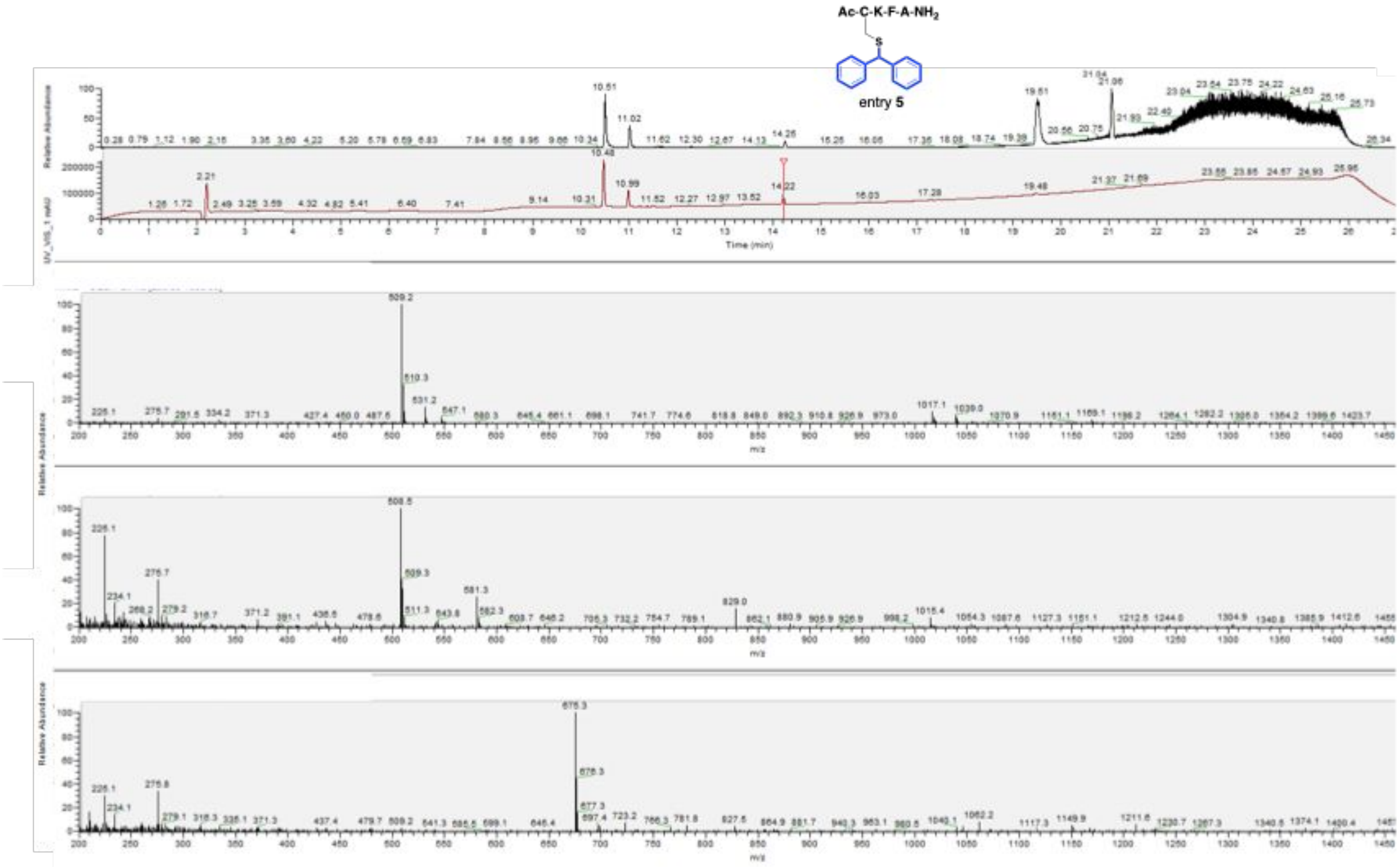

Figure S58. LC-MS chromatogram of entry **5** (Figure 7) was performed using Method **B**, at a flow rate of 0.8 mL min^{-1}, with UV detection at 220 nm, t_R= 14.2 min.

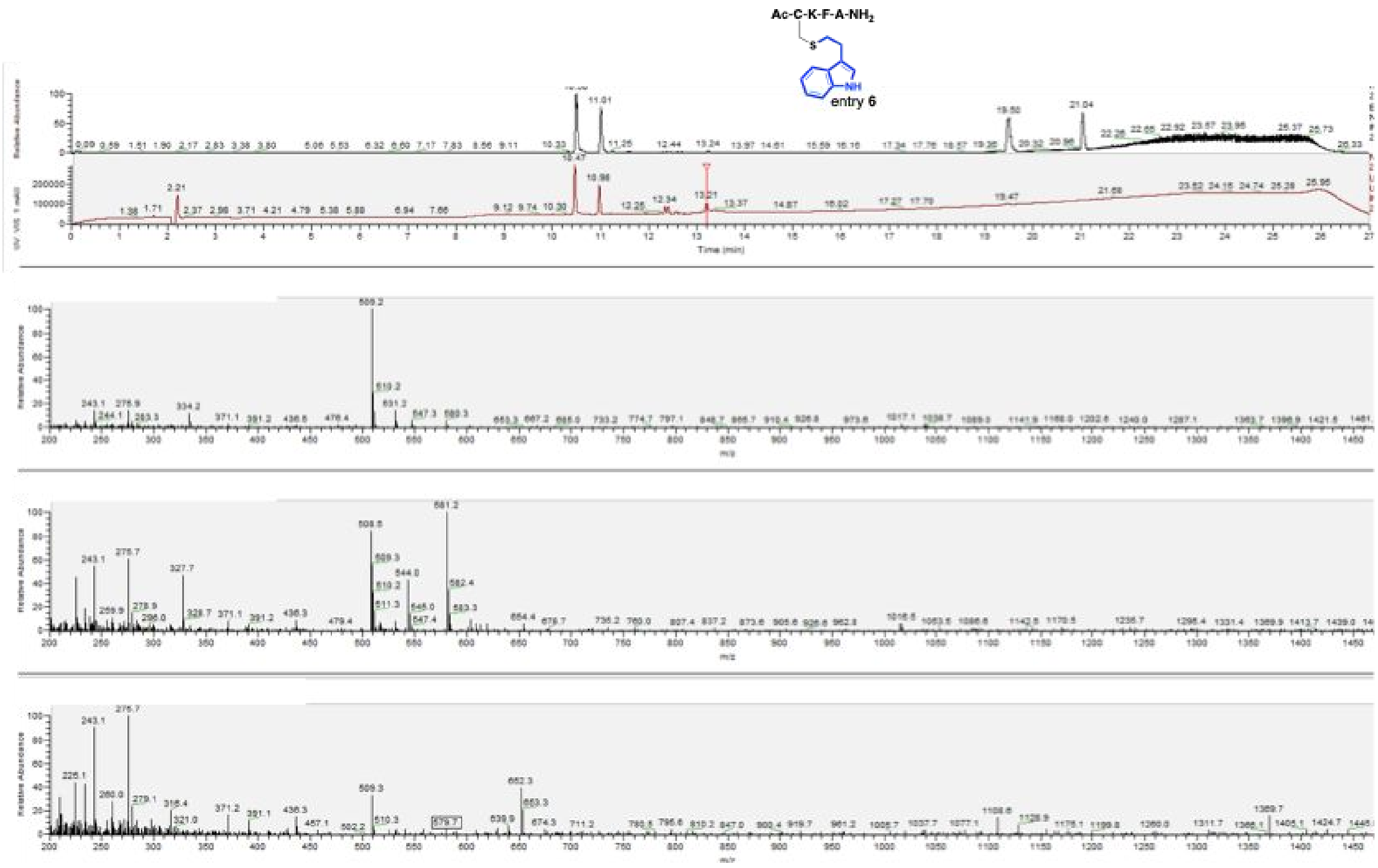

Figure S59. LC-MS chromatogram of entry **6** (Figure 7) was performed using Method **B**, at a flow rate of 0.8 mL min^{-1}, with UV detection at 220 nm, t_R= 13.2 min.
